# Supplementary material for: Simulations predict preferred Mg2+ coordination in a nonenzymatic primer-extension reaction center
Source: Biophys J. 2024 May 3;123(12):1579–91. doi: 10.1016/j.bpj.2024.04.032 (PMC11214020; doi:10.1016/j.bpj.2024.04.032)
Supplement: Document S1. Figures S1–S20, Tables S1, and S2 [file mmc1.pdf]

Biophysical Journal, Volume *123*

Supplemental information

Simulations predict preferred Mg^{2+} coordination in a nonenzymatic primer-extension reaction center

Shriyaa Mittal, Collin Nisler, and Jack W. Szostak

Supplemental Information

Simulations predict preferred Mg^{2+} coordination in a nonenzymatic primer extension reaction center

Shriyaa Mittal,[1,2,5] Collin Nisler,[4,5] Jack W. Szostak[,1,2,3,4]*

[1]*Howard Hughes Medical Institute, Department of Molecular Biology, and Center for Computational and Integrative Biology, Massachusetts General Hospital, Boston, MA, 02114 USA;* [2]*Department of Genetics, Harvard Medical School, Boston, MA, 02115 USA;* [3]*Department of Chemistry and Chemical Biology, Harvard University, Cambridge, MA, 02138 USA;* [4]*Howard Hughes Medical Institute, Department of Chemistry, University of Chicago, Chicago, IL, 60637 USA*

[5] These authors contributed equally to this work

* Corresponding author – Jack Szostak, jwszostak@uchicago.edu

Supplemental Figures

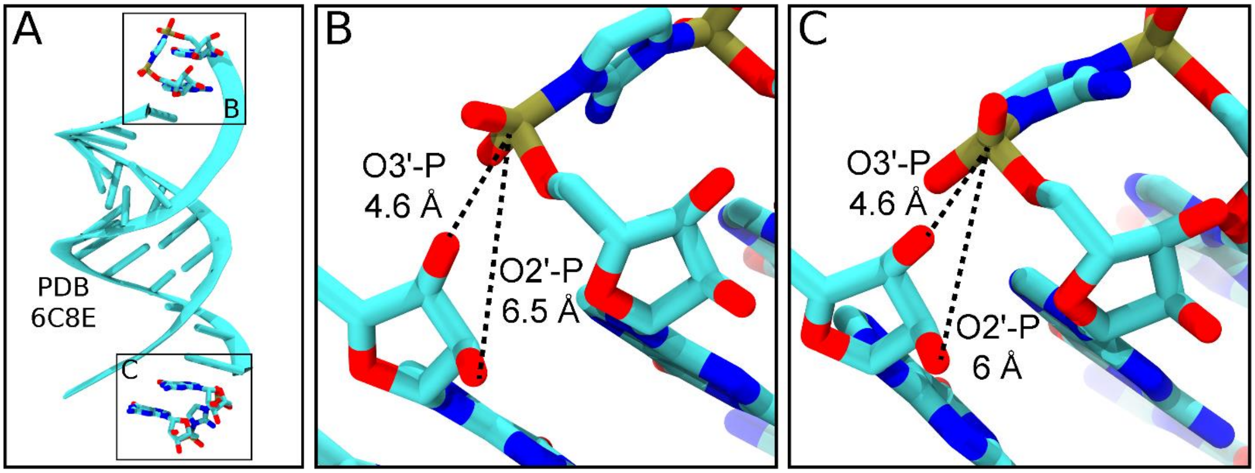

Figure S1. Distances observed in the crystal structure PDB 6C8E used for constraints during equilibration. (A) Structure of PDB 6C8E with RNA duplex shown in cartoon representation, and the 2AI-bridged dimer in licorice representation. (B) Zoomed in view from (A) showing O3'-P and O2'-P distances when 2AI is facing the major groove. (C) Zoomed in view from (A) showing O3'-P and O2'-P distances when 2AI is facing the minor groove.

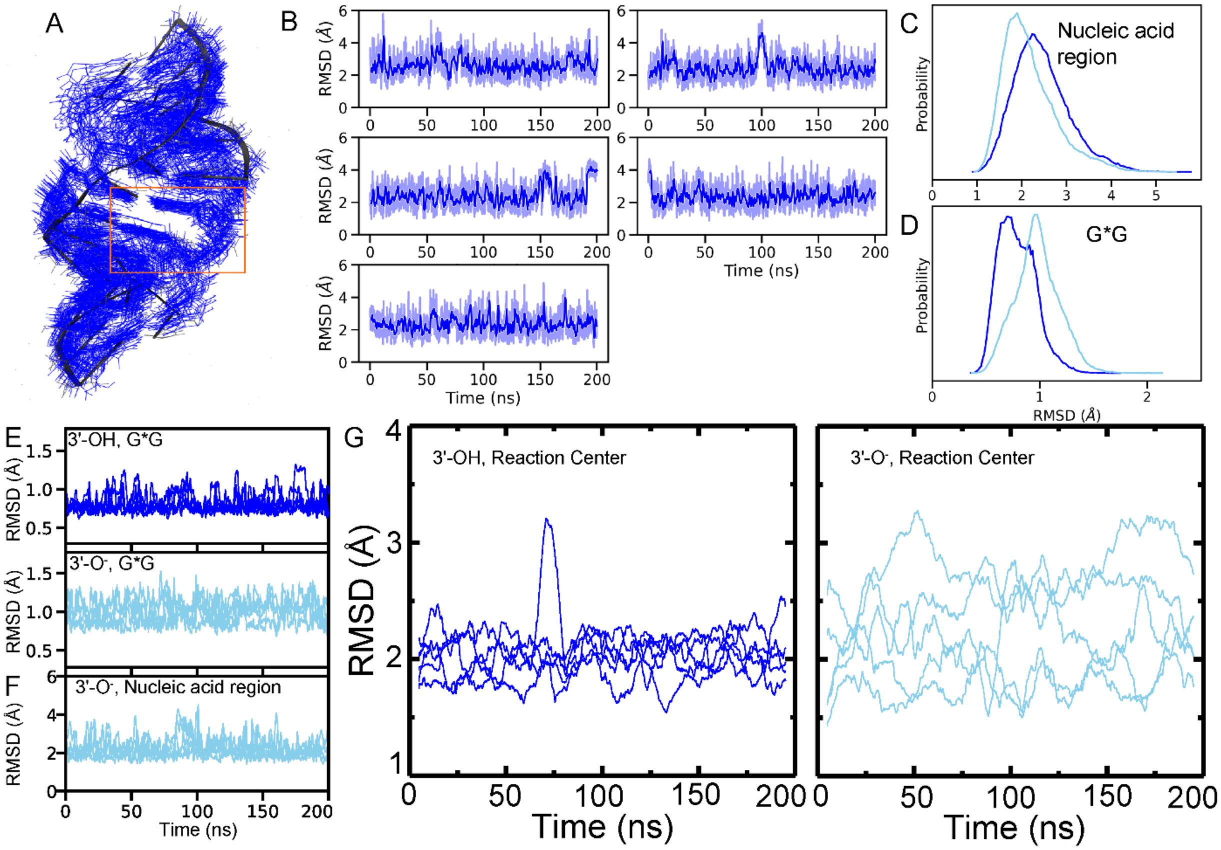

Figure S2. **RMSD of the duplex, bridged dinucleotide, and reaction center suggest increased dynamics with a deprotonated 3′-OH.** (A) Overlay of simulation snapshots over a 500 ns simulation trajectory of the complex with primer 3′-OH w/o Mg^{2+}. The first frame of the simulation can be seen partly as a dark gray ribbon representation. The G*G bridged dinucleotide is highlighted in an orange box. (B) Time series of root mean-squared deviation (RMSD) of the entire duplex for five simulation replicates. Comparing RMSD probability distribution for the (C) nucleic acid region and (D) G*G bridged dinucleotide (2AI-bridged dinucleotide only) for the simulation systems, 3′-OH w/o Mg^{2+} (Sim1; dark blue) and 3′O⁻ w/o Mg^{2+} (Sim2; light blue). (E) Time series of the G*G dinucleotide RMSD of the 3′-OH w/o Mg^{2+} (Sim1a-e; left) and 3′O⁻ w/o Mg^{2+} (Sim2a-e; right) simulation systems for all simulation replicates showing the 1 ns running averages. (F) Time series of the nucleic acid region RMSD of the 3′O⁻ w/o Mg^{2+} (Sim2a-e) simulation system for all simulation replicates showing the 1 ns running averages. (G) Time series of the reaction center RMSD, comprised of the G*G bridged dinucleotide and 3′-terminal primer guanosine, of the 3′OH w/o Mg^{2+} (Sim1; left), and the 3′O⁻ w/o Mg^{2+} (Sim2; right).

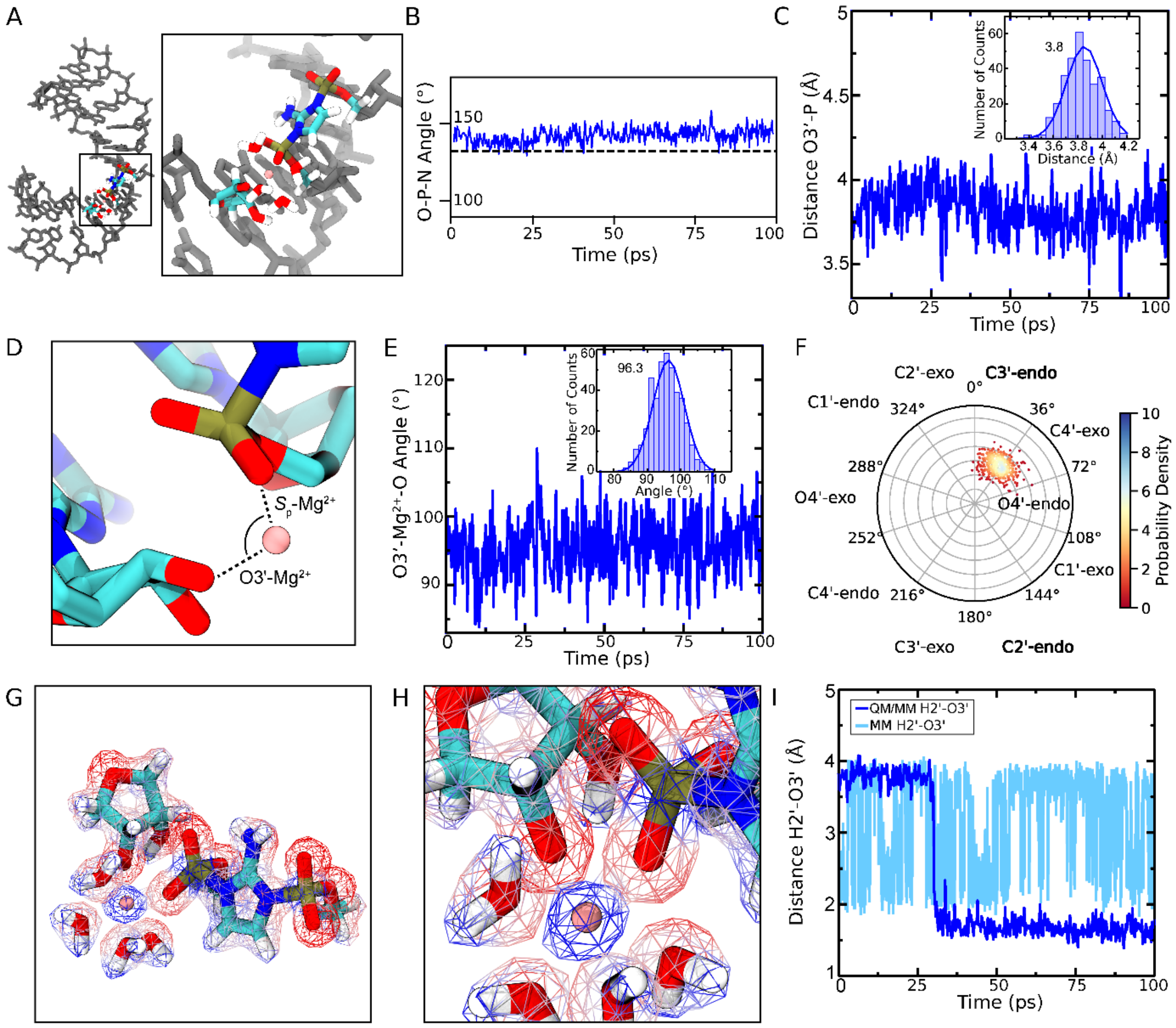

J

System	O3′-P Distance	Attack Angle	O3′-Mg^{2+}-O Angle	Primer Sugar Pucker
QM/MM	**3.8 Å**	**142.6°**	**96.3°**	**C3′-endo/C4′-exo**
Classical	**3.7 Å**	**148°**	**96°**	**C3′-endo**

***Figure S3*. QM/MM simulations of the $3'O^-$ w/ Mg^{2+}@S_P, 2-NH_2-Im:R_P system (Sim10).** (A) Molecular figure of the simulation system in licorice representation showing the portion of the duplex modeled in MM (grey) and the 49 atoms modeled using QM (colored by atom type). (B) The angle of attack during the QM/MM simulation, measured between O3′-P-N atoms, which agrees well with the CHARMM simulations (Figure 5B). (C) Time series of the O3′ (primer)-P (bridged dinucleotide) distance during the QM/MM simulation. The inset shows a probability distribution with the mean value indicated, obtained from a normal fit. This distance agrees well with the average distance from the CHARMM simulations (Figure 4D). (D) Molecular figure showing the O3′-Mg^{2+}-O(S_p) angle measured during the QM/MM simulation (E) Time course of the angle measured in (D). The inset shows a probability distribution with the mean value indicated, obtained from a normal fit. This angle agrees well with the average angle from the CHARMM simulations (Figure S16). (F) Circular histogram of pseudorotation angles of the terminal primer nucleotide sugar during the QM/MM simulation. The phase angles are based on the Altona-Sundaralingam[1] definition and are assigned to the puckering modes in multiples of 36°. (G) A molecular figure of the QM system with calculated SCF electron density colored by the electrostatic potential with positive charge in blue and negative charge in red. (H) A zoomed in view of (G), showing the polarization on the H2′ and O3′. (I) Time series of the H2′ and O3′ distance for the MM and QM/MM simulations, showing the tighter interaction in QM/MM due to polarization of the H2′. The time indicated corresponds to the QM/MM simulations, while the trace corresponding to the MM simulation goes to 3 ns. (J) Summary of measured parameters.

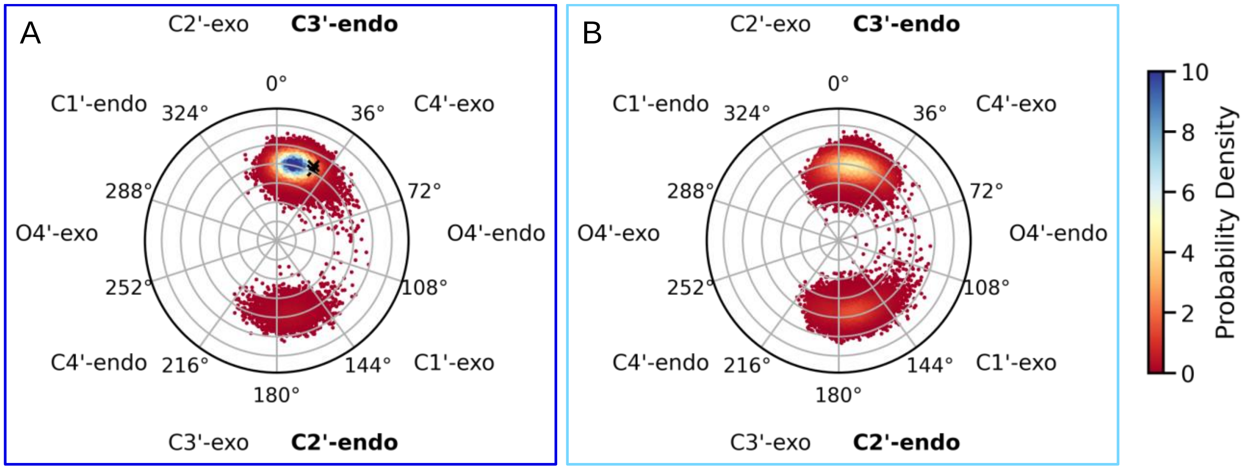

Figure S4. **Terminal primer sugar puckering in the absence of bound Mg^{2+} and a major groove-facing 2AI.** Circular histogram of pseudorotation angles and amplitudes of the terminal primer nucleotide sugar for the (A) 3′-OH w/o Mg^{2+} (Sim1) and (B) $3'O^-$ w/o Mg^{2+} (Sim2) simulation systems. The phase angles are based on the Altona-Sundaralingam[1] definition and are assigned to the puckering modes in multiples of 36°. Sugar pucker conformations of the primer nucleotide from the crystal structure, denoted with × markers in the circular plot show the sugars are in the C3′-endo conformation. Configurations that were more likely to be occupied are colored in blue, while less probable configurations are colored red.

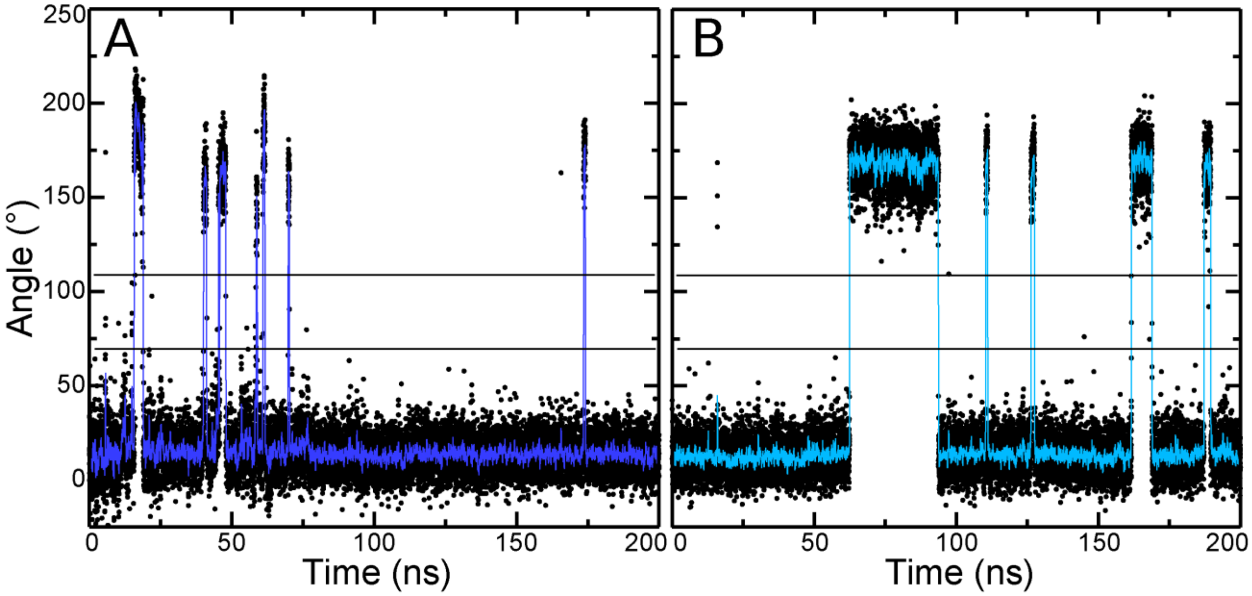

Figure S5. Time course of the terminal primer sugar pucker phase angle. (A) Phase angle of the terminal primer sugar for Sim1a. Individual data points shown as black circles, with a 0.15 ns running average. Horizontal lines show the range of angles corresponding to the O4'-endo conformation (72°-108°). (B) Phase angle of the terminal primer sugar for Sim2a. Individual data points shown as black circles, with a 0.15 ns running average. Horizontal lines show the range of angles corresponding to the O4'-endo conformation (72°-108°). For some transitions from C3'-endo to C2'-endo in both systems, data points between 72°-108° indicate a transition through the O4'-endo conformation.

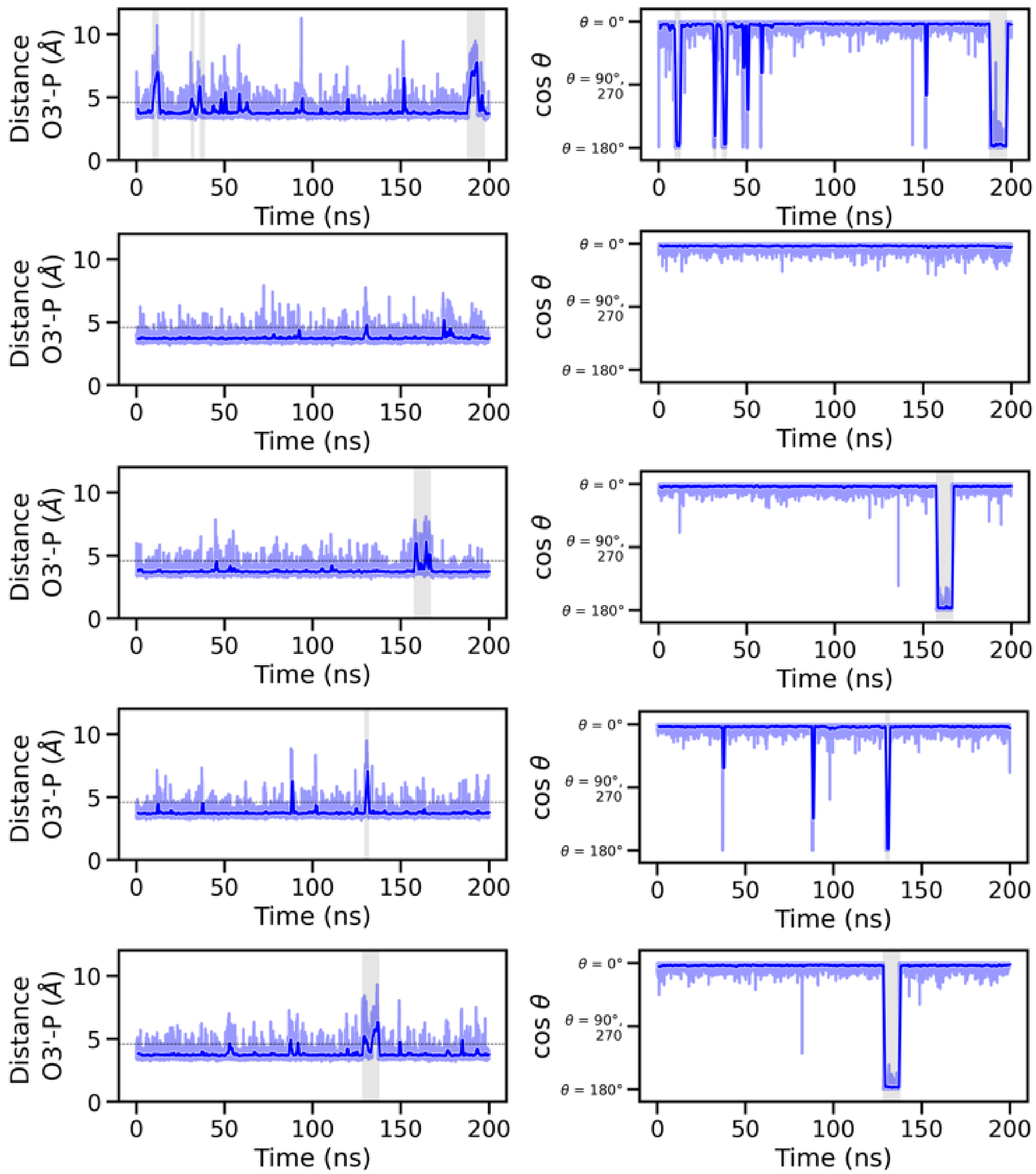

Figure S6. Time series of the distance O3′ (primer)-P (bridged dinucleotide) and primer nucleotide sugar pucker angle for the major groove-facing 3′-OH w/o Mg^{2+} simulation system (Sim1) for all five replicates. The horizontal dashed lines indicate the crystal structure value. Intervals with larger distance values and C2′-endo sugar conformations are highlighted in gray. For all time series plots, dark traces show the data averaged over a 1 ns window, while the lighter envelope shows the full range of the data recorded at 10 ps time steps in our simulations.

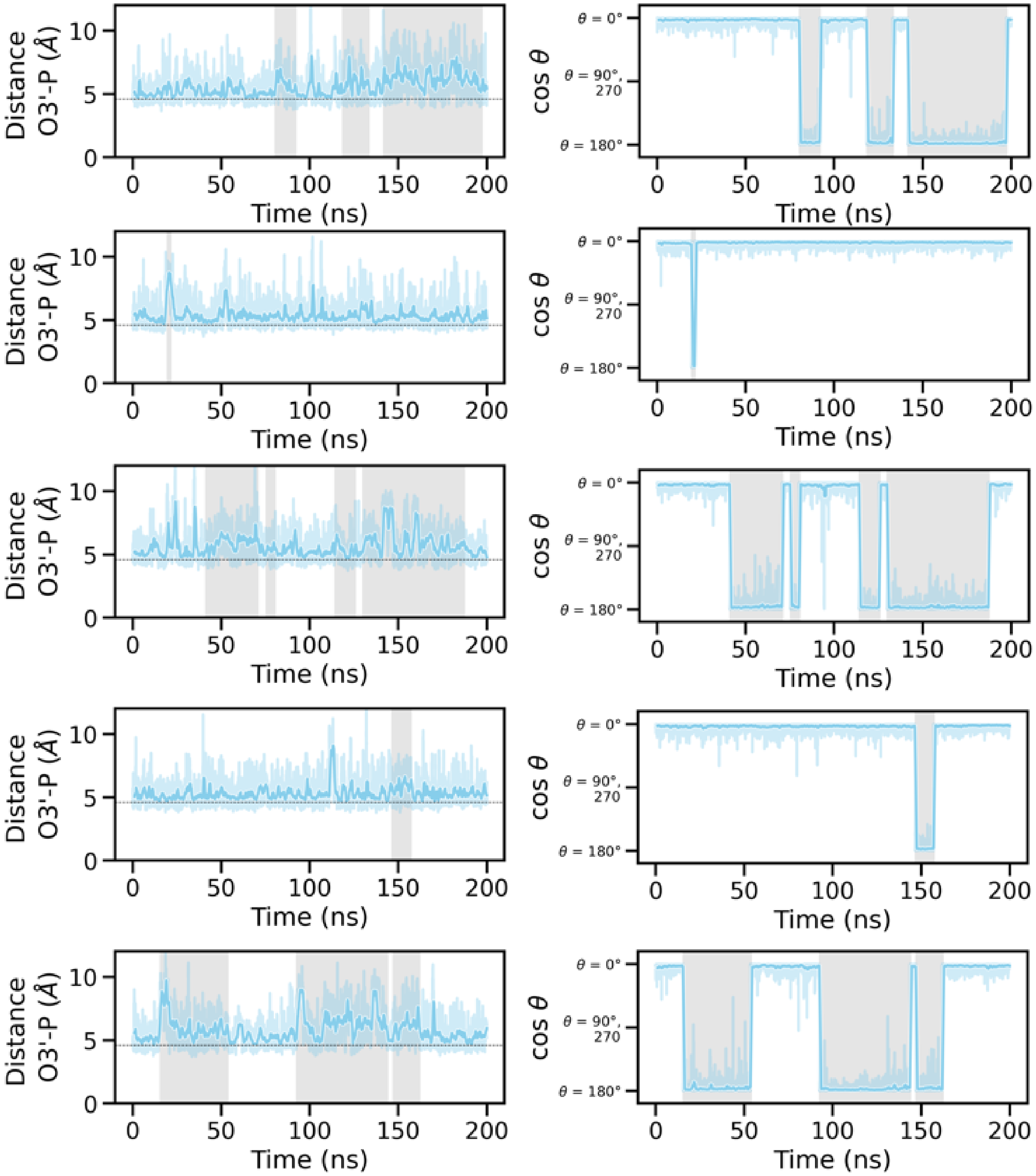

Figure S7. Time series of the distance O3′ (primer)-P (bridged dinucleotide) and primer nucleotide sugar pucker angle for the major groove-facing $3'O^{-}$ w/o Mg^{2+} simulation system (Sim2) for all five replicates. The horizontal dashed lines indicate the crystal structure value. Intervals with larger distance values and C2′-endo sugar conformations are highlighted in gray. For all time series plots, dark traces show the data averaged over a 1 ns window, while the lighter envelope shows the full range of the data recorded at 10 ps time steps in our simulations.

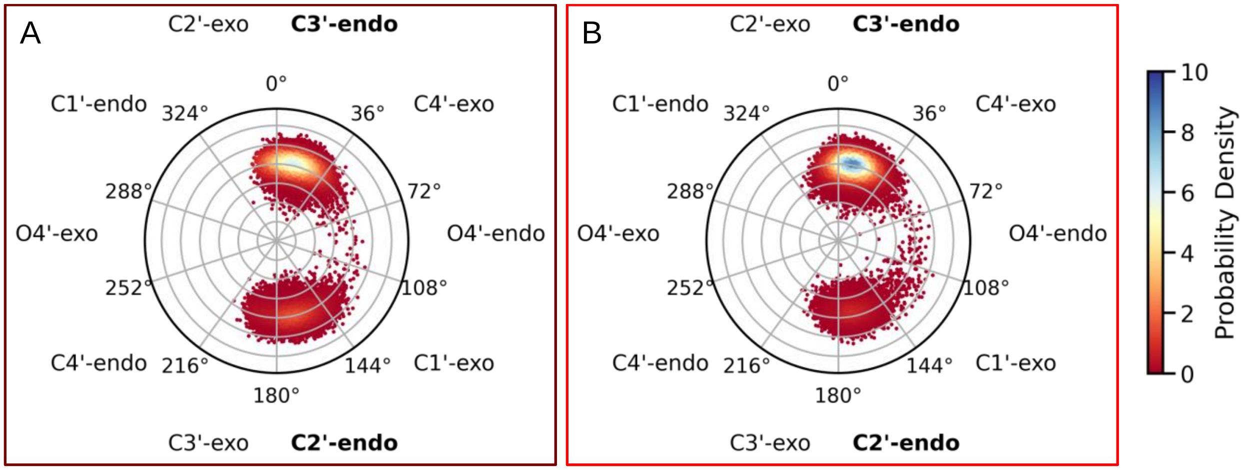

Figure S8. **Terminal primer sugar puckering in the absence of bound Mg^{2+} and a minor groove facing 2AI.** Circular histogram of pseudorotation angles of the terminal primer nucleotide sugar for the (A) 3′-OH w/o Mg^{2+} (Sim3) and (B) 3′O- w/o Mg^{2+} (Sim4) simulation systems where 2-NH_2-Im orientation is minor groove-facing. The phase angles are based on the Altona-Sundaralingam[1] definition and are assigned to the puckering modes in multiples of 36°. Configurations that were more likely to be occupied are colored in blue, while less probable configurations are colored red.

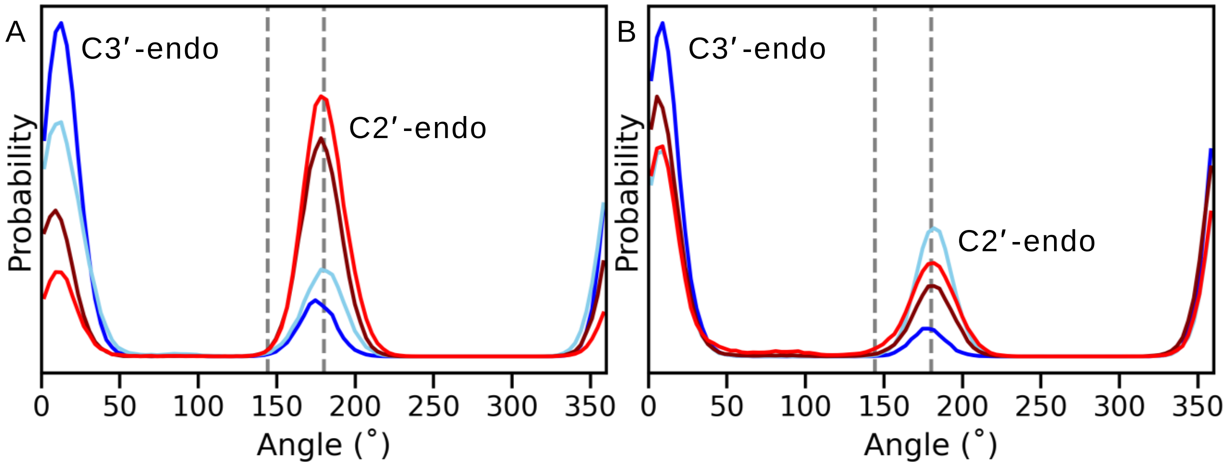

Figure S9. **Probability distribution of the pseudorotation angle of the bridged dinucleotide sugars in the absence of bound Mg^{2+}.** (A) G1 and (B) G2 positions in the major groove-facing conformations are shown for 3′-OH w/o Mg^{2+} (Sim1; dark blue) and 3′O- w/o Mg^{2+} (Sim2; light blue) and minor groove-facing conformations for 3′-OH w/o Mg^{2+} (Sim3; brown) and 3′O- w/o Mg^{2+} (Sim4; red). Dashed lines mark the region for C2′-endo sugar pucker.

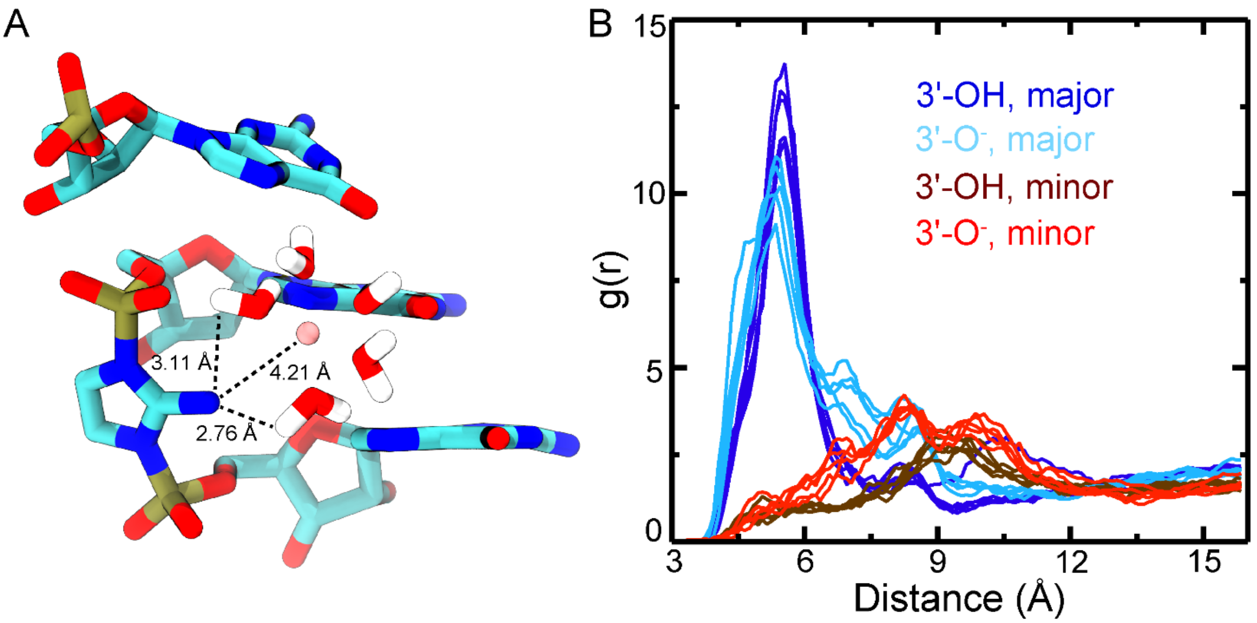

Figure S10. **Water mediated contacts between the bridging imidazolium and Mg^{2+}.** (A) Simulation snapshot (Sim1a) showing distances of Mg^{2+} ion and coordinating water molecules from the 2-NH_2-Im atom of the bridged nucleotide. (B) Radial distribution function of the distance between 2-NH_2-Im and Mg^{2+} ions in the MD simulation box for each simulation replicate.

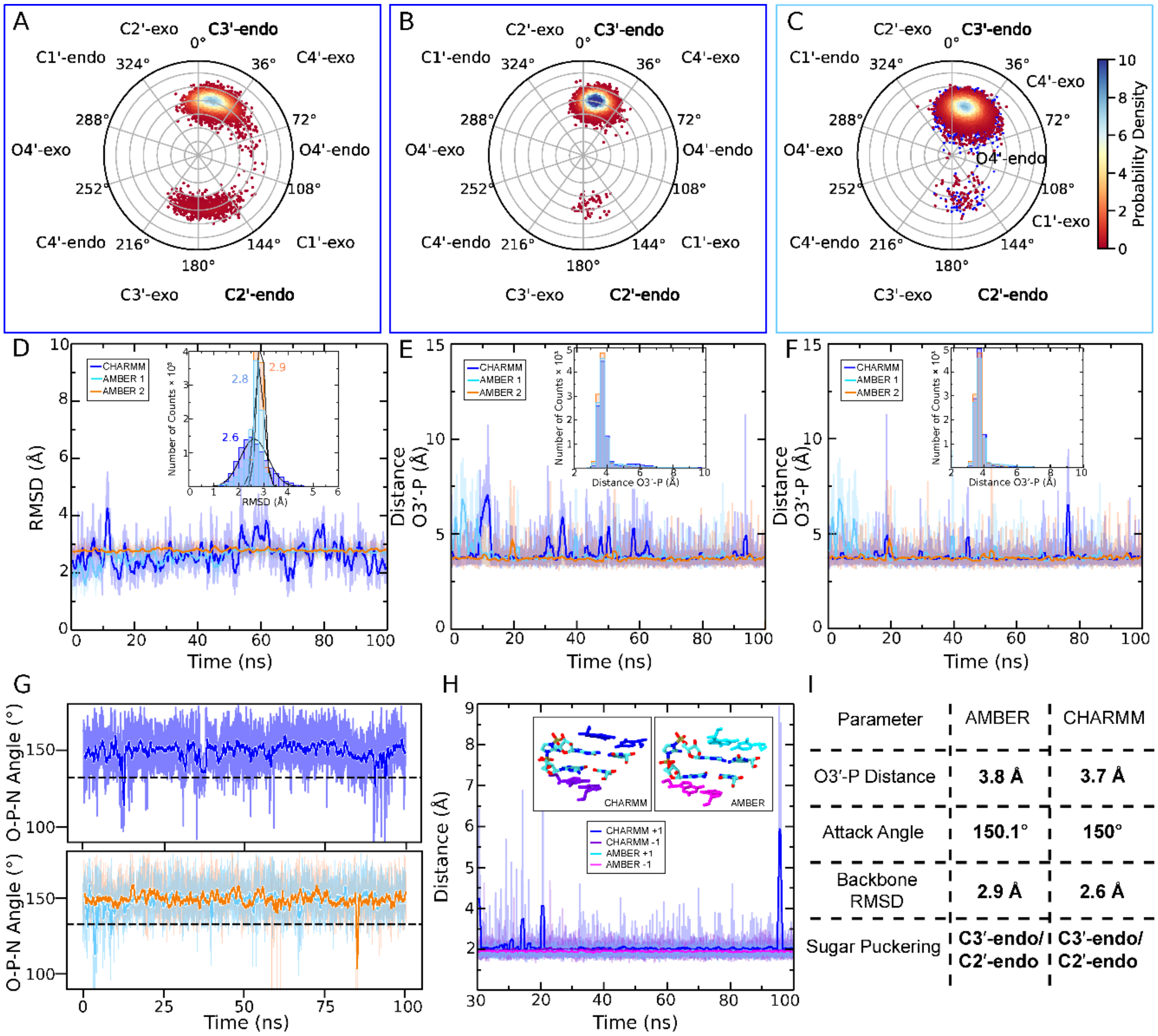

Figure S11. **Comparison of CHARMM (Sim1) and AMBER (Sim9) 3′-OH w/o Mg^{2+} simulations where 2-NH_2-Im orientation is major groove-facing.** Terminal primer sugar puckering for (A) 0-100 ns CHARMM, (B) 75-175 ns CHARMM, and (C) AMBER simulation systems. In (C), data points from the first 100 ns are shown on top, while data points from the second 100 ns are shown underneath in blue. The phase angles are based on the Altona-Sundaralingam[1] definition and are assigned to the puckering modes in multiples of 36°. (D) RMSD values (calculated for nucleic acid and bridged dinucleotide heavy atoms, see methods) of the 0-100 ns CHARMM and two 100 ns AMBER sub-trajectories. The inset shows a probability distribution for each, with mean values indicated in the corresponding color, obtained from a normal fit. Time series of the O3′ (primer)-P (bridged dinucleotide) distance for the 0-100 ns CHARMM and two 100 ns AMBER sub-trajectories (E), and the 75-175 ns CHARMM and two 100 ns AMBER sub-trajectories (F). The insets show a probability distribution for each. (G) The angle of attack during the 0-100 ns CHARMM (top) and two 100 ns AMBER (bottom) sub-trajectories, measured between O3′-P-N atoms. (H) Time series of the distances d(N1,N3) between the G-C base pairs flanking the bridged dinucleotide in the 0-100 ns CHARMM and AMBER simulations. The inset indicates which color traces correspond to the upstream and downstream positions for each simulation. (I) Summary of measured parameters comparing between the AMBER and CHARMM systems.

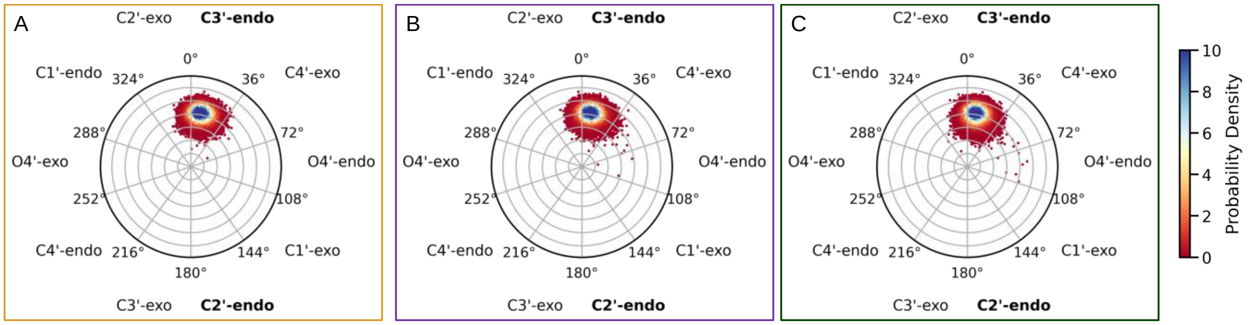

Figure S12. **Terminal primer sugar puckering with bound Mg^{2+}.** Circular histogram of pseudorotation angles of the terminal primer nucleotide sugar for the (A) 3′O⁻ w/ Mg^{2+}@S_P, 2-NH_2-Im:R_P (Sim5; yellow), (B) 3′O⁻ w/ Mg^{2+}@R_P, 2-NH_2-Im:R_P (Sim6; purple), and (C) 3′O⁻ w/ Mg^{2+}@R_P, 2-NH_2-Im:S_P (Sim7; green) simulation systems. The phase angles are based on the Altona-Sundaralingam[1] definition and are assigned to the puckering modes in multiples of 36°.

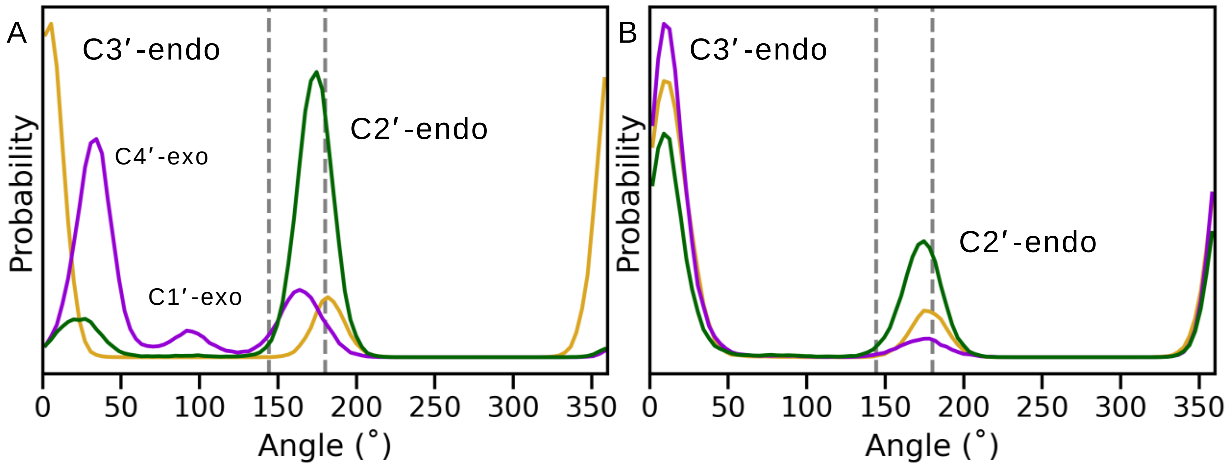

Figure S13. **Probability distribution of the pseudorotation angle of the bridged dinucleotide sugars with bound Mg^{2+}.** Probability distribution of the pseudorotation angle of the bridged dinucleotide sugars in the (A) G1 and (B) G2 positions for the simulation ensembles with Mg^{2+} in the reaction center: 3′O⁻ w/ Mg^{2+}@S_P, 2-NH_2-Im:R_P (Sim5; yellow), 3′O⁻ w/ Mg^{2+}@R_P, 2-NH_2-Im:R_P (Sim6; purple) and 3′O⁻ w/ Mg^{2+}@R_P, 2-NH_2-Im:S_P (Sim7; green). Dashed lines mark the region for C2′-endo sugar pucker.

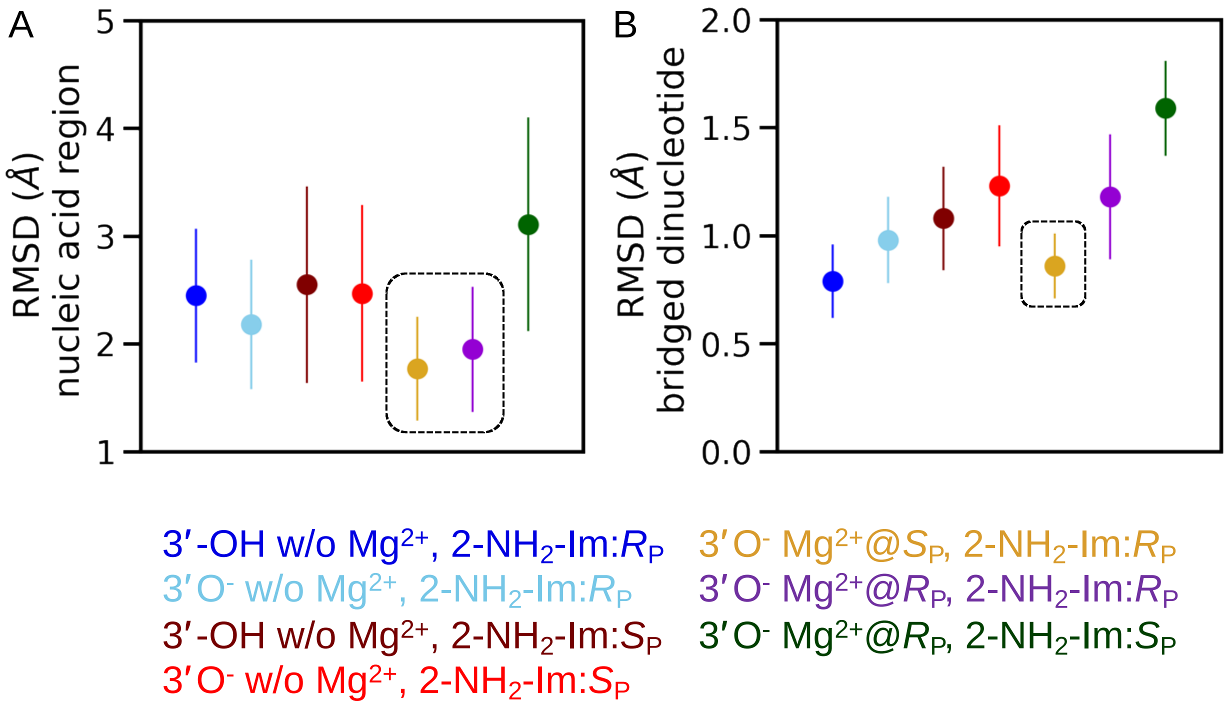

Figure S14. **A bridging Mg^{2+} in the reaction center stabilizes the primer extension complex.** Comparing mean ± standard deviation RMSD of (A) nucleic acid region and (B) bridged dinucleotide heavy atoms among the seven simulation ensembles discussed in this work. Systems highlighted in the main text are outlined.

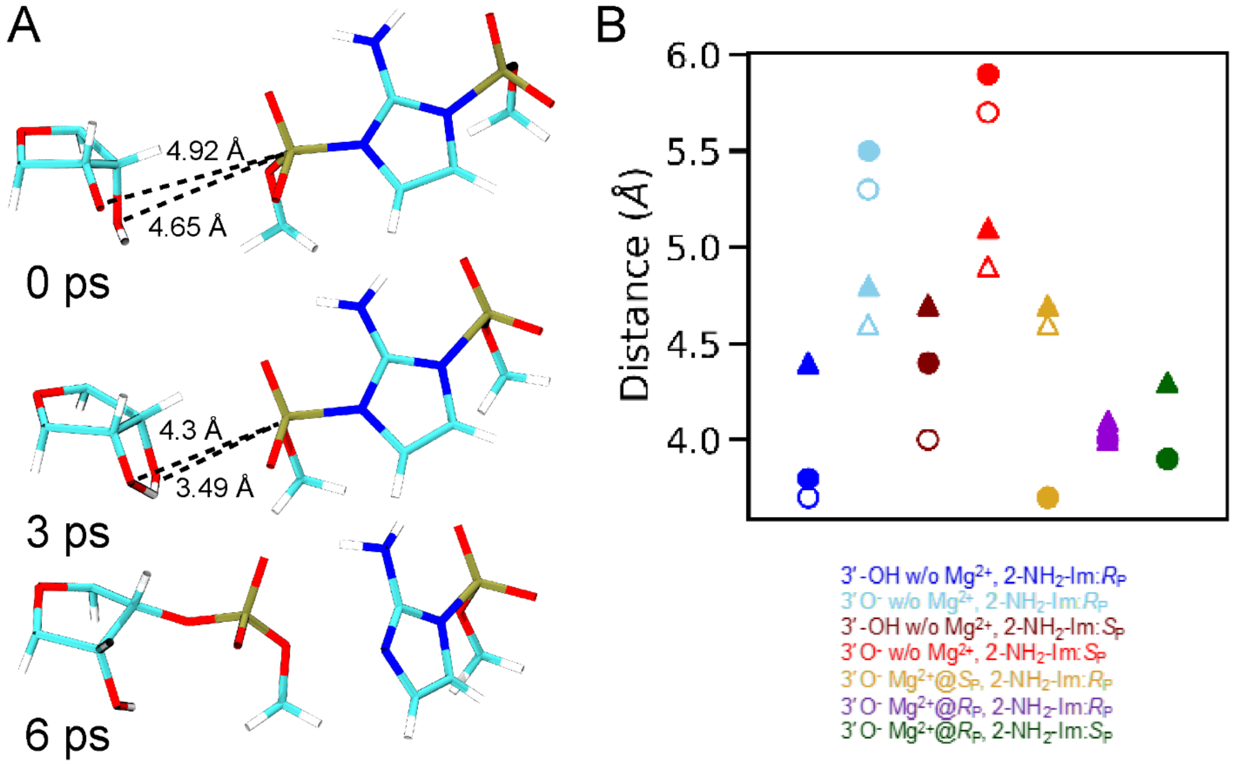

Figure S15. **Mg^{2+} increases the likelihood of forming the correct 3'-5' linkage.** (A) Reaction observed between the 2'-oxygen and reactive phosphate during Sim11. Quantum region shown in active bonds representation, which determines covalent bonds based on distance between atoms. (B) Distance between O2′-P (△ or ▲) and O3′-P (○ or ●) for the seven simulation ensembles discussed in this work. Open and closed markers show mean and median values respectively. Light blue and red highlighted simulation systems show a reversed trend where the average distance(O2′-P) < distance(O3′-P) whereas distance(O2′-P) ≈ distance(O3′-P) for the purple highlighted simulation system.

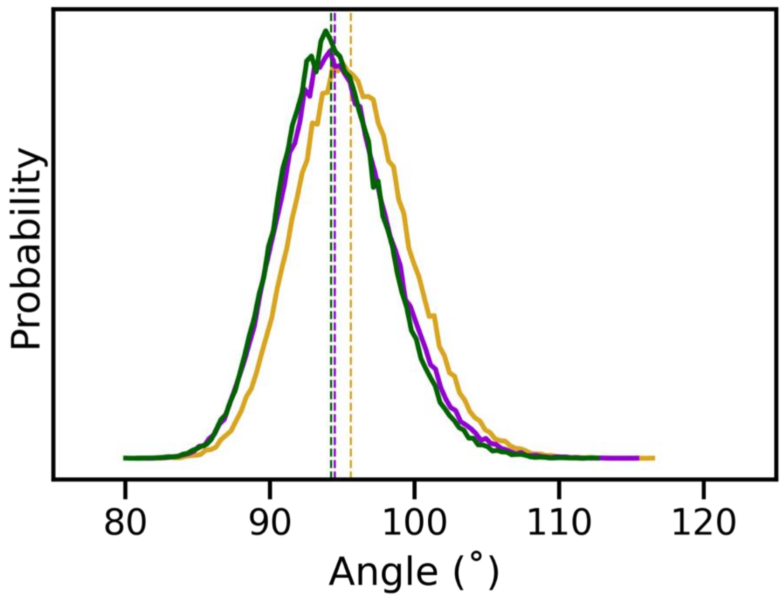

Figure S16. **Probability distribution of the Mg^{2+} coordination angle.** Angles measured between the O3′-Mg^{2+}-O (R_P or S_P) atoms in three simulation ensembles: 3′O⁻ w/ Mg^{2+}@S_P, 2-NH_2-Im:R_P (Sim5; yellow), 3′O⁻ w/ Mg^{2+}@R_P, 2-NH_2-Im:R_P (Sim6; purple) and 3′O⁻ w/ Mg^{2+}@R_P, 2-NH_2-Im:S_P (Sim7; green). Dashed lines show median values for the distributions.

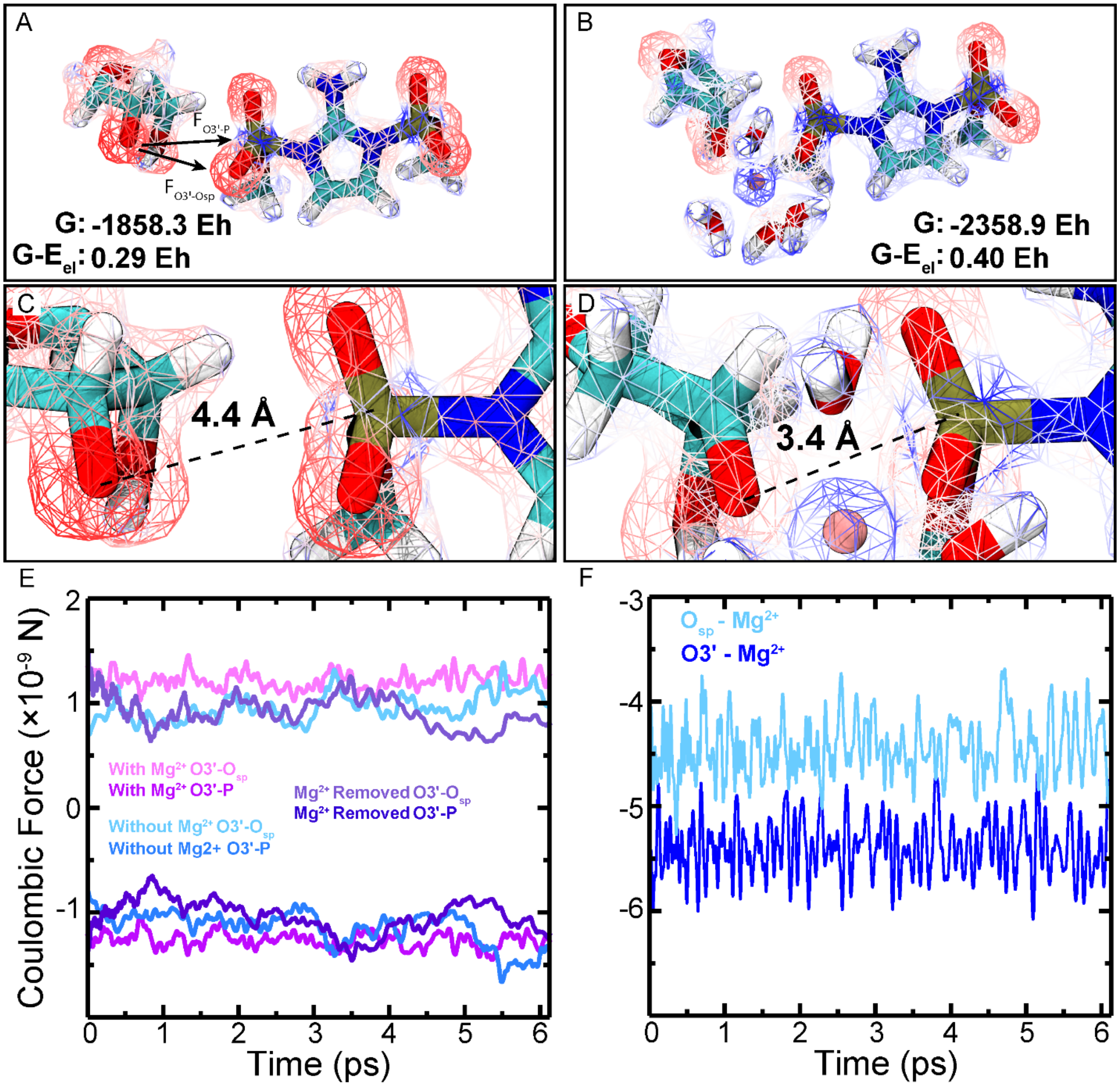

Figure S17. The presence of Mg^{2+} reduces electrostatic repulsion in the reaction center complex. A molecular figure of the QM system with calculated SCF electron density colored by the electrostatic potential with positive charge in blue and negative charge in red for the system without (A; Sim10) and with (B; Sim11) Mg^{2+} present, visualized at the same isovalue. Total Gibbs free energy (G) and Gibbs free energy minus electronic energy ($G\text{-}E_{el}$) are shown calculated using an HF-3c method with an implicit solvent model for each system. (C) Zoomed in view of (A), showing the O3'-P distance at the end of the simulation. (D) Zoomed in view of (B), showing the O3'-P distance at the end of the simulation, and a quantitatively reduced potential compared to (C) suggested by the lighter color of the electrostatic map. (E) Coulombic force calculated between the indicated atoms for the system without Mg^{2+} (Sim10), with Mg^{2+} (Sim11), and the system where Mg^{2+} was removed (Sim12). (F) Coulombic force calculated between the indicated atoms for the system with Mg^{2+} (Sim11), showing that in addition to the electrostatic influences demonstrated in (E), the presence of Mg^{2+} also exerts significant attractive forces between the relevant O atoms in the reactive center.

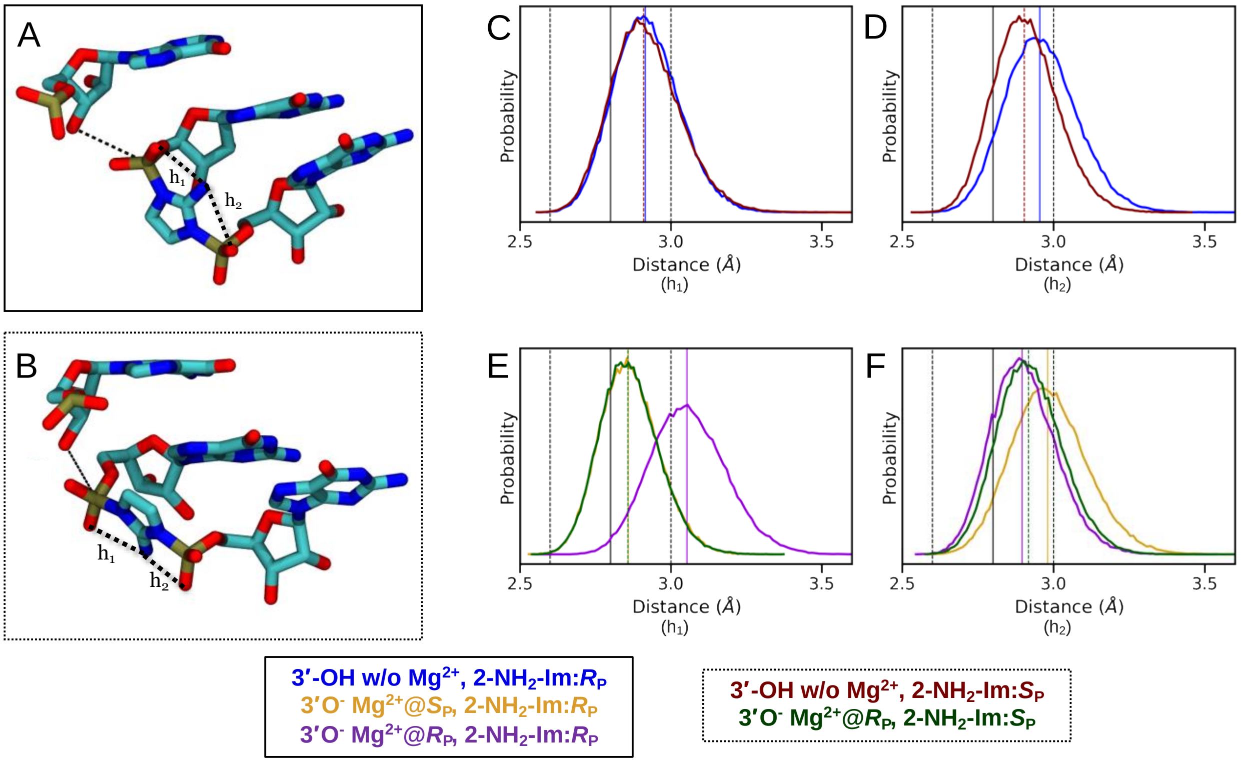

Figure S18. **Hydrogen bonds h_1 and h_2 between 2-NH_2-Im and non-bridging oxygens on the bridged dinucleotide suggest a preferred preorganization geometry.** (A) The major groove-facing and (B) minor groove-facing orientation of the bridged dinucleotide are shown. (C-F) Probability distribution of the hydrogen bond distances h_1 and h_2 for the various simulation systems. Median distance values from simulation with major and minor groove-facing orientations of bridged dinucleotide are shown in solid and dashed lines, respectively. Lines in black show hydrogen bond distances observed in the crystal structure.

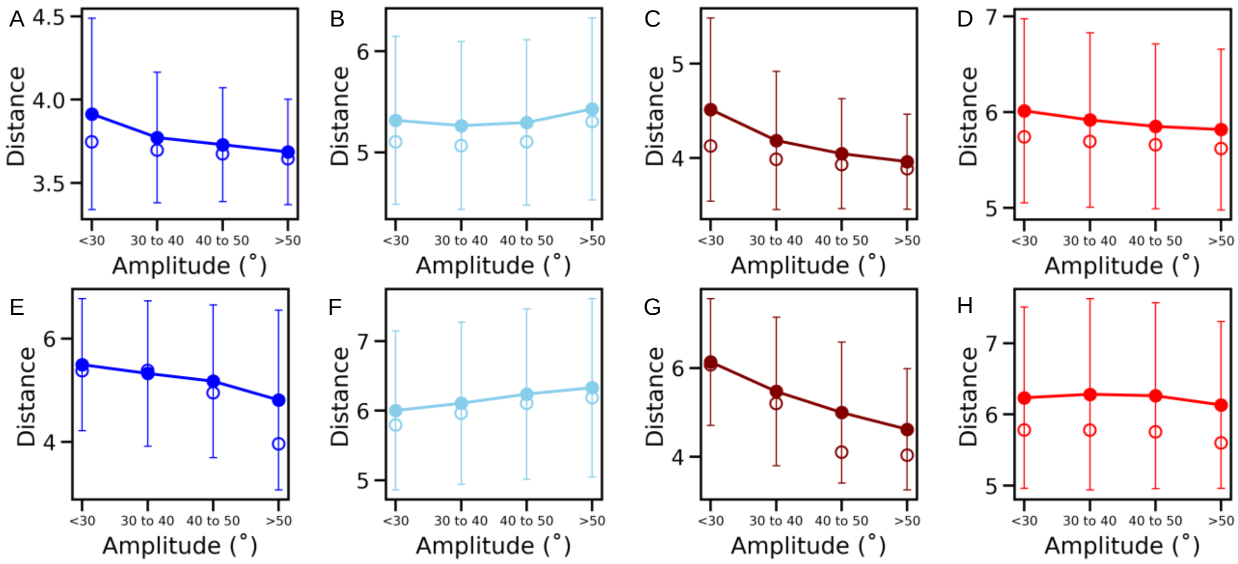

Figure S19. **O3′-P distance as a function of puckering amplitude without bound Mg^{2+}.** (A-D) Mean (●) and median (○) of the O3′-P distance distributions when the terminal primer sugar is in the C3′-endo conformation with amplitudes <30°, 30-40°, 40-50°, and >50°. (E-H) Mean (●) and median (○) of the O3′-P distance distributions when the terminal primer sugar is in the C2′-endo conformation with amplitudes <30°, 30-40°, 40-50°, and >50°. Simulation ensembles include major groove-facing 3′-OH (dark blue) and $3'O^-$ (light blue), and minor groove-facing 3′-OH (brown) and $3'O^-$ (red). Standard deviation is shown as colored bars.

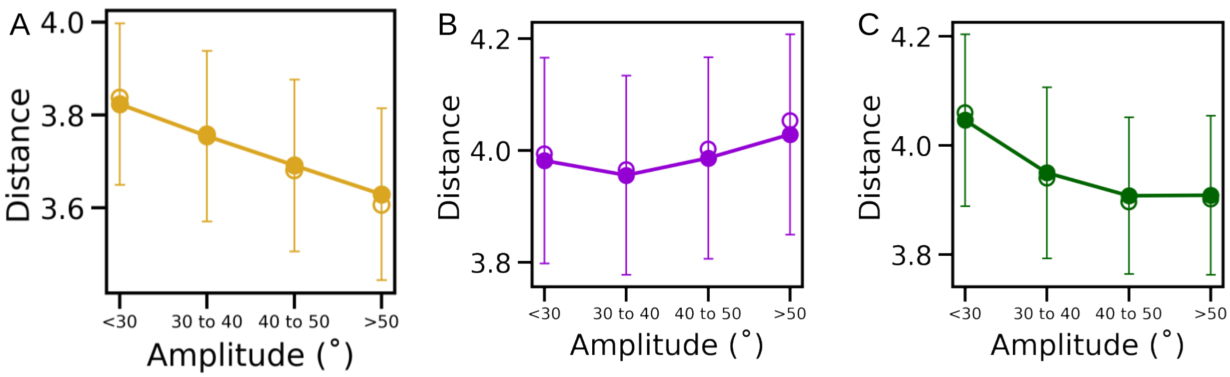

Figure S20. **O3′-P distance as a function of puckering amplitude with bound Mg^{2+}.** Mean (●) and median (○) of the O3′-P distance distributions when the terminal primer sugar is in the C3′-endo conformation with amplitudes <30°, 30-40°, 40-50°, and >50°. Simulation ensembles include (A) 3′O^- w/ Mg^{2+}@S_P, 2-NH_2-Im:R_P (Sim5; yellow), (B) 3′O^- w/ Mg^{2+}@R_P, 2-NH_2-Im:R_P (Sim6; purple) and (C) 3′O^- w/ Mg^{2+}@R_P, 2-NH_2-Im:S_P (Sim7; green). Simulation frames in C2′-endo are not observed. Standard deviation is shown as colored bars.

TABLE S1 2AI-Bridged Dinucleotide Parameters

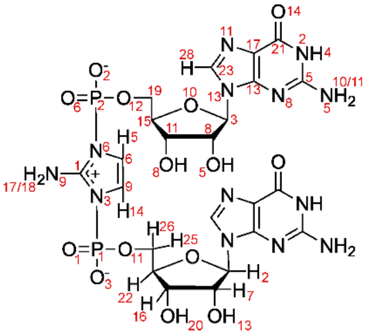

Atom Name	Atom Type	Charge
ATOM P1	PG1	1.599
ATOM P2	PG1	1.599
ATOM C1	CG2R53	0.483
ATOM C2	CG3C51	0.146
ATOM C3	CG3C51	0.146
ATOM N3	NG2R52	-0.557
ATOM O1	OG2P1	-0.821
ATOM O2	OG2P1	-0.821
ATOM C6	CG2R51	0.171
ATOM O6	OG2P1	-0.821
ATOM O12	OG303	-0.534
ATOM N6	NG2R52	-0.557
ATOM H5	HGR52	0.130
ATOM C1	CG2R53	0.483
ATOM N9	NG2P1	-0.621
ATOM H17	HGP2	0.329
ATOM H18	HGP2	0.329
ATOM O11	OG303	-0.534
ATOM O3	OG2P1	-0.821

Atom Name	Atom Type	Charge
ATOM C19	CG321	-0.101
ATOM C15	CG3C51	0.135
ATOM C11	CG3C51	0.145
ATOM O8	OG311	-0.650
ATOM C11	CG3C51	0.145
ATOM C8	CG3C51	0.143
ATOM C3	CG3C51	0.146
ATOM O10	OG3C51	-0.468
ATOM O8	OG311	-0.650
ATOM O5	OG311	-0.650
ATOM N13	NG2R51	-0.017
ATOM C23	CG2R53	0.251
ATOM N11	NG2R50	-0.595
ATOM C17	CG2RC0	0.004
ATOM C13	CG2RC0	0.264
ATOM N8	NG2R62	-0.738
ATOM C5	CG2R64	0.730
ATOM N5	NG2S3	-0.663
ATOM N2	NG2R61	-0.340

Atom Name	Atom Type	Charge
ATOM C21	CG2R63	0.544
ATOM O14	OG2D4	-0.509
ATOM H25	HGA2	0.090
ATOM H26	HGA2	0.090
ATOM H22	HGA1	0.090
ATOM H16	HGA1	0.090
ATOM H20	HGP1	0.419
ATOM H13	HGP1	0.419
ATOM H7	HGA1	0.090
ATOM H2	HGA1	0.090
ATOM H28	HGR52	0.143
ATOM H10	HGP4	0.327
ATOM H11	HGP4	0.327
ATOM H4	HGP1	0.267

Bond	Force Constant	Equilibrium Distance
CG2R51 CG2R51	410.00	1.3600
CG2R51 NG2R52	380.00	1.3700
CG2R51 HGR52	375.00	1.0830
CG2R53 NG2P1	300.00	1.3820
CG2R53 NG2R50	400.00	1.3200
CG2R53 NG2R51	320.00	1.3740
CG2R53 NG2R52	380.00	1.3200
CG2R53 HGR52	340.00	1.0900
CG2R63 CG2RC0	302.00	1.3600
CG2R63 NG2R61	340.00	1.3830
CG2R63 OG2D4	660.00	1.2340
CG2R64 NG2R61	400.00	1.3920
CG2R64 NG2R62	400.00	1.3420
CG2R64 NG2S3	360.00	1.3660
CG2RC0 CG2RC0	360.00	1.3850
CG2RC0 NG2R50	310.00	1.3650
CG2RC0 NG2R51	300.00	1.3750
CG2RC0 NG2R62	350.00	1.3150
CG321 CG3C51	222.50	1.5280
CG321 OG303	320.00	1.4400
CG321 HGA2	309.00	1.1110
CG3C51 CG3C51	195.00	1.5180
CG3C51 NG2R51	220.00	1.4580
CG3C51 OG311	428.00	1.4200
CG3C51 OG3C51	350.00	1.4250
CG3C51 HGA1	307.00	1.1000
NG2P1 HGP2	455.00	1.0000
NG2R52 PG1	180.00	1.7920
NG2R61 HGP1	474.00	1.0100
NG2S3 HGP4	488.00	1.0000
OG2P1 PG1	500.00	1.5100
OG303 PG1	190.00	1.6500
OG311 HGP1	545.00	0.9600

Angle	Force Constant	Equilibrium Angle
CG2R51 CG2R51 NG2R52	145.00	108.00
NG2P1 CG2R53 NG2R52	65.00	127.80
NG2R50 CG2R53 NG2R51	100.00	113.00
NG2R50 CG2R53 HGR52	39.00	124.80
NG2R51 CG2R53 HGR52	40.00	122.20
NG2R52 CG2R53 NG2R52	145.00	108.00
CG2RC0 CG2R63 NG2R61	70.00	107.80
CG2RC0 CG2R63 OG2D4	50.00	124.70
NG2R61 CG2R63 OG2D4	130.00	119.40
NG2R61 CG2R64 NG2R62	70.00	122.20
NG2R61 CG2R64 NG2S3	95.00	115.40
NG2R62 CG2R64 NG2S3	95.00	122.40
CG2R63 CG2RC0 CG2RC0	70.00	119.60
CG2R63 CG2RC0 NG2R50	125.00	129.00
CG2RC0 CG2RC0 NG2R50	100.00	110.00
CG2RC0 CG2RC0 NG2R51	100.00	105.70
CG2RC0 CG2RC0 NG2R62	60.00	127.40
NG2R51 CG2RC0 NG2R62	100.00	126.90
CG3C51 CG321 OG303	75.70	110.10
OG303 CG321 HGA2	60.00	110.10
CG321 CG3C51 OG3C51	45.00	111.50
CG3C51 CG3C51 NG2R51	110.00	111.00
CG3C51 CG3C51 OG311	75.70	110.10
CG3C51 CG3C51 OG3C51	45.00	111.10
CG3C51 CG3C51 HGA1	35.00	111.40
NG2R51 CG3C51 OG3C51	140.00	108.00
NG2R51 CG3C51 HGA1	43.00	111.00
OG311 CG3C51 HGA1	45.90	108.89
OG3C51 CG3C51 HGA1	70.00	107.30
CG2R53 NG2P1 HGP2	47.00	120.00
HGP2 NG2P1 HGP2	25.00	120.00
CG2R53 NG2R50 CG2RC0	120.00	103.80
CG2R53 NG2R51 CG2RC0	100.00	107.20

Angle	Force Constant	Equilibrium Angle
CG2R53 NG2R51 CG3C51	45.00	126.30
CG2RC0 NG2R51 CG3C51	45.00	126.50
CG2R51 NG2R52 CG2R53	145.00	108.00
CG2R63 NG2R61 CG2R64	70.00	131.10
CG2R63 NG2R61 HGP1	40.50	115.40
CG2R64 NG2R61 HGP1	45.00	115.60
CG2R64 NG2R62 CG2RC0	90.00	115.10
CG2R64 NG2S3 HGP4	40.00	121.50
HGP4 NG2S3 HGP4	31.00	117.00
CG3C51 OG311 HGP1	50.00	109.00
CG3C51 OG3C51 CG3C51	95.00	111.00
NG2R52 PG1 OG2P1	140.00	110.60
NG2R52 PG1 OG303	60.00	103.20
OG2P1 PG1 OG2P1	104.00	120.00
OG2P1 PG1 OG303	98.90	107.50

Angle	Force Cons.	Eq. Angle	K_{UB}	S_0
CG2R51 CG2R51 HGR52	22.00	130.00	15.00	2.2150
NG2R52 CG2R51 HGR52	22.00	122.00	15.00	2.1800
CG3C51 CG321 HGA2	34.60	110.10	22.53	2.1790
HGA2 CG321 HGA2	35.50	109.00	5.40	1.8020
CG321 CG3C51 CG3C51	58.00	115.00	8.00	2.5610
CG321 CG3C51 HGA1	34.60	110.10	22.53	2.1790
CG3C51 CG3C51 CG3C51	58.00	109.50	11.16	2.5610
CG2R51 NG2R52 PG1	44.00	126.30	23.00	2.4300
CG2R53 NG2R52 PG1	44.00	126.30	23.00	2.4300
CG321 OG303 PG1	20.00	120.00	35.00	2.3300

Improper			
CG2R53 NG2R52 NG2R52 NG2P1	90.0000	0	0.00
CG2R63 CG2RC0 NG2R61 OG2D4	90.0000	0	0.00
CG2R64 NG2R61 NG2R62 NG2S3	40.0000	0	0.00
NG2S3 HGP4 HGP4 CG2R64	9.0000	0	0.00

Dihedral	Force Cons.	Mult.	Phase
NG2R52 CG2R51 CG2R51 NG2R52	12.0000	2	180.00
NG2R52 CG2R51 CG2R51 HGR52	2.5000	2	180.00
HGR52 CG2R51 CG2R51 HGR52	1.0000	2	180.00
CG2R51 CG2R51 NG2R52 CG2R53	12.0000	2	180.00
CG2R51 CG2R51 NG2R52 PG1	6.0000	2	180.00
HGR52 CG2R51 NG2R52 CG2R53	2.5000	2	180.00
HGR52 CG2R51 NG2R52 PG1	9.0000	2	180.00
NG2R52 CG2R53 NG2P1 HGP2	2.4000	2	180.00
NG2R51 CG2R53 NG2R50 CG2RC0	14.0000	2	180.00
HGR52 CG2R53 NG2R50 CG2RC0	5.2000	2	180.00
NG2R50 CG2R53 NG2R51 CG2RC0	6.0000	2	180.00
NG2R50 CG2R53 NG2R51 CG3C51	11.0000	2	180.00
HGR52 CG2R53 NG2R51 CG2RC0	5.6000	2	180.00
HGR52 CG2R53 NG2R51 CG3C51	0.0000	2	180.00
NG2P1 CG2R53 NG2R52 CG2R51	12.0000	2	180.00
NG2P1 CG2R53 NG2R52 PG1	3.7000	2	180.00
NG2R52 CG2R53 NG2R52 CG2R51	12.0000	2	180.00
NG2R52 CG2R53 NG2R52 PG1	3.7000	2	180.00
NG2R61 CG2R63 CG2RC0 CG2RC0	0.2000	2	180.00
NG2R61 CG2R63 CG2RC0 NG2R50	2.0000	2	180.00
OG2D4 CG2R63 CG2RC0 CG2RC0	14.0000	2	180.00
OG2D4 CG2R63 CG2RC0 NG2R50	0.0000	2	180.00
CG2RC0 CG2R63 NG2R61 CG2R64	0.2000	2	180.00
CG2RC0 CG2R63 NG2R61 HGP1	3.6000	2	180.00
OG2D4 CG2R63 NG2R61 CG2R64	14.0000	2	180.00
OG2D4 CG2R63 NG2R61 HGP1	0.0000	2	180.00
NG2R62 CG2R64 NG2R61 CG2R63	0.2000	2	180.00
NG2R62 CG2R64 NG2R61 HGP1	3.6000	2	180.00
NG2S3 CG2R64 NG2R61 CG2R63	4.0000	2	180.00
NG2S3 CG2R64 NG2R61 HGP1	0.0000	2	180.00
NG2R61 CG2R64 NG2R62 CG2RC0	2.0000	2	180.00
NG2S3 CG2R64 NG2R62 CG2RC0	4.0000	2	180.00
NG2R61 CG2R64 NG2S3 HGP4	1.2000	2	180.00
NG2R62 CG2R64 NG2S3 HGP4	1.0000	2	180.00
CG2R63 CG2RC0 CG2RC0 NG2R51	10.0000	2	180.00

Dihedral	Force Cons.	Mult.	Phase
CG2R63 CG2RC0 CG2RC0 NG2R62	2.0000	2	180.00
NG2R50 CG2RC0 CG2RC0 NG2R51	10.0000	2	180.00
NG2R50 CG2RC0 CG2RC0 NG2R62	7.0000	2	180.00
CG2R63 CG2RC0 NG2R50 CG2R53	2.0000	2	180.00
CG2RC0 CG2RC0 NG2R50 CG2R53	6.0000	2	180.00
CG2RC0 CG2RC0 NG2R51 CG2R53	6.0000	2	180.00
CG2RC0 CG2RC0 NG2R51 CG3C51	11.0000	2	180.00
NG2R62 CG2RC0 NG2R51 CG2R53	2.0000	2	180.00
NG2R62 CG2RC0 NG2R51 CG3C51	11.0000	2	180.00
CG2RC0 CG2RC0 NG2R62 CG2R64	0.2000	2	180.00
NG2R51 CG2RC0 NG2R62 CG2R64	2.0000	2	180.00
OG303 CG321 CG3C51 CG3C51	2.5000	1	180.00
OG303 CG321 CG3C51 CG3C51	0.4000	2	0.00
OG303 CG321 CG3C51 CG3C51	0.8000	3	180.00
OG303 CG321 CG3C51 CG3C51	0.2000	4	180.00
OG303 CG321 CG3C51 OG3C51	3.4000	1	180.00
OG303 CG321 CG3C51 HGA1	0.1950	3	0.00
HGA2 CG321 CG3C51 CG3C51	0.1600	3	0.00
HGA2 CG321 CG3C51 OG3C51	0.1600	3	0.00
HGA2 CG321 CG3C51 HGA1	0.1600	3	0.00
CG3C51 CG321 OG303 PG1	0.6000	1	180.00
CG3C51 CG321 OG303 PG1	0.6500	2	0.00
CG3C51 CG321 OG303 PG1	0.0500	3	0.00
HGA2 CG321 OG303 PG1	0.0000	3	0.00
CG321 CG3C51 CG3C51 CG3C51	0.1900	3	0.00
CG321 CG3C51 CG3C51 OG311	0.1400	3	0.00
CG321 CG3C51 CG3C51 HGA1	0.1900	3	0.00
CG3C51 CG3C51 CG3C51 CG3C51	0.4100	3	180.00
CG3C51 CG3C51 CG3C51 NG2R51	0.0000	3	0.00
CG3C51 CG3C51 CG3C51 OG311	0.2000	3	0.00
CG3C51 CG3C51 CG3C51 OG3C51	0.0000	3	0.00
CG3C51 CG3C51 CG3C51 HGA1	0.1900	3	0.00
NG2R51 CG3C51 CG3C51 OG311	0.0000	3	0.00
NG2R51 CG3C51 CG3C51 HGA1	0.0000	3	0.00
OG311 CG3C51 CG3C51 OG311	0.0000	3	0.00

Dihedral	Force Cons.	Mult.	Phase
OG311 CG3C51 CG3C51 OG3C51	0.2000	3	0.00
OG311 CG3C51 CG3C51 OG3C51	0.6000	4	180.0
OG311 CG3C51 CG3C51 OG3C51	0.3000	5	0.00
OG311 CG3C51 CG3C51 OG3C51	0.5000	6	0.00
OG311 CG3C51 CG3C51 HGA1	0.1950	3	0.00
OG3C51 CG3C51 CG3C51 HGA1	0.1900	3	0.00
HGA1 CG3C51 CG3C51 HGA1	0.1900	3	0.00
CG3C51 CG3C51 NG2R51 CG2R53	0.0000	3	180.0
CG3C51 CG3C51 NG2R51 CG2RC0	0.0000	3	0.00
OG3C51 CG3C51 NG2R51 CG2R53	1.1000	1	0.00
OG3C51 CG3C51 NG2R51 CG2RC0	1.1000	1	180.0
OG3C51 CG3C51 NG2R51 CG2RC0	0.2000	3	0.00
HGA1 CG3C51 NG2R51 CG2R53	0.2500	2	180.0
HGA1 CG3C51 NG2R51 CG2R53	0.1950	3	0.00
HGA1 CG3C51 NG2R51 CG2RC0	0.2500	2	180.0
CG3C51 CG3C51 OG311 HGP1	0.2900	1	0.00
CG3C51 CG3C51 OG311 HGP1	0.6200	2	0.00
CG3C51 CG3C51 OG311 HGP1	0.0500	3	0.00
HGA1 CG3C51 OG311 HGP1	0.1800	3	0.00
CG321 CG3C51 OG3C51 CG3C51	0.3000	3	0.00
CG3C51 CG3C51 OG3C51 CG3C51	0.5000	3	0.00
NG2R51 CG3C51 OG3C51 CG3C51	0.0000	3	0.00
HGA1 CG3C51 OG3C51 CG3C51	0.3000	3	0.00
CG2R51 NG2R52 PG1 OG2P1	0.4000	3	0.00
CG2R51 NG2R52 PG1 OG2P1	0.5000	4	0.00
CG2R51 NG2R52 PG1 OG303	1.9000	2	0.00
CG2R53 NG2R52 PG1 OG2P1	0.4000	3	0.00
CG2R53 NG2R52 PG1 OG2P1	0.5000	4	0.00
CG2R53 NG2R52 PG1 OG303	1.9000	2	0.00
CG321 OG303 PG1 NG2R52	0.4000	1	0.00
CG321 OG303 PG1 NG2R52	0.2600	2	0.00
CG321 OG303 PG1 NG2R52	0.3500	3	0.00
CG321 OG303 PG1 OG2P1	0.1000	3	0.00

TABLE S2 Overview of MD Simulations

System	Label	Simulation time	Simulation Type	Size (# atoms)	System Size (nm^3)	Number of Ions (Cl/Mg^{2+})	Force Field/Method
**3′-OH w/o Mg^{2+} Major Groove**	Sim1a-e	1,005 ns	EQ	34,958	7.2 × 7.2 × 7.2	64/42	CHARMM
**3′O^- w/o Mg^{2+} Major Groove**	Sim2a-e	1,005 ns	EQ	34,957	7.2 × 7.2 × 7.2	64/42	CHARMM
**3′-OH w/o Mg^{2+} Minor Groove**	Sim3a-e	1,005 ns	EQ	34,958	7.2 × 7.2 × 7.2	64/42	CHARMM
**3′O^- w/o Mg^{2+} Minor Groove**	Sim4a-e	1,005 ns	EQ	34,957	7.2 × 7.2 × 7.2	64/42	CHARMM
**3′O^- Mg^{2+}@S_P, 2-NH_2-Im:R_P**	Sim5a-e	1,005 ns	EQ	35,025	6.8 × 7.1 × 7.3	65/43	CHARMM
**3′O^- Mg^{2+}@R_P, 2-NH_2-Im:R_P**	Sim6a-e	1,005 ns	EQ	35,025	6.8 × 7.1 × 7.3	65/43	CHARMM
**3′O^- Mg^{2+}@R_P, 2-NH_2-Im:S_P**	Sim7a-e	1,005 ns	EQ	35,025	6.8 × 7.1 × 7.3	65/43	CHARMM
**3′O^- Mg^{2+}@S_P, 2-NH_2-Im:S_P**	Sim8a-e	1,005 ns	EQ	35,025	6.8 × 7.1 × 7.3	65/43	CHARMM
**3′-OH w/o Mg^{2+} Major Groove**	Sim9	203 ns	EQ	34,958	7.2 × 7.2 × 7.2	64/42	AMBER
**3′O^- Mg^{2+}@S_P, 2-NH_2-Im:R_P**	Sim10	101 ps	QM/MM	35,025	6.8 × 7.1 × 7.3	65/43	CHARMM/HF-3c
**3′O^- w/o Mg^{2+} Major Groove**	Sim11	10 ps	QM/MM	35,023	6.8 × 7.1 × 7.3	63/42	CHARMM/HF-3c
**3′O^- Mg^{2+} Removed Major Groove**	Sim12	19 ps	QM/MM	35,023	6.8 × 7.1 × 7.3	63/42	CHARMM/HF-3c

SUPPORTING REFERENCES

(1) Altona, C.; Sundaralingam, M. Conformational Analysis of the Sugar Ring in Nucleosides and Nucleotides. New Description Using the Concept of Pseudorotation. *J. Am. Chem. Soc.* **1972**, *94* (23), 8205–8212. https://doi.org/10.1021/ja00778a043.
