# Supplementary material for: Simulations predict preferred Mg2+ coordination in a nonenzymatic primer-extension reaction center
Source: Biophys J. 2024 May 3;123(12):1579–91. doi: 10.1016/j.bpj.2024.04.032 (PMC11214020; doi:10.1016/j.bpj.2024.04.032)
Supplement: Document S2. Article plus supporting material [file mmc2.pdf]

Biophysical Journal
Article

Biophysical Society

Simulations predict preferred Mg^{2+} coordination in a nonenzymatic primer-extension reaction center

Shriyaa Mittal,[1,2] Collin Nisler,[4] and Jack W. Szostak[1,2,3,4,*]
[1]Howard Hughes Medical Institute, Department of Molecular Biology, Center for Computational and Integrative Biology, Massachusetts General Hospital, Boston, Massachusetts; [2]Department of Genetics, Harvard Medical School, Boston, Massachusetts; [3]Department of Chemistry and Chemical Biology, Harvard University, Cambridge, Massachusetts; and [4]Howard Hughes Medical Institute, Department of Chemistry, University of Chicago, Chicago, Illinois

ABSTRACT The mechanism by which genetic information was copied prior to the evolution of ribozymes is of great interest because of its importance to the origin of life. The most effective known process for the nonenzymatic copying of an RNA template is primer extension by a two-step pathway in which 2-aminoimidazole-activated nucleotides first react with each other to form an imidazolium-bridged intermediate that subsequently reacts with the primer. Reaction kinetics, structure-activity relationships, and X-ray crystallography have provided insight into the overall reaction mechanism, but many puzzles remain. In particular, high concentrations of Mg^{2+} are required for efficient primer extension, but the mechanism by which Mg^{2+} accelerates primer extension remains unknown. By analogy with the mechanism of DNA and RNA polymerases, a role for Mg^{2+} in facilitating the deprotonation of the primer 3′-hydroxyl is often assumed, but no catalytic metal ion is seen in crystal structures of the primer-extension complex. To explore the potential effects of Mg^{2+} binding in the reaction center, we performed atomistic molecular dynamics simulations of a series of modeled complexes in which a Mg^{2+} ion was placed in the reaction center with inner-sphere coordination with different sets of functional groups. Our simulations suggest that coordination of a Mg^{2+} ion with both O3′ of the terminal primer nucleotide and the pro-S_p nonbridging oxygen of the reactive phosphate of an imidazolium-bridged dinucleotide would help to pre-organize the structure of the primer/template substrate complex to favor the primer-extension reaction. Our results suggest that the catalytic metal ion may play an important role in overcoming electrostatic repulsion between a deprotonated O3′ and the reactive phosphate of the bridged dinucleotide and lead to testable predictions of the mode of Mg^{2+} binding that is most relevant to catalysis of primer extension.

SIGNIFICANCE Prior to the evolution of complex enzymes, the replication of genetic material must have relied on nonenzymatic mechanisms. Nonenzymatic RNA template copying can be achieved through the extension of a primer by reaction with a 2-aminoimidazole (2AI) bridged dinucleotide in the presence of Mg^{2+}. Despite progress in understanding the mechanism of this reaction, the catalytic role of Mg^{2+} remains poorly understood. Here, we present a series of molecular dynamics simulations of a model RNA primer-extension complex in different potential reactive conformations. We find that one configuration of both the 2AI moiety and coordination state of the Mg^{2+} promotes a geometry that is most favorable to reaction, suggesting a potential structural role for Mg^{2+} and providing insights to guide future experiments.

INTRODUCTION

The idea that nonenzymatic RNA replication preceded the evolution of macromolecular catalysis of replication has a long history, going back to the early experimental work of Orgel et al. (1–3). Much subsequent work has focused on a search for optimal activation chemistry that would enable activated nucleotides to polymerize spontaneously on a template strand. The Orgel lab first demonstrated the template-directed polymerization of nucleoside 5′-phosphorimidazolides in 1968 (4), then in 1982 showed that 2-methylimidazole was a superior activating group that enabled faster and more extensive copying of G/C-rich templates (5). Many other potential activating groups have since been examined. The Richert group has studied the templated polymerization of nucleotides activated by such leaving groups as N-methyl imidazole and oxyazabenzotriazole (6,7). Our laboratory identified 2-aminoimidazole (2AI) as a nucleotide-activating moiety that enabled extensive copying of templates containing all

Submitted December 28, 2023, and accepted for publication April 30, 2024.

*Correspondence: jwszostak@uchicago.edu

Shriyaa Mittal and Collin Nisler contributed equally to this work.

Editor: Lars Nordenskiöld.
https://doi.org/10.1016/j.bpj.2024.04.032

© 2024 Biophysical Society.
This is an open access article under the CC BY license (http://creativecommons.org/licenses/by/4.0/).

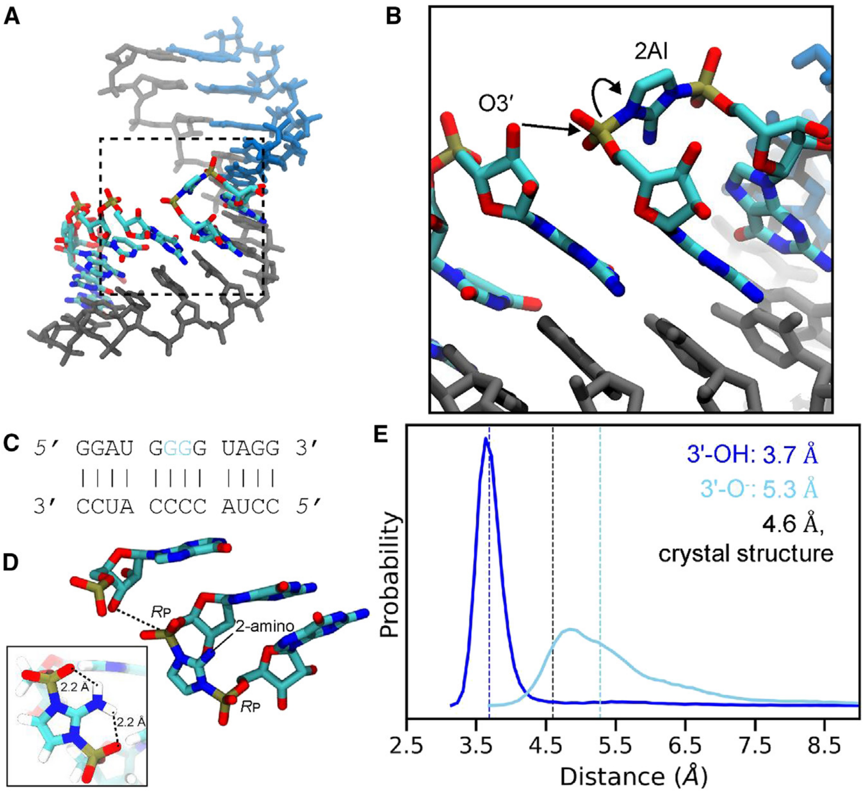

FIGURE 1 Overview of simulation system and reaction-center dynamics without Mg^{2+}. (*A*) The template is shown in gray and helper oligo in blue, and reacting imidazolium-bridged dinucleotide and primer are colored by atom type. Hydrogen atoms are not shown for clarity. (*B*) A zoomed in view of (*A*), showing the attack of the template 3′-hydroxyl on the phosphate of the dinucleotide and subsequent displacement of the activated monomer. (*C*) Schematic of the RNA complex used in this work. Five-nucleotide-long primer and helper oligonucleotides sandwich the G*G bridged dinucleotide (shown in blue) and basepair with the 12-nt template. (*D*) Structure of the primer-extension reaction site, showing the last primer nucleotide and the G*G bridged dinucleotide. The dashed line shows the distance between reacting O3′ and P atoms, and R_p atoms of the backbone phosphates are indicated. The inset shows the 2AI-R_p oxygen hydrogen bonds. (*E*) Probability distribution of the distance between O3′ and P atoms observed in the 3′-hydroxyl without Mg^{2+} (Sim1; dark blue) and 3′O^- without Mg^{2+} (Sim2; light blue). Dashed lines show median values for the distance distributions. The black dashed line shows the distance value of 4.6 Å in the crystal structure (20).

four nucleotides. Furthermore, we and others have found prebiotically reasonable pathways for the synthesis of 2AI (8–11) and for the activation of nucleotides with 2AI (12–14). Given that 2AI is a potentially prebiotic activating moiety, the mechanism by which 2AI-activated nucleotides undergo template-directed polymerization is of considerable interest.

Our laboratory has extensively characterized nonenzymatic primer extension with 2AI-activated monomers as a model of prebiotic template copying (15–17). Nonenzymatic RNA primer extension with 2AI-activated substrates involves two chemical steps. First, two 2AI-activated monomers react with each other to form an imidazolium-bridged dinucleotide (18,19) (or a mononucleotide bridged to an oligonucleotide), which binds to the template strand adjacent to the primer. In the second step, the 3′-hydroxyl of the terminal primer nucleotide attacks the adjacent phosphate of the bridged intermediate (Fig. 1, *A* and *B*), leading to the formation of a new phosphodiester bond and displacing an activated nucleotide (or oligonucleotide) as the leaving group. Hence, the primer is extended one nucleotide at a time whether the intermediate is an imidazolium-bridged dinucleotide or a mononucleotide bridged to an oligonucleotide (21). Kinetic studies have shown that the intermediate can form both in solution and on the template (19).

Our understanding of nonenzymatic primer extension has benefited greatly from high-resolution crystal structures of RNA primer/template complexes with a template-bound imidazolium-bridged guanosine dinucleotide (G*G). In such complexes, the G*G dinucleotide is bound to the template through two canonical Watson-Crick basepairs (20). A well-ordered G*G bridged dinucleotide was observed next to the terminal primer nucleotide, with a distance of 4.6 Å between the 3′-hydroxyl and the reactive phosphate of the nucleotide in the +1 position. We were subsequently able to observe both the formation of the imidazolium-bridged intermediate from activated monomers and its subsequent reaction with the primer in a series of time-resolved crystal structures (20). Although it has been informative to observe the two-step process of primer extension by crystallography, many aspects of the reaction mechanism remain unresolved or entirely unseen.

Perhaps the least well understood aspect of the primer-extension reaction is the role of the catalytic metal ion, typically Mg^{2+}. In our previous detailed kinetic studies of primer extension, the reaction does not proceed in the absence of Mg^{2+} and is initiated by the addition of Mg^{2+} at concentrations in the range of 100 mM (19). Some earlier studies have used significantly higher concentrations of Mg^{2+} to achieve optimal rates (22–24). Such high concentrations are far beyond what is needed to stabilize the primer-template duplex or even the binding of the imidazolium-bridged intermediate. Thus, it has been widely assumed that Mg^{2+} plays a critical catalytic role, but one

in which binding of the catalytic Mg^{2+} ion in the reaction center is extremely weak. Presumably because of this weak binding, none of the crystal structures determined to date have captured Mg^{2+} coordinated to functional groups in the reaction center. Additionally, while crystal structures have provided a three-dimensional view of the mechanism of primer extension, crystal lattice interactions may suppress structural fluctuations that occur in solution. Structures solved to date have been determined using constructs in which critical template residues are restricted to a single sugar conformation using conformationally locked nucleotides (25,26).

How might Mg^{2+} ions catalyze nonenzymatic primer extension? Mechanistic studies have shown that Mg^{2+} ions play vital roles in ribozyme catalysis by stabilizing leaving groups, activating nucleophiles, and coordinating nonbridging phosphate oxygens (27). In principle, Mg^{2+} ions could catalyze primer extension in similar ways. For example, inner-sphere coordination with the oxygen of the 3′-hydroxyl would significantly decrease the pK_a of that hydroxyl, thus favoring its deprotonation and formation of a metal-alkoxide nucleophile (28). Alternatively, Mg^{2+} coordination with a nonbridging phosphate oxygen of the bridged dinucleotide would make the phosphorus atom more electrophilic. Finally, a bridging interaction between the 3′-hydroxyl and the adjacent phosphate could decrease electrostatic repulsion between the alkoxide nucleophile and the phosphate being attacked, thereby decreasing the O3′-P distance. This is an attractive possibility, since phosphodiester bond formation involves a decrease in the O3′-P distance of about 3 Å (from 4.6 Å to 1.7 Å). Understanding how this occurs will require an appreciation of the dynamic nature of the primer-extension reaction site as well as sophisticated quantum-mechanical (QM) simulations of the reaction pathway.

Computational studies involving molecular dynamics (MD) simulations have played a key role in understanding RNA structure, dynamics, and functions involved in processes at various lengths and timescales (29) such as basepair opening (30), sugar puckering and bond rotations (31), dynamics of tetraloop structures (32), ion binding (33,34), the mechanistic role of metal ions (35,36) and riboswitch conformational changes (37). Here we study the possible mechanistic roles of Mg^{2+} in nonenzymatic primer extension. In this study, we have used MD simulations to explore the potential roles of the catalytic metal ion through its effects on the structure and dynamics of the RNA primer/template/bridged-intermediate complex. Our simulations provide an atomistic perspective of the pre-catalytic Mg^{2+}-free state and of several different models of Mg^{2+}-bound ground states. Our MD simulations lead to testable predictions of the metal ion coordination state that is most favorable for primer extension. Mechanistic and structural insights into how the metal ion stabilizes the catalytically competent structure may facilitate further improvements in the efficiency, extent, and accuracy of nonenzymatic primer extension.

MATERIALS AND METHODS

A computational model of a 12-bp RNA duplex (5′-GGAUG G*G GUAGG-3′/5′- CCUACCCCAUCC-3′) was generated using the nab scripting language (38), where G*G represents an imidazolium-bridged guanosine dinucleotide intermediate that is bound to the -CC- template nucleotides (Fig. 1, *A–C*). We chose a G*G bridged dinucleotide and flanking guanosine residues to mimic the sequence used for crystallographic studies (20). The sequence design includes G-C terminal basepairs to stabilize the termini of the RNA duplex. The G*G bridged dinucleotide was placed within the RNA duplex by aligning the generated RNA duplex model used in our work with a previously solved crystal structure (PDB: 6C8E) (20). These structures were aligned using Tcl scripting in VMD (39). The 5-nt oligomer downstream of the bridged dinucleotide acts as a helper oligonucleotide. Downstream helper oligonucleotides have been previously suggested to contribute to primer extension by favoring a pre-organized reactive conformation of the primer-template-intermediate complex (21,25) and also by stabilizing the RNA duplex.

In each MD simulation, the RNA complex was placed in a cubic periodic box with at least 15 Å to the box boundaries and was neutralized by adding magnesium ions and a further 0.15 M $MgCl_2$ in the solvent. The standard CHARMM36 (40,41) force field was used to represent the nucleic acid and ions. The force-field parameters of the imidazolium-bridged dinucleotide were developed on the basis of the CHARMM General Force Field (CGenFF) (42) through analogy to existing parameters (Table S1). All oligonucleotide strands are capped with 5TER and 3TER residues within the VMD plugin psfgen on the 5′ and 3′ end, respectively, except in the case of the unprotonated 3′ end of the primer strand where after removal of the hydrogen, charge was distributed to the 3′ oxygen and neighboring atoms as previously described (43). The TIP3P water model was used to represent explicit water molecules.

The NAMD 2.14 program (44) was used to perform MD simulations. Nonbonded interactions were smoothly switched off at 10–12 Å, and long-range interactions were calculated using the particle-mesh Ewald (PME) method. For all simulation steps, bond distances involving hydrogen atoms were fixed using the SHAKE algorithm. Minimization was done for 10,000 steps followed by 250-ps equilibration at 300 K in the *NVT* ensemble. Subsequent simulations were performed in the *NpT* ensemble at 1 atm using a hybrid Nosé-Hoover Langevin piston method, and temperature was controlled using Langevin dynamics with a damping coefficient of $\gamma = 1$. The O3′-P and O2′-P distances were restrained to those observed in the crystal structure (PDB: 6C8E (20))—4.6 Å and 6.5 Å when the 2-NH_2-Im group of the imidazolium bridge faces the major groove and 4.6 Å and 6.0 Å when the 2-NH_2-Im group of the imidazolium bridge faces the minor groove—for the first 5 ns of the *NpT* simulations (Fig. S1). Restraints were applied using the NAMD collective variables module (45). This initial simulation stage was discarded, and the following 200-ns run was used for all analysis, resulting in a total of 5 ns of equilibration and 200 ns of production MD. Convergence during equilibration was confirmed by tracking root-mean-squared deviation (RMSD) (Fig. S2). Simulations were run with a 2-fs timestep, and coordinates were saved every 10 ps.

To place a Mg^{2+} ion in the reaction center, we extracted frames with the smallest O3′-P distance from our simulations without Mg^{2+} and then introduced a Mg^{2+} ion within 2 Å of the O3′ atom and either the R_p or S_p oxygen atoms on the reactive phosphate of the bridged dinucleotide using Packmol (46). For an overview of simulation systems, see Table S2.

We performed five replicates for all MD systems yielding a total of 1-μs simulation time for each system (Sims1–8, Table S2). Overall, we analyzed ≈ 7 μs of simulation data in the current work. To prevent basepair fraying during the simulations, we applied weak restraints on the distances d(N4,O6), d(N3,N1), and d(O2,N2) on the terminal G-C basepairs using the

NAMD collective variables module (45). Canonical RNA geometries were also maintained by applying weak restraints between the C1′ atoms of the primer/template and helper/template nucleotides 2 nt upstream and downstream of the nicked backbone.

Structural analysis of the MD trajectories was performed using CPPTraj V5.1.0 (47), MDTraj 1.9.4 (48), and VMD (39). RMSD was calculated by superposing the simulation frame onto the first frame of the simulation trajectories prior to minimization. RMSD values were calculated for all nucleic acid atoms and bridged dinucleotide atoms. RMSD of the reaction center was computed using the G*G bridged dinucleotide and the 3′-terminal primer guanosine. Sugar puckering was calculated from the five torsion angles of the five-membered sugar ring. The torsion angles were converted to the pseudorotation phase angle and amplitude parameters based on the Altona-Sundaralingam definition (49). Each point on the circular histogram plots corresponds to a value of the phase angle (0°–360°) moving clockwise from a vertical value of 0° and the amplitude of the pucker (0°–70°), which increases radially from the center of the plot. Ribose rings avoid a planar conformation and are usually present in the C2′-endo or C3′-endo conformation.

The CHARMM force field has been considered by some to be less accurate than the AMBER force field for MD simulations of nucleic acids (29). We therefore carried out control simulations on the same major-groove-facing-2AI configuration as described above, starting from the same initial coordinates but using the AMBER OL3 force field (50) to simulate the RNA duplex primer, template, and helper nucleotides (Sim9). Parameters were converted to AMBER using the CHARMM-GUI Force Field Converter (51,52), and simulations were run using NAMD 2.14 using the TIP3P explicit model of water and 12-6-4 ion force field. Bonded parameters for the imidazolium-bridged dinucleotide, including bonds, angles, and dihedrals, were taken from those generated in CGenFF. Nonbonded parameters for the imidazolium group were generated using OpenFF, while OL3 parameters were used to represent the remaining nonbonded terms for the bases of the imidazolium-bridged dinucleotide. The final system was minimized for 10,000 steps, followed by 250 ps of equilibration in which the nucleic acid backbone heavy atoms were constrained with a force constant of 1 kcal mol^{-1} $Å^{-2}$ and non-backbone nucleotide heavy atoms were constrained with a force constant of 0.5 kcal mol^{-1} $Å^{-2}$. Production MD was performed in the *NpT* ensemble at 1 atm using a hybrid Nosé-Hoover Langevin piston method. Simulation parameters, including temperature and pressure control, PME, hydrogen atom constraints, and timestep were implemented exactly as above, except for a 1–4 scaling term of 0.833333, a cutoff of 9 Å, and rigid tolerance of 0.0005, as suggested by NAMD developers when using the AMBER force field (ks.uiuc.edu). Analysis of the control simulation was performed as described above.

Finally, the lack of polarization or charge-transfer effects in standard force fields can result in inaccurate thermodynamic and kinetic properties of Mg^{2+} binding in aqueous systems (29,53). To better characterize the RNA backbone-Mg^{2+} interaction in our simulations and to validate the classical simulations, we carried out mixed QM/molecular mechanical (QM/MM) simulations on the same major-groove-facing-2AI with S_p and deprotonated O3′-oxygen bound Mg^{2+} configuration as described above using NAMD's QM/MM interface ((54); Sim10). The 3′ primer sugar, 2AI-bridged backbone up to the 5′ carbons of each monomer in the bridged dimer, the interacting Mg^{2+}, and four inner-sphere coordinating water molecules were all treated at the QM level (49 atoms in total; Fig. S3 *A*), resulting in a multiplicity of 1 and no net charge for the QM region. The rest of the system was treated at the classical level using the CHARMM force field as described above. The QM calculations were performed by ORCA (55) using the HF-3c semi-empirical Hartree-Fock method (56). The HF-3c method was developed for relatively large biomolecular systems, with geometry and energy predictions showing a high level of agreement with large-basis methods (56), and thus is an ideal choice for the current study with a reasonable trade-off between accuracy and computational cost. Interactions between the QM and MM regions were treated with an electrostatic embedding scheme, and covalent bonds split at the QM/MM boundary were treated with a charge-shifting method. Charge distribution in the QM region was taken from ORCA and updated at every step using the Mulliken charge calculation mode. Other than the use of a 0.5-fs timestep, parameters for the MM region were identical to those described above for CHARMM simulations. Analysis of the QM/MM simulation was performed as described above. Self-consistent field electron density and electrostatic potential maps were generated using the orca_plot command and the molecular orbitals output by ORCA and plotted with an isovalue of 0.085. Gibbs free energy for the QM region was calculated in ORCA using the HF-3c method and the universal continuum solvation model of implicit solvent using coordinates from the final frame of Sim10 with and without Mg^{2+} and coordinating waters. A second simulation was performed for ≈ 10 ps without the reactive Mg^{2+} and coordinating waters (Sim11) using coordinates from the equilibrated Sim2, resulting in a quantum region of 36 atoms and a -2 charge. Simulation parameters and analysis methods were identical to those described above. To observe the repulsive effect of removing the coordinating Mg^{2+}, a third QM/MM simulation was performed starting from the same coordinates as Sim10 but with the Mg^{2+} and coordinating waters removed, again giving a quantum region of 36 atoms and -2 charge (Sim12).

RESULTS AND DISCUSSION

Effect of protonation state of the primer 3′-hydroxyl in the absence of Mg^{2+}

Previous work has suggested that the active nucleophilic species in nonenzymatic primer extension is a 3′-alkoxide. This conclusion was supported in part by the absence of any significant solvent deuterium isotope effect, which is consistent with the necessary proton transfer occurring prior to the transition state. In addition, the pH dependence of the reaction rate has been interpreted as reflecting the metal-ion-dependent deprotonation of the 3′-hydroxyl (28). However, these experiments are indirect, and the possibility that the nucleophile is the protonated hydroxyl cannot be fully discounted. We have therefore modeled the reaction center with the 3′-hydroxyl either protonated or deprotonated in the ground state, which represents the structure prior to the initiation of the chemical reaction. In principle, the 3′-hydroxyl could be deprotonated by proton transfer to a Mg^{2+} coordinated with five water molecules and a hydroxide ion, such that O3′ would not be inner-sphere coordinated with the Mg^{2+} ion. Such an alkoxide would be more nucleophilic than a directly metal coordinated alkoxide and much more nucleophilic than a protonated 3′-OH (27). To provide atomistic insight into the dynamics of the ground state and structural implications of a deprotonated O3′, we first compared MD simulations in which O3′ was a protonated 3′-OH (Sim1) versus an O3′ alkoxide (Sim2), both in the absence of Mg^{2+}.

We first carried out simulations with the conformation of the bridged dinucleotide such that the 2-amino group of the 2AI was hydrogen bonded with the R_p oxygen atoms of the flanking phosphates (2-NH_2-Im:R_p, Fig. 1 *D*; Sim1), referred to as the major groove-facing conformation in (20). Overall, the structure in simulations when O3′ is protonated show low RMSD with respect to the starting

conformation, indicating excellent maintenance of the double helical structure (Fig. S2, *A* and *B*). The higher RMSD of the G*G bridged dinucleotide and reaction center when O3′ is deprotonated (Sim2) indicates that the deprotonated species makes the reaction center slightly more dynamic (Fig. S2, *C–E* and *G*), which could inhibit the formation of a pre-organized state necessary for reaction.

To further characterize the reaction center and understand the origin of the increased flexibility of the reaction center in the deprotonated case, we compared the distance between the O3′ atom of the terminal primer nucleotide and the P of the adjacent phosphate of the bridged dinucleotide (Fig. 1 *D*). Shorter distances, which would favor the primer-extension reaction, are observed when O3′ is protonated, while the median distance increases from 3.7 Å to 5.3 Å in the deprotonated case (Fig. 1 *E*). In our most recent crystal structure (PDB: 6C8E (20)), the corresponding distance is 4.6 Å. Crystal structures solved with G(5′)ppp(5′)G—an analog of the imidazolium-bridged dinucleotide—in the absence of helper oligos (PDB: 5UED (57)) show the distance between O3′ and the nearest phosphorous atom to be 4.1 Å. In the presence of helper oligos (PDB: 6AZ4 (25)) the corresponding distance is significantly shorter, 3.7 Å. Our protonated O3′-P distances lie within the range of those observed in these crystal structures, but this key distance increases significantly when the 3′-hydroxyl is deprotonated.

To examine the effect of the protonation state of the 3′-hydroxyl on the ribose conformation, we calculated the pseudorotation angles of the ribose sugar of the terminal primer nucleotide. Fig. S4 shows the simulated conformational ensemble projected on a circular plot where the phase angle increases clockwise from a vertical value of 0° and the puckering amplitude increases radially from the center. The resulting probability density plot shows that the most prominent conformation observed is the C3′-endo for both protonated and deprotonated O3′ simulations. During our MD runs, the ribose sugar exhibits reversible conformational switches to the C2′-endo conformation (Fig. 2, *C* and *D*). As seen in Fig. S5, this conformational change from C3′-endo to C2′-endo occurs through the O4′-endo conformation in most cases, while in others the transition occurs without visibly occupying an intermediate state. These apparently instantaneous transitions are likely the result of the change occurring on a timescale faster than the 0.01 ns DCD output frequency used. The C2′-endo conformation is observed for longer periods in the deprotonated case, and the time spent in this conformation increases from 3.5% when O3′ is protonated to 30% when O3′ is deprotonated. In contrast, the terminal primer nucleotide sugars in the crystal structure (PDB: 6C8E (20)) are all in the C3′-endo conformation (Fig. S4 *A*).

During the simulation runs, periods of high O3′-P distance are highly correlated with the primer ribose conformation switch to C2′-endo (Figs. 2, S6, and S7) in both protonated and deprotonated cases. It is unclear whether the increase in distance is a cause or consequence of the sugar conformation switch. Nevertheless, the correlation explains why modified primer 3′-nucleotides with sugars with an increased preference for the C3′-endo conformation are better at primer extension (26,58,59) because this conformation is associated with shorter distances of attack. The larger O3′-P distances and the prevalence of the C2′-endo sugar conformation observed when O3′ is deprotonated in our simulations suggest that deprotonation without a bound Mg^{2+} (while highly unlikely to occur) would be anti-catalytic. This observation is consistent with the possibility that the deprotonated O3′ primer is stabilized by coordination with a metal ion, which may act to overcome the electrostatic repulsion between the deprotonated O3′ and the phosphate it is attacking.

Orientation of the 2-aminoimidazole affects the O3′-P distance and angle of attack

To examine the effect of the orientation of the imidazolium moiety of the bridged dinucleotide on the geometry of the reaction center, we performed MD simulations beginning with the minor groove-facing conformation, in which the imidazolium 2-NH_2-Im group forms hydrogen bonds with the S_p oxygens of the flanking phosphates (2-NH_2-Im:S_p, Fig. 3 *A*). As before, we performed these simulations for both the protonated (Sim3) and deprotonated states (Sim4) of the primer 3′-hydroxyl. As in the major groove-facing orientation discussed above, the minor groove-facing orientation simulations show a smaller O3′-P distance of attack when O3′ is protonated (Fig. 3 *B*). When O3′ is deprotonated, the median distance increases by 1.7 Å, similar to the 1.6-Å increase seen in the major groove-facing simulations discussed previously.

The minor groove-facing orientation of the imidazolium group has relatively minor effects on the ribose pucker of the 3′-primer nucleotide. In both protonation states, the sugar is predominantly in the C3′-endo conformation (Fig. S8). However, when O3′ is deprotonated, the terminal primer sugar shows a slightly higher probability of a C3′-endo conformation as opposed to when O3′ is protonated, which is opposite to the trends observed for the major groove-facing imidazolium state (compare Figs. S4 and S8). The sugar conformation is also not as well correlated with the O3′-P distance. We hypothesize that the increased distance between the primer and the bridged dinucleotide (Fig. 3 *B*) allows the sugar pucker and the O3′-P distance to vary independently of each other. The first sugar of the bridged dinucleotide (G1) is predominantly C3′-endo for the simulations with a major groove-facing imidazolium, but predominantly C2′-endo for the minor groove-facing imidazolium orientation (Fig. S9 *A*), while the ribose of the second sugar (G2) is always predominantly C3′-endo (Fig. S9 *B*). Overall, in the four simulation systems discussed so far, the maximum probability for the C3′-endo

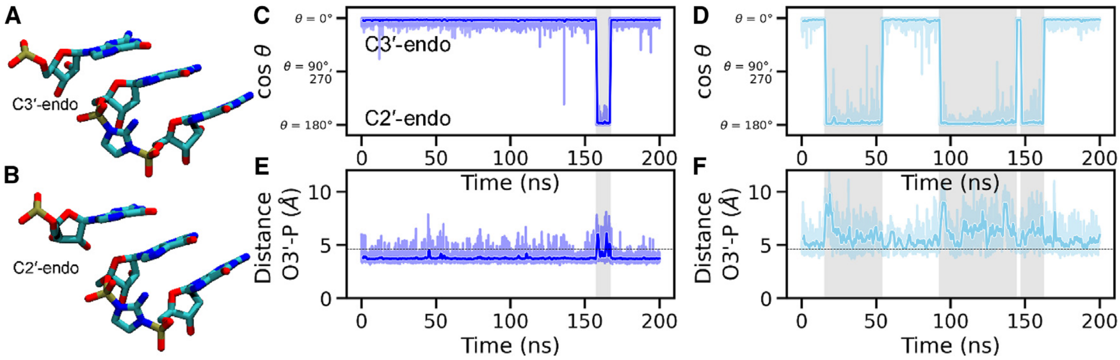

FIGURE 2 Correlation of sugar conformation and O3′-P distance during MD simulations without Mg^{2+}. (*A* and *B*) Molecular figures showing the conformation of the terminal primer sugar in the (*A*) C3′-endo conformation and (*B*) C2′-endo conformation. (*C* and *D*) Time series of the primer 3′-nt sugar pucker pseudorotation angle for the (*C*) 3′-hydroxyl (Sim1) and (*D*) 3′O^- (Sim2) simulation systems, without Mg^{2+} in the reaction center. Sugar pucker angles close to 0° correspond to the C3′-endo sugar conformation, and angles close to 180° correspond to the C2′-endo sugar conformation. Time series for cosine of the angle (cos θ) are shown for clarity, and angle (θ) values are indicated. (*E* and *F*) Time series of the distance between O3′ (primer) and P (bridged dinucleotide) for the (*E*) 3′-hydroxyl and (*F*) 3′O^- simulation systems, both without Mg^{2+} in the reaction center. The horizontal dashed lines indicate the crystal structure value. Intervals with larger distance values and C2′-endo sugar conformations are highlighted in gray. For all time-series plots, dark traces show the data averaged over a 1-ns window, while the lighter envelope shows the full range of the data recorded at 10-ps time steps in our simulations. All simulation replicates are shown in Figs. S6 and S7.

sugar conformation for all three ribose sugars is seen when the primer O3′ is protonated and the bridged dinucleotide is in the major groove-facing orientation.

The orientation of the imidazolium moiety also affects the angle of attack between the 3′-hydroxyl and the P–N bond to be broken during phosphodiester bond formation. A more favorable angle for in-line attack (closer to 180°) is primarily seen in the major groove-facing conformations (Fig. 3 *C*). This is in contrast to the crystal structures, where the angles of attack are 132° and 170° for the major groove-facing and minor groove-facing conformations, respectively. While the cause of this discrepancy is unclear, it may reflect an artifact of fitting both possible orientations into the same density in the crystal structures, while MD simulations provide insights into the relaxed conformations explored during the time course of the simulations.

The O3′-P distance, the terminal primer sugar pucker, and the angle of attack are key structural characteristics of the primer-extension reaction center. In summary, we observe a shorter O3′-P distance, increased C3′-endo conformation of the terminal primer sugar, and a higher than $\approx 150°$ angle of attack when the imidazolium 2-NH_2-Im faces the major groove of the RNA duplex. All three parameters shift toward less catalytically favorable values when the 2-NH_2-Im faces the minor groove. Therefore, while both conformations are likely to occur, we suggest that the major groove-facing conformation of the bridged dinucleotide imidazolium is more favorable for phosphodiester bond formation.

While orientation of the imidazolium group influences the geometry of the reaction center, this orientation is in turn influenced by ionic interactions. Our MD simulations suggest that the major groove-facing conformation of the bridging 2-AI group may be stabilized by water-mediated coordination with a Mg^{2+} ion that is distinct from the catalytic Mg^{2+} coordinated by the 3′-hydroxyl. In a crystal structure (PDB: 6C8D (20)) in which two template-bound 2′-deoxyguanosine-5′-monophosphate monomers sit next to the primer in an RNA duplex, a metal ion (Sr^{2+} ion in PDB: 6CAB (20)) forms water-mediated contacts with N7 and O6 of the 3′-terminal primer guanosine and with the N7 atoms of the G1 and G2 template-bound monomers. Both inner-sphere and outer-sphere interactions of Mg^{2+} with the N7 atom of guanosine have been observed in a large number of solved RNA crystal structures (60). Interestingly, in our MD simulations, we observe water-mediated interactions between a Mg^{2+} ion and N7 of the first guanosine (G1) of the bridged dinucleotide, and with its O6 atom. Additionally, the same metal ion forms water-mediated interactions with the G2 nucleobase of the bridged dinucleotide, which supports its previously suggested role in the better copying chemistry of G*G as opposed to other nucleobases (Fig. S10 *A*). Moreover, it is possible that this Mg^{2+} may also form a water-mediated contact with the 2-amino group of the bridging imidazolium when it is in the major groove-facing conformation (Fig. S10 *A*). This imidazolium-ion contact is mediated through water molecules and does not occur when the imidazolium faces the minor groove (Fig. S10 *B*). Hence, we posit that a secondary metal ion may help to pre-organize the catalytically favorable major groove-facing conformation of the bridged dinucleotide.

To determine the extent to which the observed dynamics are dependent on the choice of force field, a 200-ns equilibration simulation without constraints was performed on the major groove-facing system with protonated O3′ using the AMBER force field (Sim9; see materials and methods for simulation details). For analysis, the simulation was split into two 100-ns subtrajectories to test for self-consistency (61). Compared to the first 100 ns of the first replicate

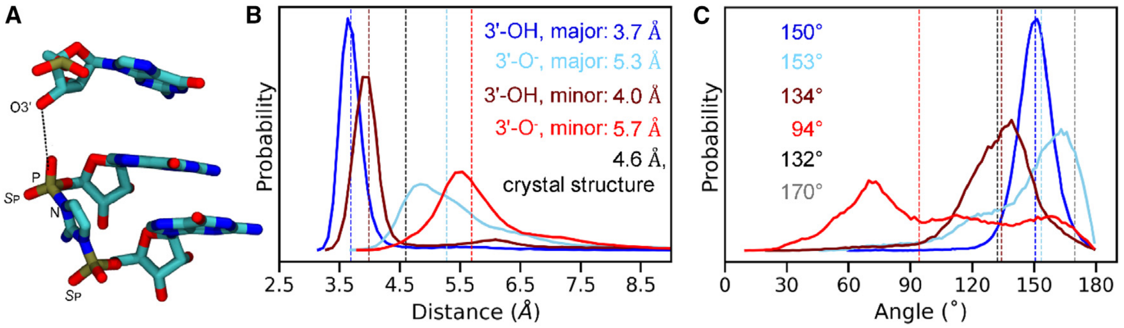

FIGURE 3 O3′-P distance and angle of attack during MD simulations in different 2AI conformations without Mg^{2+}. (*A*) Structure of the primer-extension reaction site, showing the last primer nucleotide and the G*G bridged dinucleotide with the imidazolium 2-NH_2-Im pointing toward the minor groove. Hydrogen atoms are not shown for clarity. The dashed line shows the O3′-P distance. (*B*) Probability distribution of the distance between O3′ and P atoms observed in the four simulation ensembles: major groove-facing 2-NH_2-Im conformations for 3′-hydroxyl without Mg^{2+} (Sim1; *dark blue*) and 3′O^- without Mg^{2+} (Sim2; *light blue*), and minor groove-facing conformations for 3′-hydroxyl without Mg^{2+} (Sim3; *brown*) and 3′O^- without Mg^{2+} (Sim4; *red*). Colored dashed lines show median values for the distance distributions. The black dashed line shows the crystal structure distance. (*C*) Probability distribution of the angle of attack, measured using the O3′-P-N atoms, in the four simulation ensembles. Dashed lines show median values for the distributions. The black and gray dashed lines show the crystal structure angle for the major groove-facing and minor groove-facing conformations, respectively.

simulated using the CHARMM force field on the same major groove-facing configuration, the AMBER system exhibited similar terminal primer sugar puckering (Fig. S11, *A–C*), RMSD of nucleic acid heavy atoms (Fig. S11 *D*), O3′-P distance (Fig. S11, *E* and *F*), O-P-N attack angle (Fig. S11 *G*), and stability of Watson-Crick base pairing in sites flanking the imidazolium-bridged dinucleotide (Fig. S11 *H*). While some variability is observed in the degree of fluctuation between systems, the probability distributions show similar mean values (Fig. S11, *D–F*, *insets*). Additionally, similar variability is observed between subtrajectories using the CHARMM force field (Fig. S6, *first and second rows*). Finally, the terminal primer sugar in the CHARMM simulation does exhibit a greater propensity for switching to a C2′-endo conformation compared to the AMBER simulation. However, this simulation replicate exhibited the highest conformational dynamics in the terminal primer sugar of all the CHARMM simulation replicates (Fig. S6), suggesting that the sugar conformational ensemble seen in the AMBER simulation is comparable to the CHARMM simulations in aggregate (Fig. S4 *A*). These parameters are summarized and compared for the AMBER and CHARMM systems in Fig. S11 *I*. Overall, these results suggest that the dynamic effects presented in previous and subsequent simulations are the result of differences in the systems being investigated and not the simulation methods used.

Effects of Mg^{2+} coordination to the primer O3′ and the adjacent phosphate of the bridged dinucleotide

To identify potentially important interactions of a Mg^{2+} ion in the reaction center, we introduced a Mg^{2+} ion with inner-sphere interactions with a deprotonated O3′ and either the S_p or R_p oxygen atoms on the reactive phosphate of the bridged dinucleotide. These systems will be referred to as 3′O^- Mg^{2+}@S_p, 2-NH_2-Im:R_p (Sim5; Fig. 4 *A*) and 3′O^- Mg^{2+}@R_p, 2-NH_2-Im:R_p (Sim6; Fig. 4 *B*), respectively. We also performed simulations with the Mg^{2+} coordinated to the R_p oxygen and the 2-NH_2-Im facing the minor groove (3′O^- Mg^{2+}@R_p, 2-NH_2-Im:S_p, Sim7) (Fig. 4 *C*). When we attempted to start our MD simulations in the remaining 3′O^- Mg^{2+}@S_p, 2-NH_2-Im:S_p arrangement (Sim8), the Mg^{2+} ion spontaneously switched its coordination from the S_p to the R_p oxygen. In all replicates of our three Mg^{2+}-bound simulations, the ion forms stable bridging interactions between the terminal primer nucleotide and the bridged dinucleotide.

Comparison of Sim5 (3′O^- Mg^{2+}@S_p, 2-NH_2-Im:R_p) (*yellow curve* in Fig. 4 *D*) with the corresponding protonated simulation ensemble lacking a bound Mg^{2+} ion (Sim1; *dark-blue curve* in Fig. 4 *D*) shows a similar unimodal O3′-P distance distribution with the same median of 3.7 Å. The median O3′-P distance of 4.0 Å is slightly larger for Sim6 (3′O^- Mg^{2+}@R_p, 2-NH_2-Im:R_p; *purple curve* in Fig. 4 *D*). The 0.3-Å increase in the median O3′-P distance is too small to indicate that Mg^{2+} coordination with one or the other of the phosphate oxygen atoms would significantly favor the primer-extension reaction. However, in the latter case where both the 2-NH_2-Im and the Mg^{2+} are coordinated to the same oxygen atom, the reaction might be affected for other reasons, such as effects on the nucleophilicity of the 3′O^- or the electrophilicity of the reactive phosphorous atom.

Our previous simulations in the absence of bound Mg^{2+} showed that deprotonation of the 3′-hydroxyl is correlated with longer O3′-P distances. However, when there is a Mg^{2+} ion bridging the primer and the phosphate, the O3′-P distances in our MD simulations greatly decrease, with the median distance decreasing from 5.7 Å (*red curve* in Fig. 3 *B*) to 3.9 Å (*green curve* in Fig. 4 *D*). Hence,

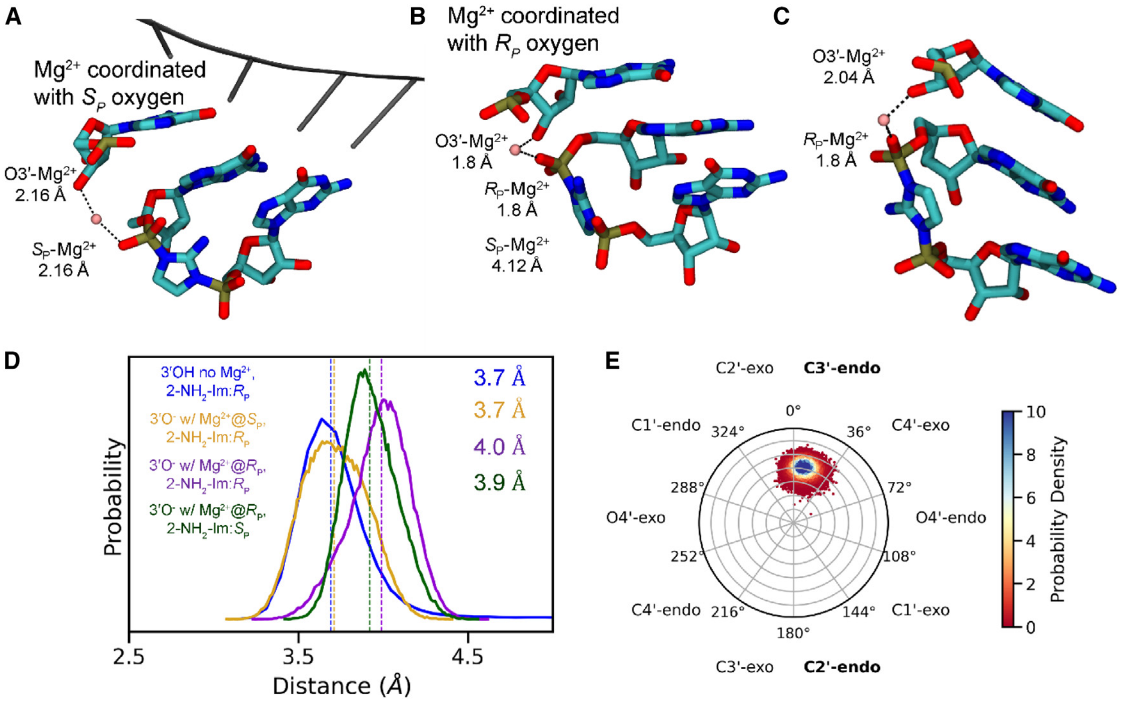

FIGURE 4 Representative snapshots showing plausible Mg^{2+} coordination geometry and their effects on dynamics. (*A*) Sim5 (3′O^- with $Mg^{2+}@S_p$, 2-NH_2-Im:R_p) with the template residues visualized in gray, (*B*) Sim6 (3′O^- with $Mg^{2+}@R_p$, 2-NH_2-Im:R_p), and (*C*) Sim7 (3′O^- with $Mg^{2+}@R_p$, 2-NH_2-Im:S_p). (*D*) Probability distribution of the distance between O3′ and P atoms observed in the three simulation ensembles with Mg^{2+} in the reaction center. The simulation ensembles are Sim5 (3′O^- with $Mg^{2+}@S_p$, 2-NH_2-Im:R_p; *yellow*), Sim6 (3′O^- with $Mg^{2+}@R_p$, 2-NH_2-Im:R_p; *purple*), and Sim7 (3′O^- with $Mg^{2+}@R_p$, 2-NH_2-Im:S_p; *green*). Distance probability distribution for 3′-hydroxyl without Mg^{2+}, 2-NH_2-Im:R_p (*dark blue*) is shown for comparison. Colored dashed lines show median values for the distance distributions. (*E*) Circular histogram of pseudorotation angles of the terminal primer nucleotide sugar for the Sim5 (3′O^- with $Mg^{2+}@S_p$, 2-NH_2-Im:R_p) simulation case. All Mg^{2+} coordination geometries with respect to the oxygen atoms are comparable, and Fig. S16 shows that the O3′-Mg^{2+}-O angles are close to 95° for all Mg^{2+} coordination geometries.

although deprotonation was anti-catalytic in the absence of Mg^{2+}, a bridging Mg^{2+} atom coordinating with the deprotonated O3′ overcomes this effect and plays an important role in preventing the primer and bridged dinucleotide from moving apart from each other. This effect of Mg^{2+} is independent of which of the oxygen atoms of the bridged dinucleotide the Mg^{2+} coordinates with, or the orientation of the imidazolium 2-NH_2-Im.

To further characterize the ground state of the primer/template/bridged-intermediate complex, we have also examined the terminal primer sugar conformation, angle of attack, and RMSD of the reaction center in the presence of Mg^{2+}. In addition to decreasing the O3′-P distance, the presence of a bridging Mg^{2+} ion is correlated with the terminal primer nucleotide sugar being almost exclusively in the C3′-endo conformation (Figs. 4 *E* and S12), which has been shown experimentally to favor primer extension (58,59). In simulations where the imidazolium 2-NH_2-Im group faces the major groove the G1 sugar pucker is predominantly C3′-endo, but when the imidazolium 2-NH_2-Im group faces the minor groove the G1 nucleotide sugar pucker conformation shifts toward being C2′-endo (Fig. S13 *A*), consistent with our simulations without Mg^{2+} in the reaction center (Fig. S9 *A*). In the simulation in which both the Mg^{2+} ion and the imidazolium 2-NH_2, are interacting with the R_p oxygen of the phosphate, the G1 sugar adopts a range of conformations including C4′-exo, C1′-exo, and C2′-endo, with very low probability of C3′-endo sugar puckering (Fig. S13 *A*). As seen in simulations without Mg^{2+}, the sugar pucker of the G2 nucleotide does occasionally adopt the C2′-endo conformation, but the C3′-endo conformation shows a larger population density (Fig. S13 *B*). Recent work has shown that the primer-extension reaction is faster when the bridged dinucleotide sugars are in the C3′-endo conformation (62). From our simulations, only Sim5 (3′O^- $Mg^{2+}@S_p$, 2-NH_2-Im:R_p) exhibits a primarily C3′-endo conformation for both G1 and G2.

Sim5 (3′O^- $Mg^{2+}@S_p$, 2-NH_2-Im:R_p) also shows a favorable angle for in-line attack (Fig. 5 *A*, median value of 148°, *yellow curve* in Fig. 5 *B*), which is comparable to the median value of 150° for the simulations with protonated O3′ without a Mg^{2+} ion (*dark-blue curve* in Fig. 5 *B*). All other simulation systems with Mg^{2+} show less favorable angles of attack. Overall, a reaction-center organization in which the imidazolium group faces the major groove and the Mg^{2+} ion forms inner-sphere contacts with the S_p oxygen of the reactive phosphate would appear to favor the primer-extension reaction. Moreover, a lower RMSD of the basepaired

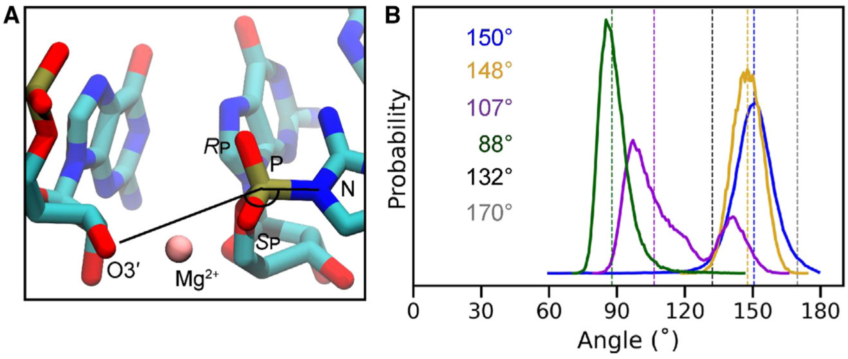

FIGURE 5 Effect of 2AI conformation and Mg^{2+} binding sites on angle of attack. (*A*) Molecular figure of the Sim5 (3′O$^-$ with Mg^{2+}@S_p, 2-NH_2-Im:R_p) system showing the angle of attack measured throughout the simulations. (*B*) Probability distribution of the angle of attack, measured among O3′-P-N atoms in four simulation ensembles: Sim1 (3′-hydroxyl without Mg^{2+}, 2-NH_2-Im:R_p; *dark blue*), Sim5 (3′O$^-$ with Mg^{2+}@S_p, 2-NH_2-Im:R_p; *yellow*), Sim6 (3′O$^-$ with Mg^{2+}@R_p, 2-NH_2-Im:R_p; *purple*), and Sim7 (3′O$^-$ with Mg^{2+}@R_p, 2-NH_2-Im:S_p; *green*). Dashed lines show median values for the distributions. The black and gray dashed lines show the crystal structure angle for the major groove-facing and minor groove-facing conformations, respectively.

primer/template and helper/template regions (Fig. S14 *A*) and the bridged dinucleotide (Fig. S14 *B*) region for Sim5 (3′O$^-$ Mg^{2+}@S_p, 2-NH_2-Im:R_p) suggest that the presence of the bridging metal ion in the reaction center has a global effect in stabilizing the primer-extension complex.

Interestingly, we also observed that when O3′ is deprotonated and no Mg^{2+} is present in the reaction center, the average O2′-P distance is less than the average O3′-P distance, which might suggest an increased probability of forming a 2′-5′ linkage. Previous studies from our laboratory have shown that primer extension by reaction with an imidazolium-bridged dinucleotide intermediate increases the proportion of 3′-5′ linkages formed, compared to reaction with an activated monomer (63). Our results suggest that through its role in stabilizing the conformation of the bridged dinucleotide, the metal ion also prevents the formation of the incorrect 2′-5′ extended product. This is further validated by the QM/MM simulation without Mg^{2+} (Sim11), in which a spontaneous reaction between the 2′-oxygen and reactive phosphate occurred in $\approx$5.5 ps. During this reaction, the O2′ proton was simultaneously transferred to the deprotonated 3′ oxygen (Fig. S15 *A*). This reaction was not observed in the entire 100-ps simulation in which Mg^{2+} is present. Furthermore, the predicted favorable metal coordination with the S_p oxygen atom of the bridged dinucleotide (*yellow symbols* in Fig. S15 *B*) typically shows a more significant difference between the average O3′-P and O2′-P distances, increasing the likelihood of formation of the 3′-5′ linkage.

To further validate models used for the interaction between RNA and Mg^{2+}, a 100-ps QM/MM simulation was performed on the 3′O$^-$ Mg^{2+}@S_p, 2-NH_2-Im:R_p system, with the reaction center modeled at the HF-3c semi-empirical Hartree-Fock level (Sim10; Fig. S3 *A*; see materials and methods for simulation details). The QM/MM simulation exhibited similar O-P-N angle (Fig. S3 *B*) compared to the CHARMM simulations (Fig. 5 *B*), and average O3′-P distance was 3.8 Å in the QM/MM simulation (Fig. S3 *C*) compared to 3.7 Å in the CHARMM simulation (Fig. 4 *D*), suggesting that these parameters are well modeled without the effects of polarizability. The O3′-Mg^{2+}-O(S_p) angle was measured (Fig. S3, *D* and *E*) and was similar to the angle observed in the CHARMM simulations between the same three atoms (Fig. S16). Finally, the QM/MM system exhibited similar terminal primer sugar puckering (Fig. S3 *F*) compared to the CHARMM simulations (Fig. S12 *A*), although the QM/MM simulation exhibited a slight preference for a C4′-exo conformation relative to the CHARMM simulation. While the timescale limitations make it difficult to discern whether this is a favorable conformation or the system is in transition from one state to another, experiments and simulations have shown the C4′-exo conformation to be significantly populated in other RNA systems (64). A potential molecular explanation for this deviation can be found in the polarizability of the QM/MM system (Fig. S3, *G* and *H*), in which the calculated charge of the H2′ atom was 0.33 at the end of the QM/MM simulation, while the same atom had a fixed charge of 0.09 in the MM simulation. This resulted in a tighter interaction between the H2′ and O3′ in the QM/MM simulation (Fig. S3 *I*), which biases the geometry of the sugar toward a more planar configuration. Despite this difference in charge between the classical and QM/MM simulations, classical modeling of essential geometric parameters such as O3′-P distance, O-P-N angle, and O3′-Mg^{2+}-O(S_p) angle agree with the quantum calculations over the timescale sampled. These parameters are summarized for the QM/MM and classical system in Fig. S3 *J*.

In addition to validating the choice of force field, the polarizability of QM/MM simulations provide a more thorough opportunity to investigate the role of electrostatic repulsion and attraction in determining the geometry of the reaction center. A second QM/MM simulation was performed without Mg^{2+} in the reactive complex (Sim11), and the electrostatic potentials, O3′-P distances, and Coulombic forces were compared. The darker color of the electrostatic map exhibited by the system without Mg^{2+} and depletion of negative charge in the system with Mg^{2+} (Fig. S17, *A* and *B*) qualitatively suggest a reduction of electrostatic repulsion when Mg^{2+} is present, and this manifests as a shorter

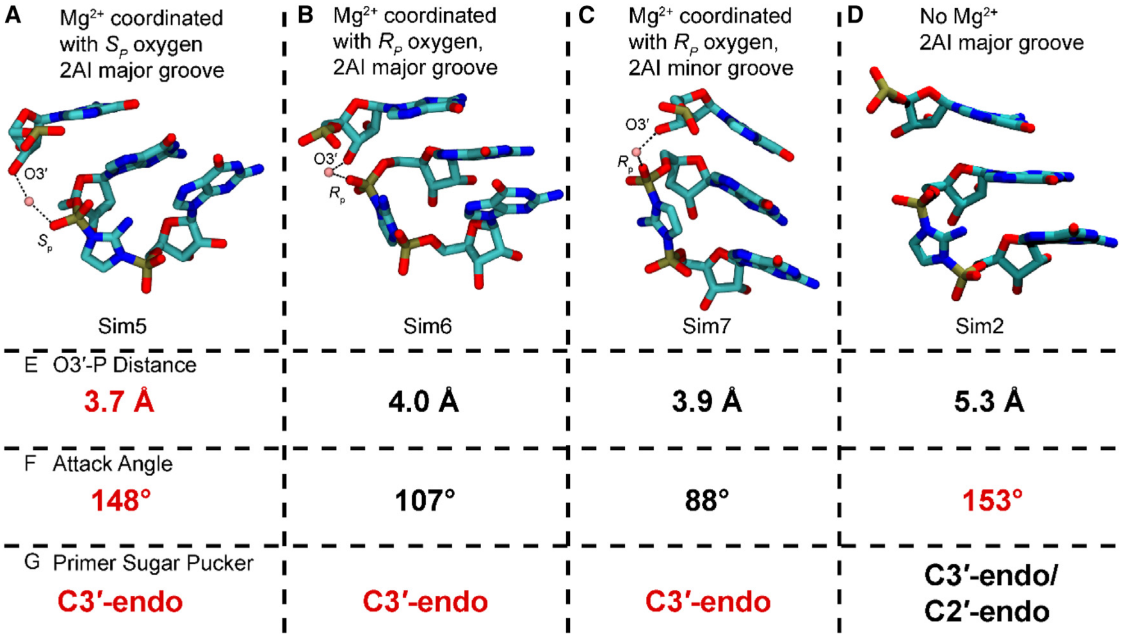

FIGURE 6 Summary of simulation systems and associated measured parameters. (*A–D*) Molecular figures of the indicated systems showing the 2AI-bridged dimer, terminal primer nucleotide, and Mg^{2+} when present. (*E–G*) Average values for the indicated parameters measured during the simulations. Red values indicate parameters that are most favorable to the primer-extension reaction.

O3′-P distance (Fig. S17, *C* and *D*). Additionally, the presence of Mg^{2+} results in a lower Gibbs free energy and electronic energy for the reaction center calculated from the same geometry with and without Mg^{2+} (Fig. S17, *A* and *B*). Coulombic force was calculated based on the charges and distances between O3′ oxygens, S_p oxygens, reactive phosphates, and Mg^{2+} when present for each respective simulation. The repulsion between the O3′ oxygen and S_p oxygen was similar for Sim10 and Sim11 despite a larger O3′-P distance in the system without Mg^{2+}, while the attraction between the O3′ oxygen and the reactive phosphate was larger when Mg^{2+} was present (Fig. S17 *E*). In the system where Mg^{2+} was removed (Sim12), the Coulombic force begins at the same value as for Sim10, but the O3′-P distance immediately increases and within ≈ 1 ps reaches equilibrium. During this time, the Coulombic force increases for O3′-P attraction and decreases for O3′-O(S_p) repulsion to values similar to those in Sim11 (Fig. S17 *E*). When Mg^{2+} is present (Sim10), the Coulombic force between the O3′ and S_p oxygens and the Mg^{2+} is strongly negative (Fig. S17 *F*). Collectively, these results strongly suggest that reduction of electrostatic repulsion may be an important role for Mg^{2+} in catalyzing nonenzymatic primer extension.

The structural features expected for optimal reaction-center geometry such as short O3′-P distance, C3′-endo sugar conformations, in-line angle of attack, and 3′-5′ regioselectivity are either absent or not present for extended timescales in Sim7 (3′O$^-$ Mg^{2+}@R_p, 2-NH_2-Im:S_p) and Sim6 (3′O$^-$ Mg^{2+}@R_p, 2-NH_2-Im:R_p). In the latter case, the hydrogen bond between the 2-NH_2-Im and the R_p oxygen is likely to be weakened due to the Mg^{2+} being coordinated with the same oxygen atom (Fig. S18 *E*). Thus, Mg^{2+} coordination with the S_p oxygen and 2-NH_2-Im hydrogen bonded to the R_p oxygen—the major groove-facing orientation—is likely to be the preferred geometry for the pre-organization of the reaction center.

CONCLUSION

Understanding the structure and dynamics of the primer-extension reaction center may offer insight into how to improve the rate (18) and fidelity (65) of primer-extension. In the present study, we performed MD simulations of a primer/template/bridged dinucleotide complex in the absence or presence of a catalytic Mg^{2+} in the reaction center. We modeled the system with both a protonated and a deprotonated 3′-hydroxyl, and with both likely orientations of the imidazolium moiety of the bridged dinucleotide intermediate. Only one conformation tested, Sim5 (3′O$^-$ Mg^{2+}@S_p, 2-NH_2-Im:R_p), exhibited favorable results for all main parameters analyzed (Fig. 6). From these results, we suggest that this represents a plausible structural model of the primer-extension reaction complex.

By analogy with the mechanism of enzyme catalyzed primer extension, one possible model for nonenzymatic primer extension is that inner-sphere coordination of the 3′-hydroxyl with Mg^{2+} lowers the pK_a of the hydroxyl, thereby facilitating its deprotonation to form an alkoxide nucleophile. Our MD simulations are consistent with a role for a bridging Mg^{2+} in overcoming the electrostatic

repulsion between the ionized $O3'^{-}$ and the negatively charged phosphate. In our simulations, the median distance from the primer $O3'^{-}$ to the phosphorous atom of the adjacent phosphate is 3.7 Å in the presence of Mg^{2+} in the reaction center, but much greater in the absence of a coordinated Mg^{2+} ion. Along with the organizing role of Mg^{2+} in the reaction center, the major groove-facing conformation of the imidazolium moiety of the bridged dinucleotide is correlated with an increased fraction of the C3′-endo sugar conformation and a higher in-line angle of attack. Our comparison of Mg^{2+} coordination with either of the two nonbridging phosphate oxygen atoms, S_p and R_p, of the bridged dinucleotide suggests that an inner-sphere coordination with the S_p oxygen atom is structurally favorable for the catalytic step. We have recently tested our predicted $3'O^{-}$-with-Mg^{2+}@S_p coordination by phosphorothioate metal rescue experiments (36,66,67), providing experimental evidence that the $3'O^{-}$-with-Mg^{2+}@S_p coordination is preferred.

Based on studies of primer extension with modified 3′-terminal sugars, Giurgiu et al. proposed that the primer-extension reaction involves an increase in the puckering amplitude of the terminal sugar as the O3′ atom attacks the adjacent phosphorous atom of the bridged dinucleotide (26). In some of our MD simulations we do see an association of larger puckering amplitude with configurations with a shorter O3′-P distance. Interestingly, this trend is strongest in the system proposed above to be most favorable for primer extension, i.e., Sim5 ($3'O^{-}$ with Mg^{2+}@S_p, 2-NH_2-Im:R_p), where Mg^{2+} is coordinated to the S_p oxygen and the amine of the bridging 2-AI is hydrogen bonded with the R_p phosphate oxygen (Figs. S19 and S20 *A*). Since modified sugars can significantly impact the rate of the primer-extension reaction, future computational studies incorporating sugars such as ANA, DNA, and LNA in the primer/template/bridged dinucleotide complex may reveal the contribution, if any, of increasing sugar pucker amplitude and structural factors related to the rate of the primer-extension reaction (26,68–70). Because transient intermediate conformations cannot be sampled efficiently through conventional MD, alternative biased simulation strategies may provide further insights into the primer-extension reaction. In addition, our current study is limited by the choice of force field (29) and does not consider the subsequent steps on the reaction pathway such as O3′-P bond formation, expulsion of Mg^{2+} from the reaction center, and the structural dynamics of the leaving group, all of which will require more extensive QM studies using QM/MM techniques.

In summary, we have used MD simulations to examine a set of models of the primer-extension reaction center in which a Mg^{2+} ion has been placed in alternative coordination geometries. We predict a specific catalytically favorable coordination of the metal ion within the reaction center. This prediction can be tested by thiol-metal substitution experiments in combination with conventional and time-resolved X-ray crystallographic studies. We suggest that the catalytic Mg^{2+} ion may play multiple roles including facilitating deprotonation of the 3′-hydroxyl and increasing the electrophilicity of the reactive phosphorous. We propose that the catalytic Mg^{2+} also plays an electrostatic role in overcoming repulsion between the negative charge of a deprotonated O3′ and the phosphate being attacked and plays an organizational role in stabilizing the conformation of the bridged dinucleotide and its position relative to the terminal primer nucleotide.

SUPPORTING MATERIAL

Supporting material can be found online at https://doi.org/10.1016/j.bpj.2024.04.032.

AUTHOR CONTRIBUTIONS

The manuscript was written through contributions of all authors. All authors have given approval to the final version of the manuscript.

ACKNOWLEDGMENTS

Portions of this research were conducted on the O2 High Performance Compute Cluster, supported by the Research Computing Group, at Harvard Medical School. See https://it.hms.harvard.edu/our-services/research-computing for more information. Remaining simulations were performed using HPC resources on the Midway3 supercomputer at the Research Computing Center at the University of Chicago. We thank Dr. Rafal Szabla for his helpful comments and insights.

This work was supported by the Simons Foundation (grant no. 290363 to J.W.S.) and the National Science Foundation (grant no. CHE-1607034 to J.W.S.). J.W.S. is an Investigator of the Howard Hughes Medical Institute. Funding for open access charge: Howard Hughes Medical Institute.

DECLARATION OF INTERESTS

The authors declare no competing interests.

REFERENCES

1. Lohrmann, R., and L. E. Orgel. 1973. Prebiotic Activation Processes. *Nature*. 244:418–420.
2. Inoue, T., G. F. Joyce, …, C. B. Reese. 1984. Template-directed synthesis on the pentanucleotide CpCpGpCpC. *J. Mol. Biol.* 178:669–676.
3. Orgel, L. E. 1992. Molecular replication. *Nature*. 358:203–209.
4. Weimann, B. J., R. Lohrmann, …, J. E. Sulston. 1968. Template-Directed Synthesis with Adenosine-5′-phosphorimidazolide. *Science*. 161:387.
5. Inoue, T., and L. E. Orgel. 1982. Oligomerization of (guanosine 5′-phosphor)-2-methylimidazolide on poly(C): An RNA polymerase model. *J. Mol. Biol.* 162:201–217.
6. Jauker, M., H. Griesser, and C. Richert. 2015. Copying of RNA Sequences without Pre-Activation. *Angew. Chem.* 54:14559–14563.
7. Vogel, S. R., C. Deck, and C. Richert. 2005. Accelerating chemical replication steps of RNA involving activated ribonucleotides and downstream-binding elements. *Chem. Commun.* 39:4922–4924.

8. Vázquez-Salazar, A., G. Tan, …, A. Lazcano. 2017. Can an Imidazole Be Formed from an Alanyl-Seryl-Glycine Tripeptide under Possible Prebiotic Conditions? *Orig. Life Evol. Biosph.* 47:345–354.
9. Shen, C., L. Yang, …, J. Oró. 1987. Prebiotic synthesis of imidazole-4-acetaldehyde and histidine. *Orig. Life Evol. Biosph.* 17:295–305.
10. Oró, J., B. Basile, …, T. Yamrom. 1984. The prebiotic synthesis and catalytic role of imidazoles and other condensing agents. *Orig. Life.* 14:237–242.
11. Fahrenbach, A. C., C. Giurgiu, …, J. W. Szostak. 2017. Common and Potentially Prebiotic Origin for Precursors of Nucleotide Synthesis and Activation. *J. Am. Chem. Soc.* 139:8780–8783.
12. Ding, D., S. J. Zhang, and J. W. Szostak. 2023. Enhanced nonenzymatic RNA copying with in-situ activation of short oligonucleotides. *Nucleic Acids Res.* 51:6528–6539.
13. Mariani, A., D. A. Russell, …, J. D. Sutherland. 2018. A Light-Releasable Potentially Prebiotic Nucleotide Activating Agent. *J. Am. Chem. Soc.* 140:8657–8661.
14. Zhang, S. J., D. Duzdevich, …, J. W. Szostak. 2022. Freeze-thaw cycles enable a prebiotically plausible and continuous pathway from nucleotide activation to nonenzymatic RNA copying. *Proc. Natl. Acad. Sci. USA.* 119, e2116429119.
15. Li, L., N. Prywes, …, J. W. Szostak. 2017. Enhanced nonenzymatic RNA copying with 2-aminoimidazole activated nucleotides. *J. Am. Chem. Soc.* 139:1810–1813.
16. Zhou, L., S. C. Kim, …, J. W. Szostak. 2019. Non-enzymatic primer extension with strand displacement. *Elife.* 8, e51888.
17. Walton, T., and J. W. Szostak. 2017. A Kinetic Model of Nonenzymatic RNA Polymerization by Cytidine-5′-phosphoro-2-aminoimidazolide. *Biochemistry.* 56:5739–5747.
18. Walton, T., and J. W. Szostak. 2016. A Highly Reactive Imidazolium-Bridged Dinucleotide Intermediate in Nonenzymatic RNA Primer Extension. *J. Am. Chem. Soc.* 138:11996–12002.
19. Walton, T., L. Pazienza, and J. W. Szostak. 2019. Template-Directed Catalysis of a Multistep Reaction Pathway for Nonenzymatic RNA Primer Extension. *Biochemistry.* 58:755–762.
20. Zhang, W., T. Walton, …, J. W. Szostak. 2018. Crystallographic observation of nonenzymatic RNA primer extension. *Elife.* 7, e36422.
21. Prywes, N., J. C. Blain, …, J. W. Szostak. 2016. Nonenzymatic copying of RNA templates containing all four letters is catalyzed by activated oligonucleotides. *Elife.* 5, e17756.
22. Joyce, G. F., and L. E. Orgel. 1988. Non-enzymatic template-directed synthesis on RNA random copolymers: Poly(C,A) templates. *J. Mol. Biol.* 202:677–681.
23. Kanavarioti, A., C. F. Bernasconi, …, D. J. Alberas. 1989. Magnesium ion catalyzed P-N bond hydrolysis in imidazolide-activated nucleotides. Relevance to template-directed synthesis of polynucleotides. *J. Am. Chem. Soc.* 111:7247–7257.
24. Taifeng, W., and L. E. Orgel. 1992. Nonenzymatic template-directed synthesis on oligodeoxycytidylate sequences in hairpin oligonucleotides. *J. Am. Chem. Soc.* 114:317–322.
25. Zhang, W., C. P. Tam, …, J. W. Szostak. 2018. Structural Rationale for the Enhanced Catalysis of Nonenzymatic RNA Primer Extension by a Downstream Oligonucleotide. *J. Am. Chem. Soc.* 140:2829–2840.
26. Giurgiu, C., Z. Fang, …, J. W. Szostak. 2021. Structure–Activity Relationships in Nonenzymatic Template-Directed RNA Synthesis. *Angew Chem. Int. Ed. Engl.* 60:22925–22932.
27. Frederiksen, J. K., R. Fong, and J. A. Piccirilli. 2008. Chapter 8. Metal Ions in RNA Catalysis. *In* RSC Biomolecular Sciences. N. V. Hud, ed Royal Society of Chemistry, pp. 260–306.
28. Giurgiu, C., T. H. Wright, …, J. W. Szostak. 2018. A Fluorescent G-Quadruplex Sensor for Chemical RNA Copying. *Angew Chem. Int. Ed. Engl.* 57:9844–9848.
29. Šponer, J., G. Bussi, …, M. Otyepka. 2018. RNA Structural Dynamics As Captured by Molecular Simulations: A Comprehensive Overview. *Chem. Rev.* 118:4177–4338.
30. Zgarbová, M., M. Otyepka, …, P. Jurečka. 2014. Base Pair Fraying in Molecular Dynamics Simulations of DNA and RNA. *J. Chem. Theor. Comput.* 10:3177–3189.
31. Li, L., and J. W. Szostak. 2014. The Free Energy Landscape of Pseudorotation in 3′–5′ and 2′–5′ Linked Nucleic Acids. *J. Am. Chem. Soc.* 136:2858–2865.
32. Bottaro, S., P. J. Nichols, …, K. Lindorff-Larsen. 2020. Integrating NMR and simulations reveals motions in the UUCG tetraloop. *Nucleic Acids Res.* 48:5839–5848.
33. Yu, T., and S.-J. Chen. 2018. Hexahydrated Mg^{2+} Binding and Outer-Shell Dehydration on RNA Surface. *Biophys. J.* 114:1274–1284.
34. Ratnasinghe, B. D., A. M. Salsbury, and J. A. Lemkul. 2020. Ion Binding Properties and Dynamics of the bcl-2 G-Quadruplex Using a Polarizable Force Field. *J. Chem. Inf. Model.* 60:6476–6488.
35. Zhang, S., D. R. Stevens, …, S. Hammes-Schiffer. 2016. Assessing the Potential Effects of Active Site Mg^{2+} Ions in the glmS Ribozyme–Cofactor Complex. *J. Phys. Chem. Lett.* 7:3984–3988.
36. Ganguly, A., B. P. Weissman, …, D. M. York. 2020. Confluence of theory and experiment reveals the catalytic mechanism of the Varkud satellite ribozyme. *Nat. Chem.* 12:193–201.
37. Ucisik, M. N., P. C. Bevilacqua, and S. Hammes-Schiffer. 2016. Molecular Dynamics Study of Twister Ribozyme: Role of Mg^{2+} Ions and the Hydrogen-Bonding Network in the Active Site. *Biochemistry.* 55:3834–3846.
38. Macke, T. J., and D. A. Case. 1998. Modeling Unusual Nucleic Acid Structures. *ACS Symp. Ser.* 682:379–393.
39. Humphrey, W., A. Dalke, and K. Schulten. 1996. VMD: visual molecular dynamics. *J. Mol. Graph.* 14:33.
40. MacKerell, A. D., D. Bashford, …, M. Karplus. 1998. All-Atom Empirical Potential for Molecular Modeling and Dynamics Studies of Proteins. *J. Phys. Chem. B.* 102:3586–3616.
41. Denning, E. J., U. D. Priyakumar, …, A. D. MacKerell, Jr. 2011. Impact of 2′-hydroxyl sampling on the conformational properties of RNA: Update of the CHARMM all-atom additive force field for RNA. *J. Comput. Chem.* 32:1929–1943.
42. Vanommeslaeghe, K., E. Hatcher, …, A. D. Mackerell. 2010. CHARMM general force field: A force field for drug-like molecules compatible with the CHARMM all-atom additive biological force fields. *J. Comput. Chem.* 31:671–690.
43. Bignon, E., and A. Monari. 2022. Modeling the Enzymatic Mechanism of the SARS-CoV-2 RNA-Dependent RNA Polymerase by DFT/MM-MD: An Unusual Active Site Leading to High Replication Rates. *J. Chem. Inf. Model.* 62:4261–4269.
44. Phillips, J. C., D. J. Hardy, …, E. Tajkhorshid. 2020. Scalable molecular dynamics on CPU and GPU architectures with NAMD. *J. Chem. Phys.* 153, 044130.
45. Fiorin, G., M. L. Klein, and J. Hénin. 2013. Using collective variables to drive molecular dynamics simulations. *Mol. Phys.* 111:3345–3362.
46. Martínez, L., R. Andrade, …, J. M. Martínez. 2009. PACKMOL: a package for building initial configurations for molecular dynamics simulations. *J. Comput. Chem.* 30:2157–2164.
47. Roe, D. R., and T. E. Cheatham. 2013. PTRAJ and CPPTRAJ: Software for Processing and Analysis of Molecular Dynamics Trajectory Data. *J. Chem. Theor. Comput.* 9:3084–3095.
48. McGibbon, R. T., K. A. Beauchamp, …, V. S. Pande. 2015. MDTraj: A Modern Open Library for the Analysis of Molecular Dynamics Trajectories. *Biophys. J.* 109:1528–1532.
49. Altona, C., and M. Sundaralingam. 1972. Conformational analysis of the sugar ring in nucleosides and nucleotides. New description using the concept of pseudorotation. *J. Am. Chem. Soc.* 94:8205–8212.
50. Zgarbová, M., M. Otyepka, …, P. Jurečka. 2011. Refinement of the Cornell et al. Nucleic acids force field based on reference quantum chemical calculations of glycosidic torsion profiles. *J. Chem. Theor. Comput.* 7:2886–2902.
51. Jo, S., T. Kim, …, W. Im. 2008. CHARMM-GUI: A web-based graphical user interface for CHARMM. *J. Comput. Chem.* 29:1859–1865.

52. Lee, J., M. Hitzenberger, …, W. Im. 2020. CHARMM-GUI supports the Amber force fields. *J. Chem. Phys.* 153, 035103.

53. Lemkul, J. A., and A. D. Mackerell. 2016. Balancing the Interactions of Mg2+ in Aqueous Solution and with Nucleic Acid Moieties For a Polarizable Force Field Based on the Classical Drude Oscillator Model. *J. Phys. Chem. B.* 120:11436–11448.

54. Melo, M. C. R., R. C. Bernardi, …, Z. Luthey-Schulten. 2018. NAMD goes quantum: An integrative suite for QM/MM simulations. *Nat. Methods.* 15:351–354.

55. Neese, F., F. Wennmohs, …, C. Riplinger. 2020. The ORCA quantum chemistry program package. *J. Chem. Phys.* 152, 224108.

56. Sure, R., and S. Grimme. 2013. Corrected small basis set Hartree-Fock method for large systems. *J. Comput. Chem.* 34:1672–1685.

57. Zhang, W., C. P. Tam, …, J. W. Szostak. 2017. Insight into the mechanism of nonenzymatic RNA primer extension from the structure of an RNA-GpppG complex. *Proc. Natl. Acad. Sci. USA.* 114:7659–7664.

58. Wu, T., and L. E. Orgel. 1992. Nonenzymic template-directed synthesis on oligodeoxycytidylate sequences in hairpin oligonucleotides. *J. Am. Chem. Soc.* 114:317–322.

59. Kozlov, I. A., P. K. Politis, …, L. E. Orgel. 1999. Nonenzymatic Synthesis of RNA and DNA Oligomers on Hexitol Nucleic Acid Templates: The Importance of the A Structure. *J. Am. Chem. Soc.* 121:2653–2656.

60. Zheng, H., I. G. Shabalin, …, W. Minor. 2015. Magnesium-binding architectures in RNA crystal structures: validation, binding preferences, classification and motif detection. *Nucleic Acids Res.* 43:3789–3801.

61. Sawle, L., and K. Ghosh. 2016. Convergence of Molecular Dynamics Simulation of Protein Native States: Feasibility vs Self-Consistency Dilemma. *J. Chem. Theor. Comput.* 12:861–869.

62. Ding, D., L. Zhou, …, J. W. Szostak. 2022. Kinetic explanations for the sequence biases observed in the nonenzymatic copying of RNA templates. *Nucleic Acids Res.* 50:35–45.

63. Giurgiu, C., L. Li, …, J. W. Szostak. 2017. A Mechanistic Explanation for the Regioselectivity of Nonenzymatic RNA Primer Extension. *J. Am. Chem. Soc.* 139:16741–16747.

64. Steffen, F. D., M. Khier, …, R. K. O. Sigel. 2020. Metal ions and sugar puckering balance single-molecule kinetic heterogeneity in RNA and DNA tertiary contacts. *Nat. Commun.* 11:104.

65. Duzdevich, D., C. E. Carr, …, J. W. Szostak. 2021. Competition between bridged dinucleotides and activated mononucleotides determines the error frequency of nonenzymatic RNA primer extension. *Nucleic Acids Res.* 49:3681–3691.

66. Thaplyal, P., A. Ganguly, …, P. C. Bevilacqua. 2015. Inverse Thio Effects in the Hepatitis Delta Virus Ribozyme Reveal that the Reaction Pathway Is Controlled by Metal Ion Charge Density. *Biochemistry.* 54:2160–2175.

67. Fang, Z., L. T. Pazienza, …, J. W. Szostak. 2024. Catalytic Metal Ion-Substrate Coordination during Nonenzymatic RNA Primer Extension. *J. Am. Chem. Soc.* 22:47.

68. Kim, S. C., L. Zhou, …, J. W. Szostak. 2020. A Model for the Emergence of RNA from a Prebiotically Plausible Mixture of Ribonucleotides, Arabinonucleotides, and 2′-Deoxynucleotides. *J. Am. Chem. Soc.* 142:2317–2326.

69. Zhang, W., S. C. Kim, …, J. W. Szostak. 2021. Structural interpretation of the effects of threo-nucleotides on nonenzymatic template-directed polymerization. *Nucleic Acids Res.* 49:646–656.

70. Kim, S. C., D. K. O'Flaherty, …, J. W. Szostak. 2021. The Emergence of RNA from the Heterogeneous Products of Prebiotic Nucleotide Synthesis. *J. Am. Chem. Soc.* 143:3267–3279.

Biophysical Journal, Volume *123*

Supplemental information

Simulations predict preferred Mg^{2+} coordination in a nonenzymatic primer-extension reaction center

Shriyaa Mittal, Collin Nisler, and Jack W. Szostak

Supplemental Information

Simulations predict preferred Mg^{2+} coordination in a nonenzymatic primer extension reaction center

Shriyaa Mittal,[1,2,5] Collin Nisler,[4,5] Jack W. Szostak[,1,2,3,4]*

[1]Howard Hughes Medical Institute, Department of Molecular Biology, and Center for Computational and Integrative Biology, Massachusetts General Hospital, Boston, MA, 02114 USA; [2]Department of Genetics, Harvard Medical School, Boston, MA, 02115 USA; [3]Department of Chemistry and Chemical Biology, Harvard University, Cambridge, MA, 02138 USA; [4]Howard Hughes Medical Institute, Department of Chemistry, University of Chicago, Chicago, IL, 60637 USA

[5] These authors contributed equally to this work

* Corresponding author – Jack Szostak, jwszostak@uchicago.edu

Supplemental Figures

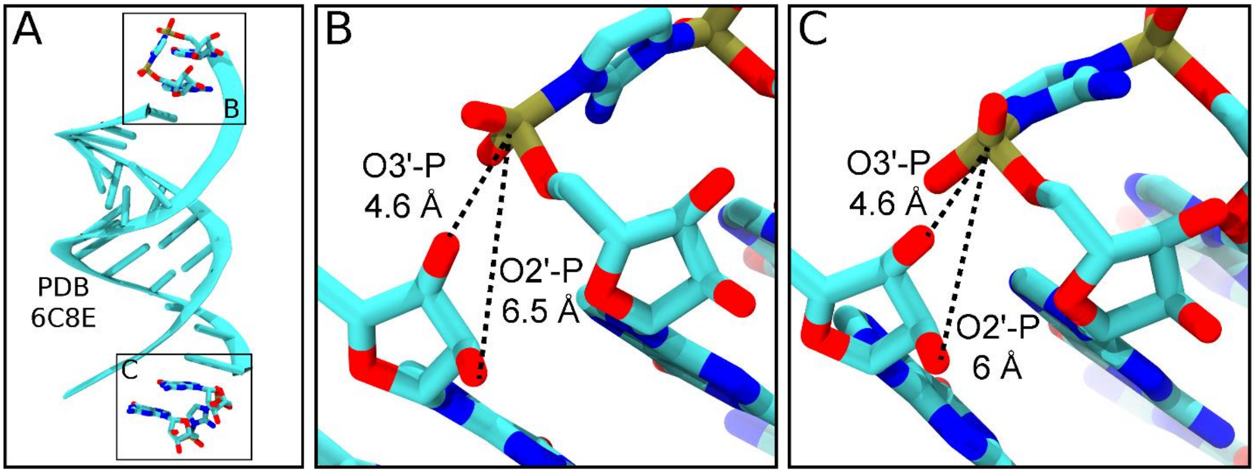

Figure S1. Distances observed in the crystal structure PDB 6C8E used for constraints during equilibration. (A) Structure of PDB 6C8E with RNA duplex shown in cartoon representation, and the 2AI-bridged dimer in licorice representation. (B) Zoomed in view from (A) showing O3'-P and O2'-P distances when 2AI is facing the major groove. (C) Zoomed in view from (A) showing O3'-P and O2'-P distances when 2AI is facing the minor groove.

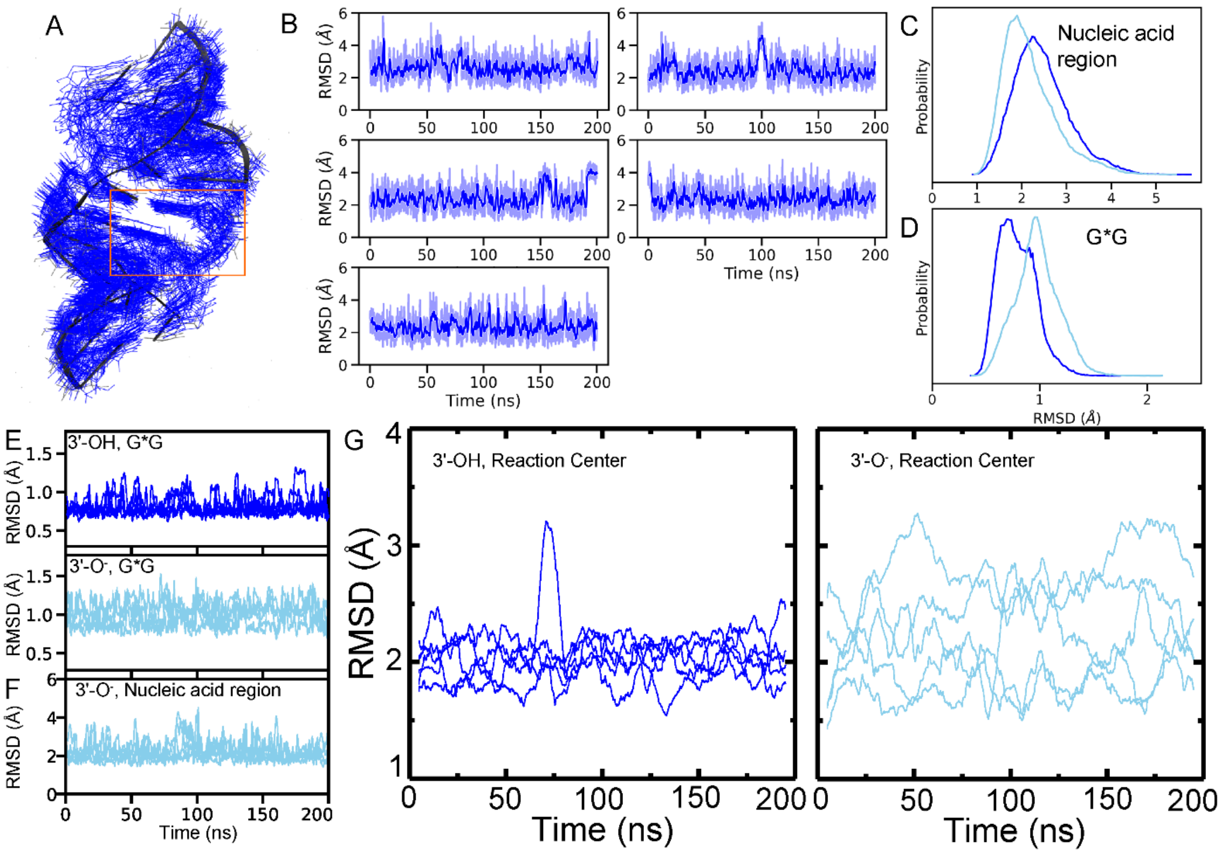

Figure S2. **RMSD of the duplex, bridged dinucleotide, and reaction center suggest increased dynamics with a deprotonated 3′-OH.** (A) Overlay of simulation snapshots over a 500 ns simulation trajectory of the complex with primer 3′-OH w/o Mg^{2+}. The first frame of the simulation can be seen partly as a dark gray ribbon representation. The G*G bridged dinucleotide is highlighted in an orange box. (B) Time series of root mean-squared deviation (RMSD) of the entire duplex for five simulation replicates. Comparing RMSD probability distribution for the (C) nucleic acid region and (D) G*G bridged dinucleotide (2AI-bridged dinucleotide only) for the simulation systems, 3′-OH w/o Mg^{2+} (Sim1; dark blue) and 3′O$^-$ w/o Mg^{2+} (Sim2; light blue). (E) Time series of the G*G dinucleotide RMSD of the 3′-OH w/o Mg^{2+} (Sim1a-e; left) and 3′O$^-$ w/o Mg^{2+} (Sim2a-e; right) simulation systems for all simulation replicates showing the 1 ns running averages. (F) Time series of the nucleic acid region RMSD of the 3′O$^-$ w/o Mg^{2+} (Sim2a-e) simulation system for all simulation replicates showing the 1 ns running averages. (G) Time series of the reaction center RMSD, comprised of the G*G bridged dinucleotide and 3′-terminal primer guanosine, of the 3′OH w/o Mg^{2+} (Sim1; left), and the 3′O$^-$ w/o Mg^{2+} (Sim2; right).

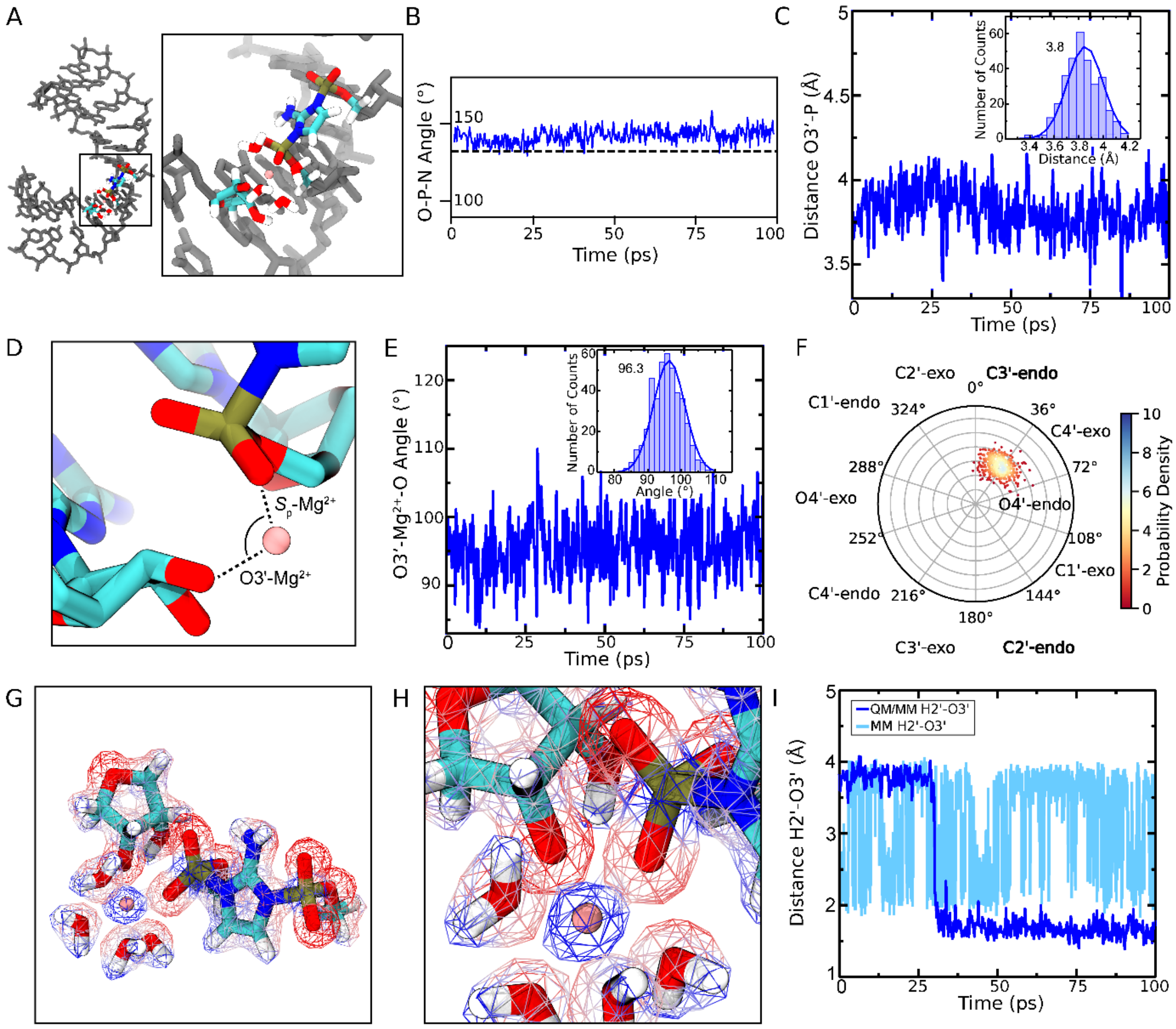

J

System	O3′-P Distance	Attack Angle	O3′-Mg^{2+}-O Angle	Primer Sugar Pucker
QM/MM	**3.8 Å**	**142.6°**	**96.3°**	**C3′-endo/C4′-exo**
Classical	**3.7 Å**	**148°**	**96°**	**C3′-endo**

***Figure S3*. QM/MM simulations of the 3′O$^-$ w/ Mg^{2+}@S_P, 2-NH_2-Im:R_P system (Sim10).** (A) Molecular figure of the simulation system in licorice representation showing the portion of the duplex modeled in MM (grey) and the 49 atoms modeled using QM (colored by atom type). (B) The angle of attack during the QM/MM simulation, measured between O3′-P-N atoms, which agrees well with the CHARMM simulations (Figure 5B). (C) Time series of the O3′ (primer)-P (bridged dinucleotide) distance during the QM/MM simulation. The inset shows a probability distribution with the mean value indicated, obtained from a normal fit. This distance agrees well with the average distance from the CHARMM simulations (Figure 4D). (D) Molecular figure showing the O3′-Mg^{2+}-O(S_p) angle measured during the QM/MM simulation (E) Time course of the angle measured in (D). The inset shows a probability distribution with the mean value indicated, obtained from a normal fit. This angle agrees well with the average angle from the CHARMM simulations (Figure S16). (F) Circular histogram of pseudorotation angles of the terminal primer nucleotide sugar during the QM/MM simulation. The phase angles are based on the Altona-Sundaralingam[1] definition and are assigned to the puckering modes in multiples of 36°. (G) A molecular figure of the QM system with calculated SCF electron density colored by the electrostatic potential with positive charge in blue and negative charge in red. (H) A zoomed in view of (G), showing the polarization on the H2′ and O3′. (I) Time series of the H2′ and O3′ distance for the MM and QM/MM simulations, showing the tighter interaction in QM/MM due to polarization of the H2′. The time indicated corresponds to the QM/MM simulations, while the trace corresponding to the MM simulation goes to 3 ns. (J) Summary of measured parameters.

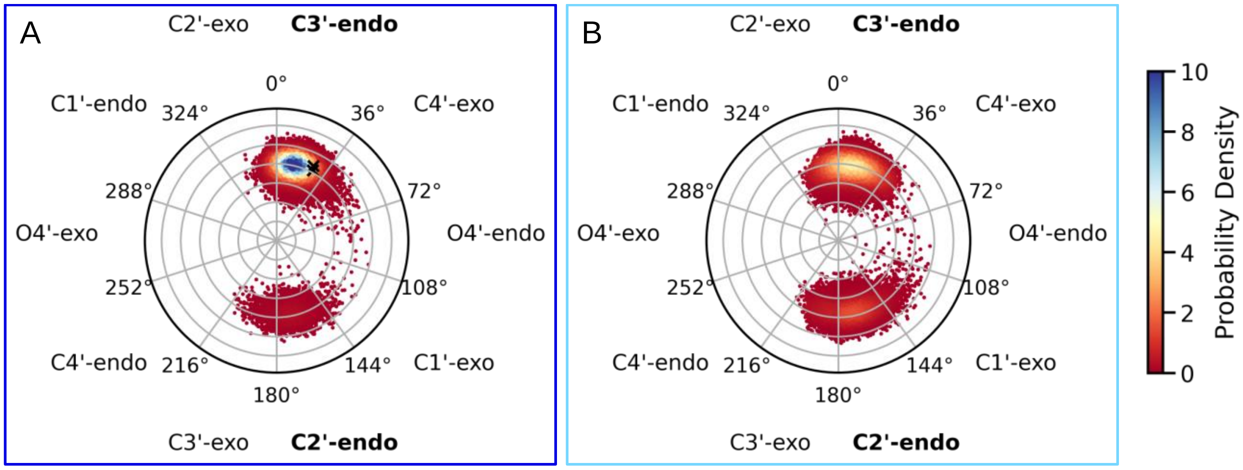

Figure S4. **Terminal primer sugar puckering in the absence of bound Mg^{2+} and a major groove-facing 2AI.** Circular histogram of pseudorotation angles and amplitudes of the terminal primer nucleotide sugar for the (A) 3′-OH w/o Mg^{2+} (Sim1) and (B) 3′O^- w/o Mg^{2+} (Sim2) simulation systems. The phase angles are based on the Altona-Sundaralingam[1] definition and are assigned to the puckering modes in multiples of 36°. Sugar pucker conformations of the primer nucleotide from the crystal structure, denoted with × markers in the circular plot show the sugars are in the C3′-endo conformation. Configurations that were more likely to be occupied are colored in blue, while less probable configurations are colored red.

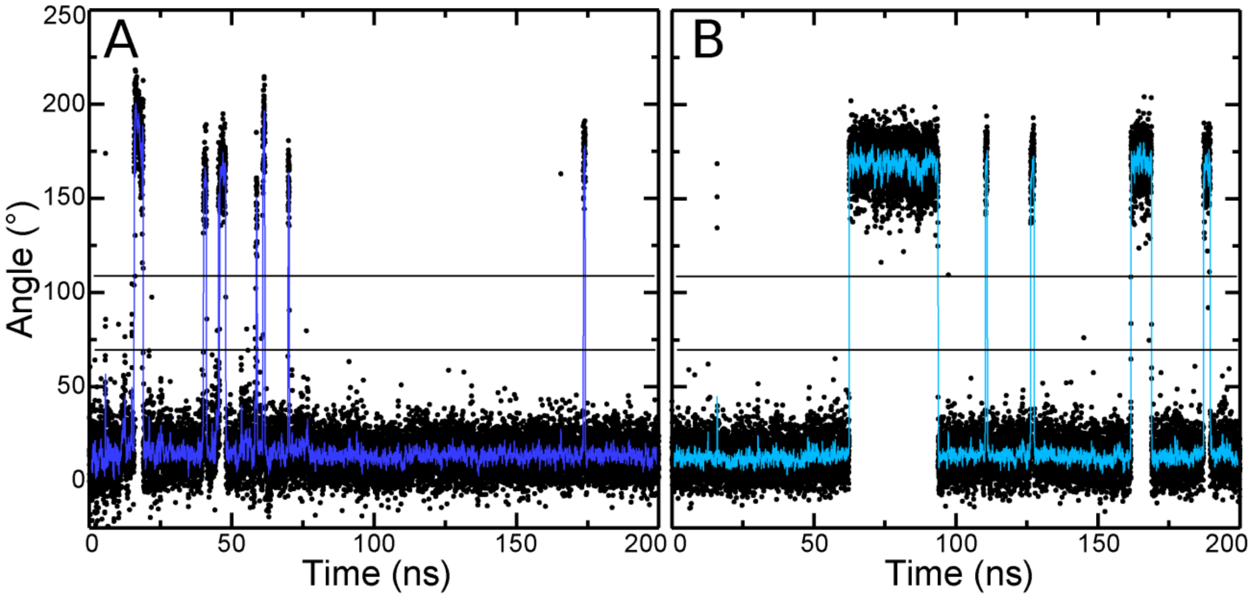

Figure S5. Time course of the terminal primer sugar pucker phase angle. (A) Phase angle of the terminal primer sugar for Sim1a. Individual data points shown as black circles, with a 0.15 ns running average. Horizontal lines show the range of angles corresponding to the O4'-endo conformation (72˚-108˚). (B) Phase angle of the terminal primer sugar for Sim2a. Individual data points shown as black circles, with a 0.15 ns running average. Horizontal lines show the range of angles corresponding to the O4'-endo conformation (72˚-108˚). For some transitions from C3'-endo to C2'-endo in both systems, data points between 72˚-108˚ indicate a transition through the O4'-endo conformation.

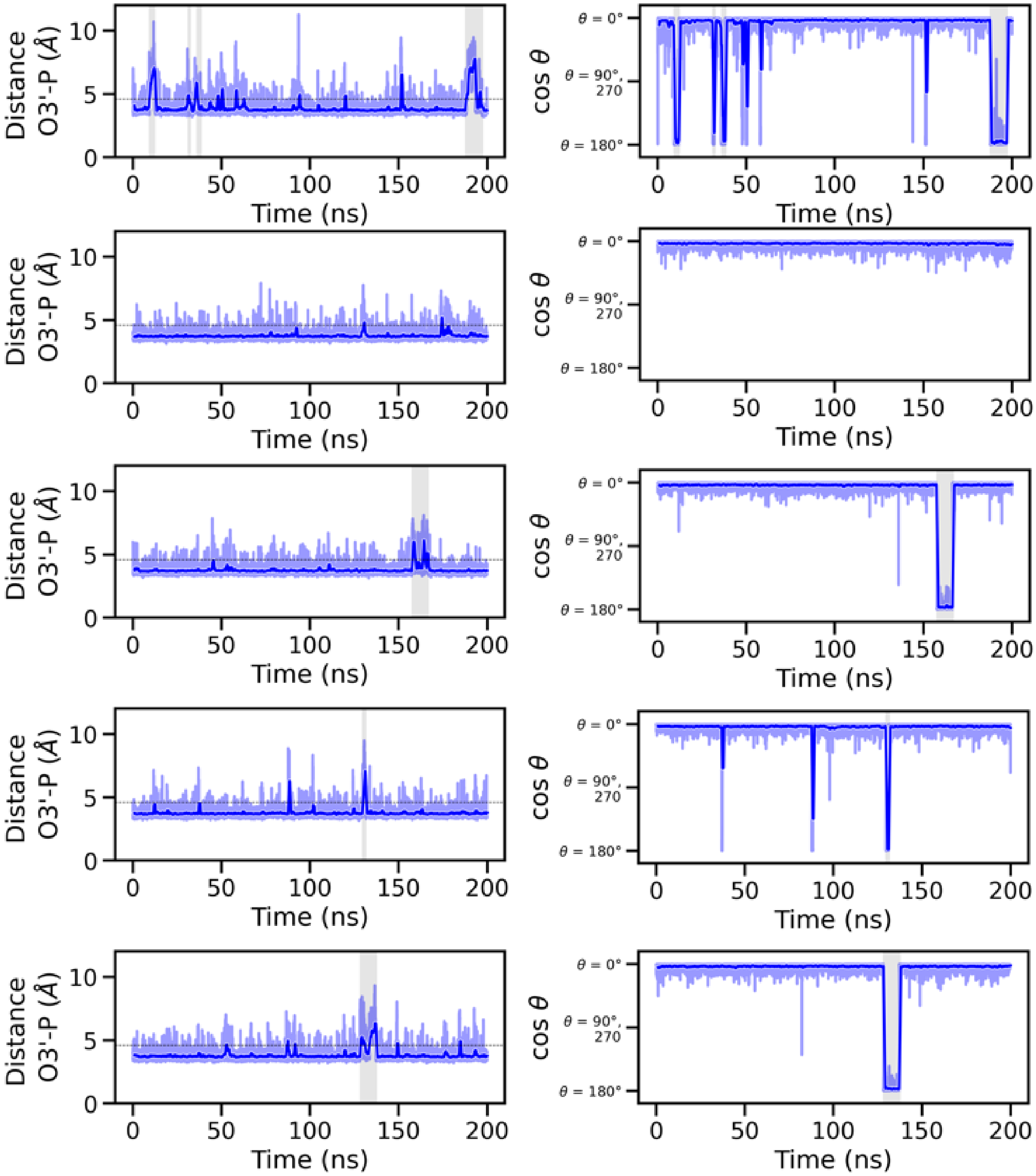

Figure S6. Time series of the distance O3′ (primer)-P (bridged dinucleotide) and primer nucleotide sugar pucker angle for the major groove-facing 3′-OH w/o Mg^{2+} simulation system (Sim1) for all five replicates. The horizontal dashed lines indicate the crystal structure value. Intervals with larger distance values and C2′-endo sugar conformations are highlighted in gray. For all time series plots, dark traces show the data averaged over a 1 ns window, while the lighter envelope shows the full range of the data recorded at 10 ps time steps in our simulations.

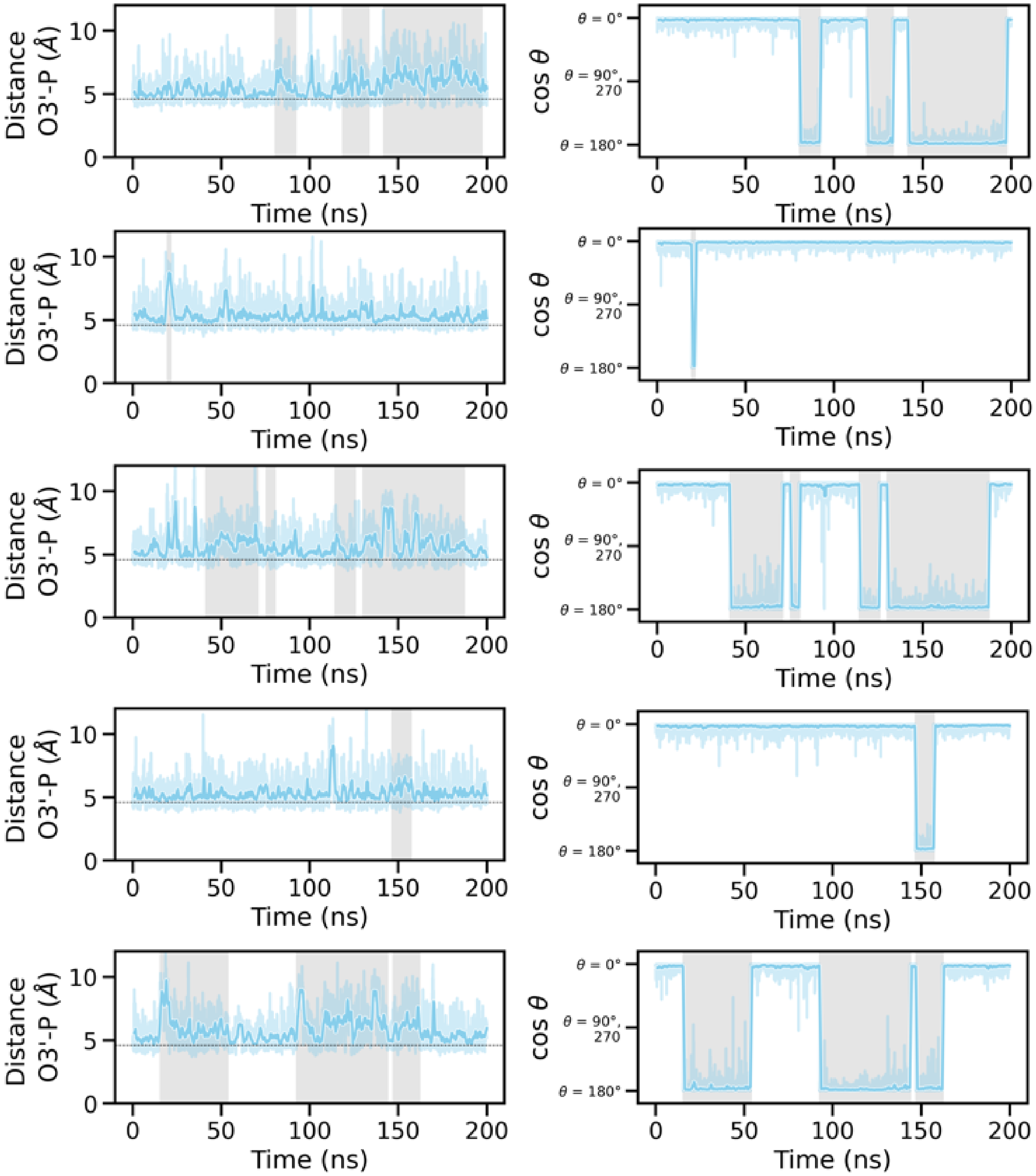

Figure S7. Time series of the distance O3′ (primer)-P (bridged dinucleotide) and primer nucleotide sugar pucker angle for the major groove-facing 3′O- w/o Mg^{2+} simulation system (Sim2) for all five replicates. The horizontal dashed lines indicate the crystal structure value. Intervals with larger distance values and C2′-endo sugar conformations are highlighted in gray. For all time series plots, dark traces show the data averaged over a 1 ns window, while the lighter envelope shows the full range of the data recorded at 10 ps time steps in our simulations.

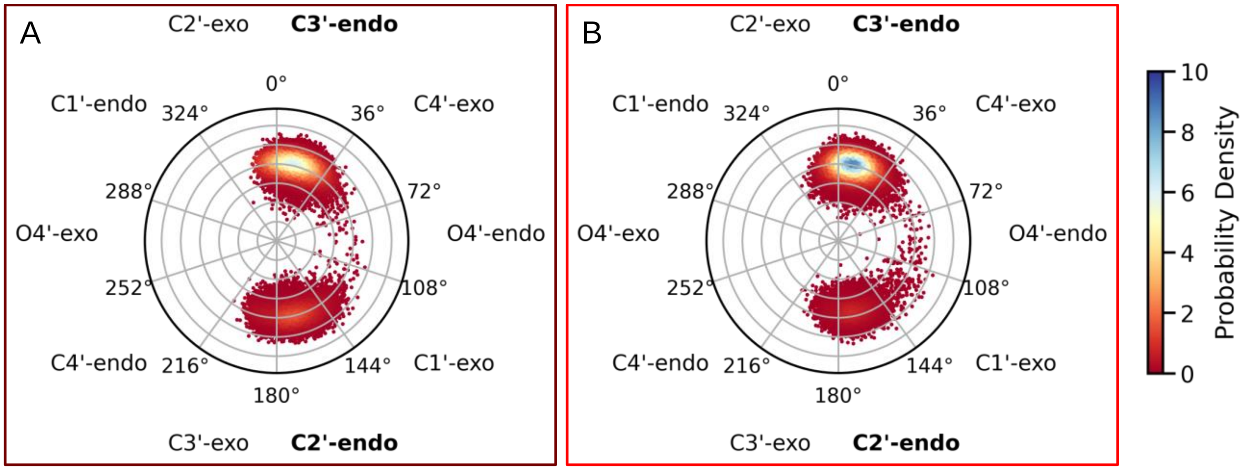

Figure S8. **Terminal primer sugar puckering in the absence of bound Mg^{2+} and a minor groove facing 2AI.** Circular histogram of pseudorotation angles of the terminal primer nucleotide sugar for the (A) 3′-OH w/o Mg^{2+} (Sim3) and (B) 3′O⁻ w/o Mg^{2+} (Sim4) simulation systems where 2-NH_2-Im orientation is minor groove-facing. The phase angles are based on the Altona-Sundaralingam[1] definition and are assigned to the puckering modes in multiples of 36°. Configurations that were more likely to be occupied are colored in blue, while less probable configurations are colored red.

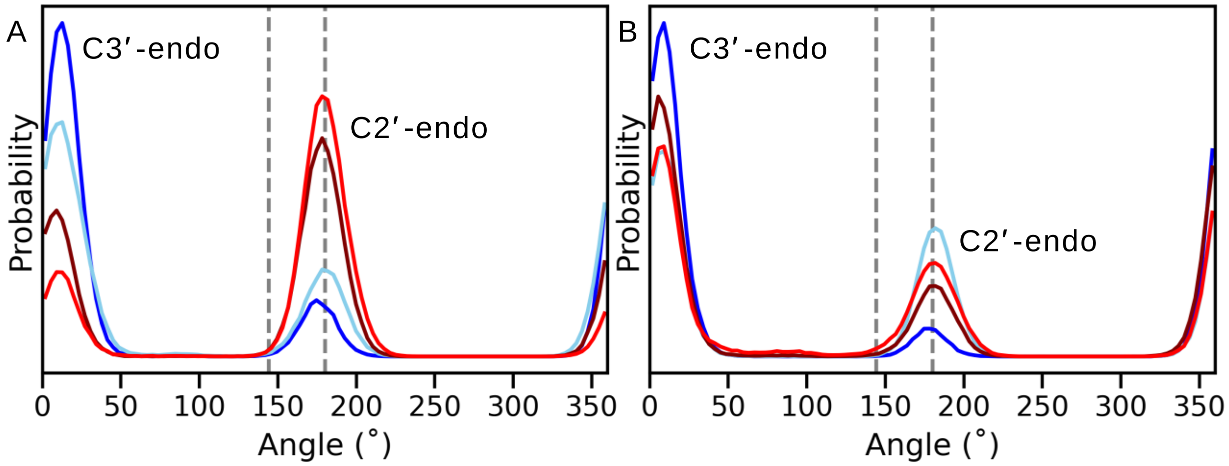

Figure S9. **Probability distribution of the pseudorotation angle of the bridged dinucleotide sugars in the absence of bound Mg^{2+}.** (A) G1 and (B) G2 positions in the major groove-facing conformations are shown for 3′-OH w/o Mg^{2+} (Sim1; dark blue) and 3′O- w/o Mg^{2+} (Sim2; light blue) and minor groove-facing conformations for 3′-OH w/o Mg^{2+} (Sim3; brown) and 3′O- w/o Mg^{2+} (Sim4; red). Dashed lines mark the region for C2′-endo sugar pucker.

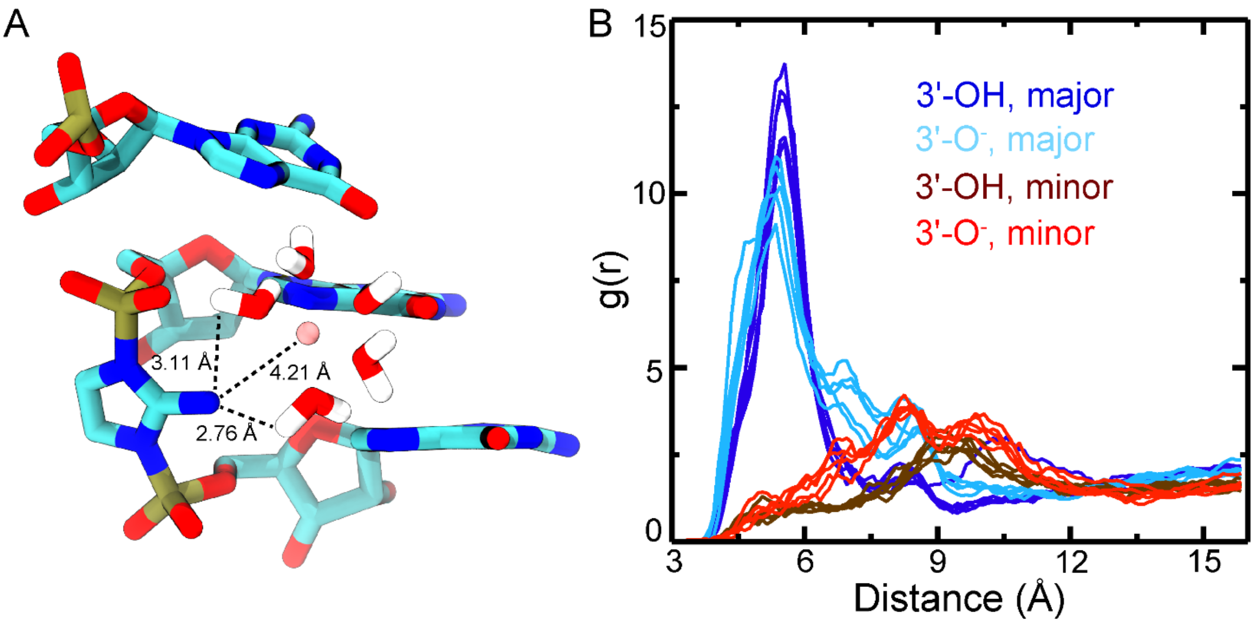

Figure S10. **Water mediated contacts between the bridging imidazolium and Mg^{2+}.** (A) Simulation snapshot (Sim1a) showing distances of Mg^{2+} ion and coordinating water molecules from the 2-NH_2-Im atom of the bridged nucleotide. (B) Radial distribution function of the distance between 2-NH_2-Im and Mg^{2+} ions in the MD simulation box for each simulation replicate.

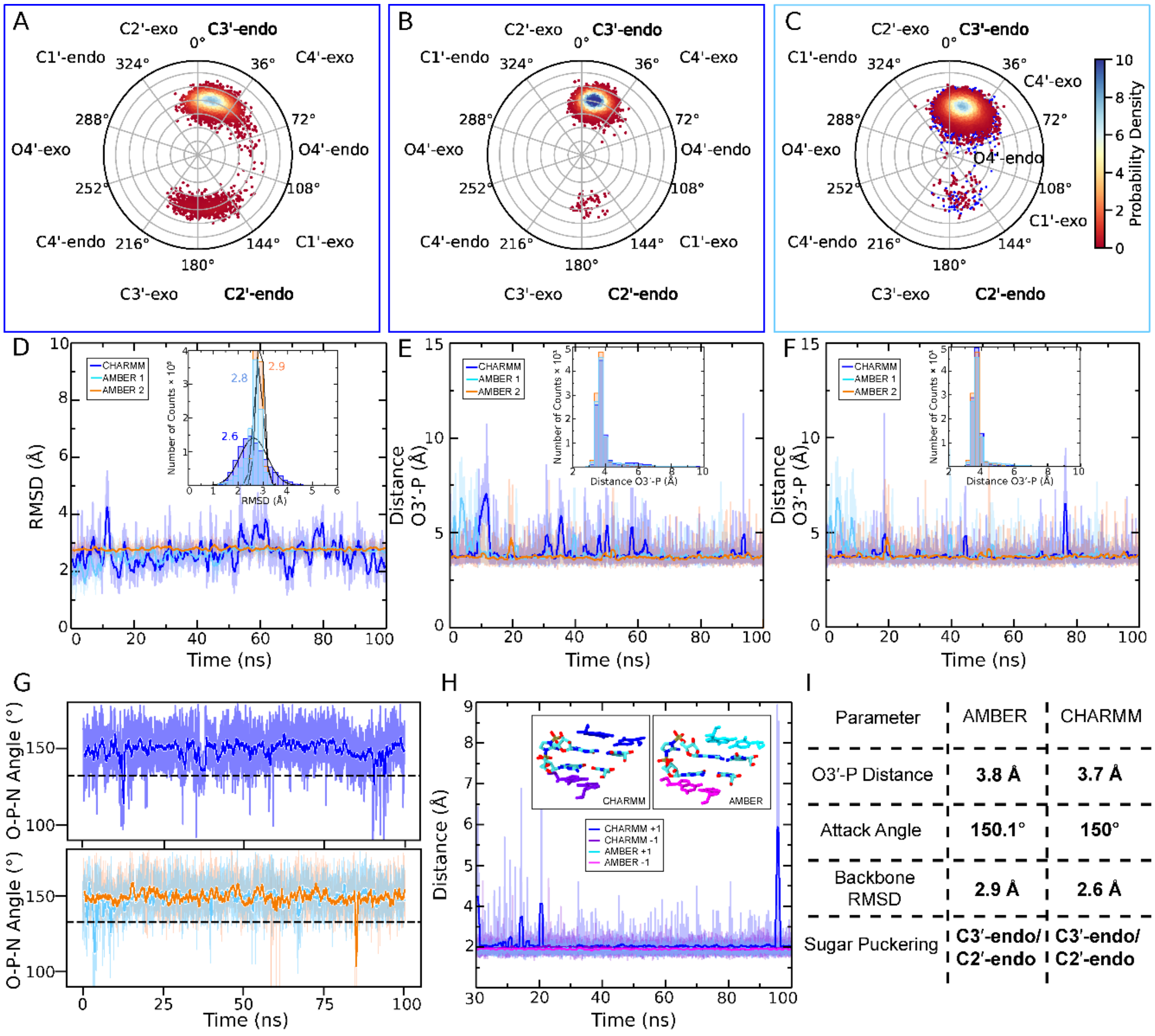

Parameter	AMBER	CHARMM
O3′-P Distance	**3.8 Å**	**3.7 Å**
Attack Angle	**150.1°**	**150°**
Backbone RMSD	**2.9 Å**	**2.6 Å**
Sugar Puckering	**C3′-endo/ C2′-endo**	**C3′-endo/ C2′-endo**

Figure S11. **Comparison of CHARMM (Sim1) and AMBER (Sim9) 3′-OH w/o Mg^{2+} simulations where 2-NH_2-Im orientation is major groove-facing.** Terminal primer sugar puckering for (A) 0-100 ns CHARMM, (B) 75-175 ns CHARMM, and (C) AMBER simulation systems. In (C), data points from the first 100 ns are shown on top, while data points from the second 100 ns are shown underneath in blue. The phase angles are based on the Altona-Sundaralingam[1] definition and are assigned to the puckering modes in multiples of 36°. (D) RMSD values (calculated for nucleic acid and bridged dinucleotide heavy atoms, see methods) of the 0-100 ns CHARMM and two 100 ns AMBER sub-trajectories. The inset shows a probability distribution for each, with mean values indicated in the corresponding color, obtained from a normal fit. Time series of the O3′ (primer)-P (bridged dinucleotide) distance for the 0-100 ns CHARMM and two 100 ns AMBER sub-trajectories (E), and the 75-175 ns CHARMM and two 100 ns AMBER sub-trajectories (F). The insets show a probability distribution for each. (G) The angle of attack during the 0-100 ns CHARMM (top) and two 100 ns AMBER (bottom) sub-trajectories, measured between O3′-P-N atoms. (H) Time series of the distances d(N1,N3) between the G-C base pairs flanking the bridged dinucleotide in the 0-100 ns CHARMM and AMBER simulations. The inset indicates which color traces correspond to the upstream and downstream positions for each simulation. (I) Summary of measured parameters comparing between the AMBER and CHARMM systems.

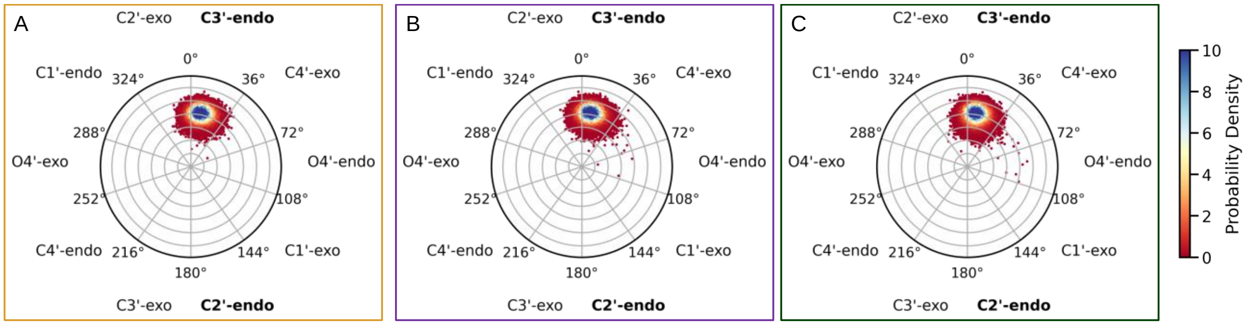

Figure S12. **Terminal primer sugar puckering with bound Mg^{2+}.** Circular histogram of pseudorotation angles of the terminal primer nucleotide sugar for the (A) 3′O- w/ Mg^{2+}@S_P, 2-NH_2-Im:R_P (Sim5; yellow), (B) 3′O- w/ Mg^{2+}@R_P, 2-NH_2-Im:R_P (Sim6; purple), and (C) 3′O- w/ Mg^{2+}@R_P, 2-NH_2-Im:S_P (Sim7; green) simulation systems. The phase angles are based on the Altona-Sundaralingam[1] definition and are assigned to the puckering modes in multiples of 36°.

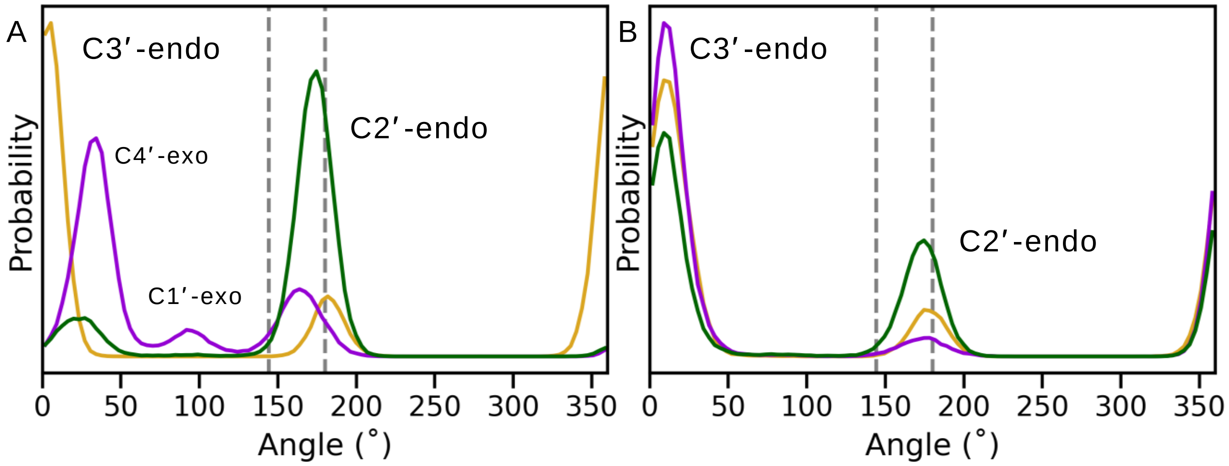

Figure S13. **Probability distribution of the pseudorotation angle of the bridged dinucleotide sugars with bound Mg^{2+}.** Probability distribution of the pseudorotation angle of the bridged dinucleotide sugars in the (A) G1 and (B) G2 positions for the simulation ensembles with Mg^{2+} in the reaction center: 3′O- w/ Mg^{2+}@S_P, 2-NH_2-Im:R_P (Sim5; yellow), 3′O- w/ Mg^{2+}@R_P, 2-NH_2-Im:R_P (Sim6; purple) and 3′O- w/ Mg^{2+}@R_P, 2-NH_2-Im:S_P (Sim7; green). Dashed lines mark the region for C2′-endo sugar pucker.

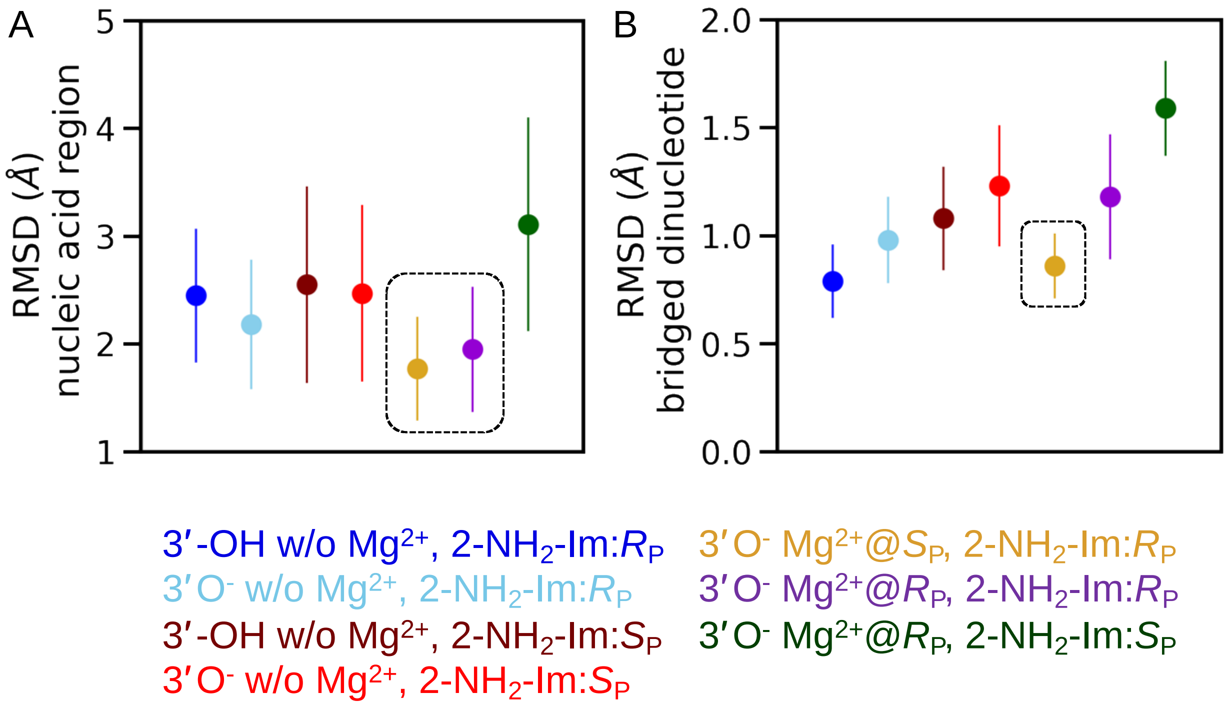

Figure S14. **A bridging Mg^{2+} in the reaction center stabilizes the primer extension complex.** Comparing mean ± standard deviation RMSD of (A) nucleic acid region and (B) bridged dinucleotide heavy atoms among the seven simulation ensembles discussed in this work. Systems highlighted in the main text are outlined.

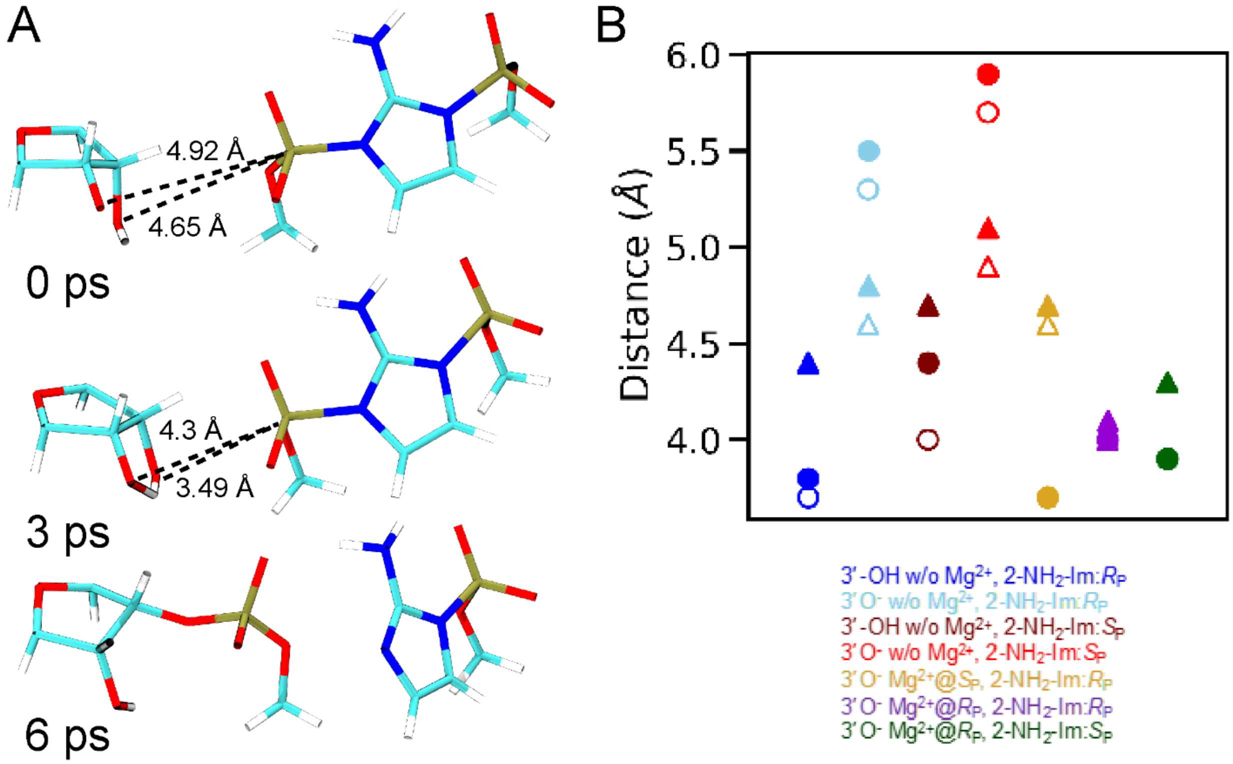

Figure S15. **Mg^{2+} increases the likelihood of forming the correct 3'-5' linkage.** (A) Reaction observed between the 2'-oxygen and reactive phosphate during Sim11. Quantum region shown in active bonds representation, which determines covalent bonds based on distance between atoms. (B) Distance between O2′-P (△ or ▲) and O3′-P (○ or ●) for the seven simulation ensembles discussed in this work. Open and closed markers show mean and median values respectively. Light blue and red highlighted simulation systems show a reversed trend where the average distance(O2′-P) < distance(O3′-P) whereas distance(O2′-P) ≈ distance(O3′-P) for the purple highlighted simulation system.

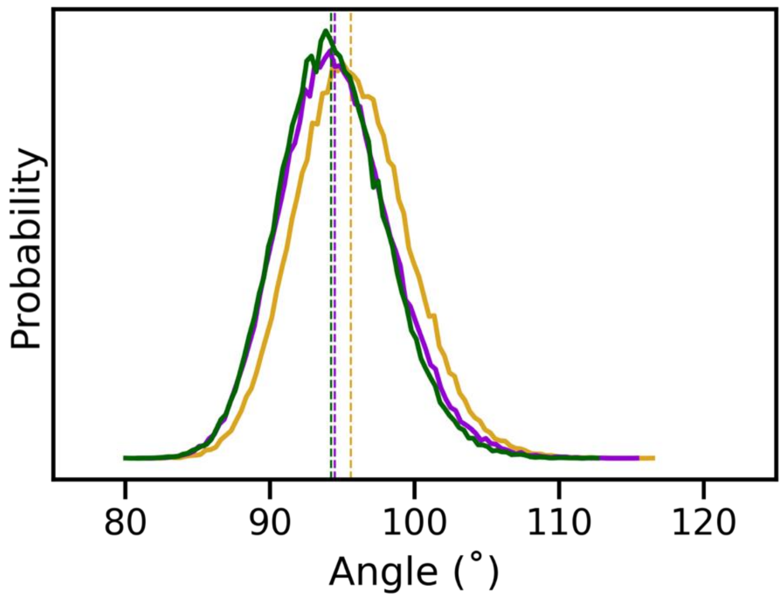

Figure S16. **Probability distribution of the Mg^{2+} coordination angle.** Angles measured between the O3′-Mg^{2+}-O (R_P or S_P) atoms in three simulation ensembles: 3′O^- w/ Mg^{2+}@S_P, 2-NH_2-Im:R_P (Sim5; yellow), 3′O^- w/ Mg^{2+}@R_P, 2-NH_2-Im:R_P (Sim6; purple) and 3′O^- w/ Mg^{2+}@R_P, 2-NH_2-Im:S_P (Sim7; green). Dashed lines show median values for the distributions.

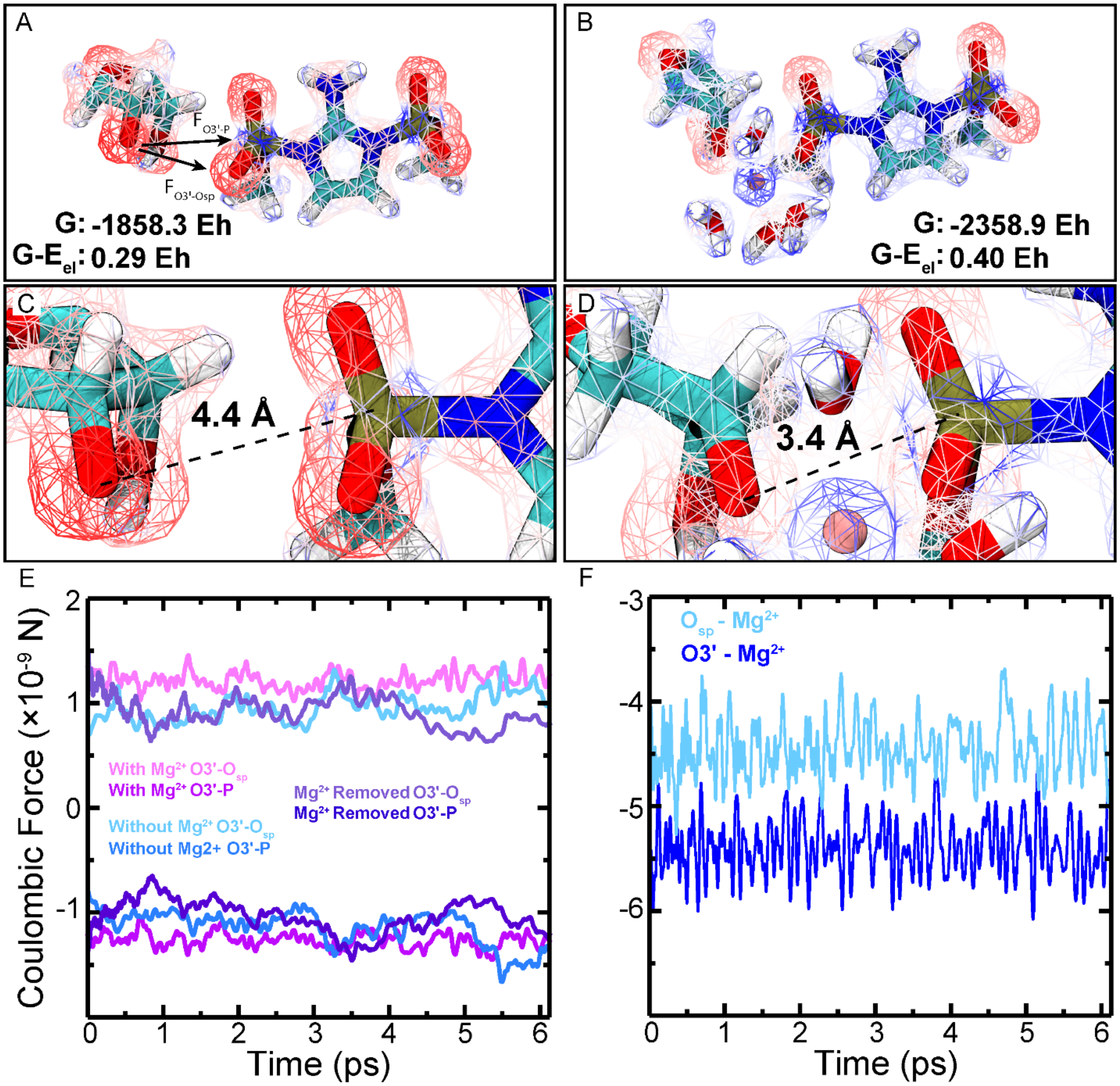

Figure S17. The presence of Mg^{2+} reduces electrostatic repulsion in the reaction center complex. A molecular figure of the QM system with calculated SCF electron density colored by the electrostatic potential with positive charge in blue and negative charge in red for the system without (A; Sim10) and with (B; Sim11) Mg^{2+} present, visualized at the same isovalue. Total Gibbs free energy (G) and Gibbs free energy minus electronic energy ($G-E_{el}$) are shown calculated using an HF-3c method with an implicit solvent model for each system. (C) Zoomed in view of (A), showing the O3'-P distance at the end of the simulation. (D) Zoomed in view of (B), showing the O3'-P distance at the end of the simulation, and a quantitatively reduced potential compared to (C) suggested by the lighter color of the electrostatic map. (E) Coulombic force calculated between the indicated atoms for the system without Mg^{2+} (Sim10), with Mg^{2+} (Sim11), and the system where Mg^{2+} was removed (Sim12). (F) Coulombic force calculated between the indicated atoms for the system with Mg^{2+} (Sim11), showing that in addition to the electrostatic influences demonstrated in (E), the presence of Mg^{2+} also exerts significant attractive forces between the relevant O atoms in the reactive center.

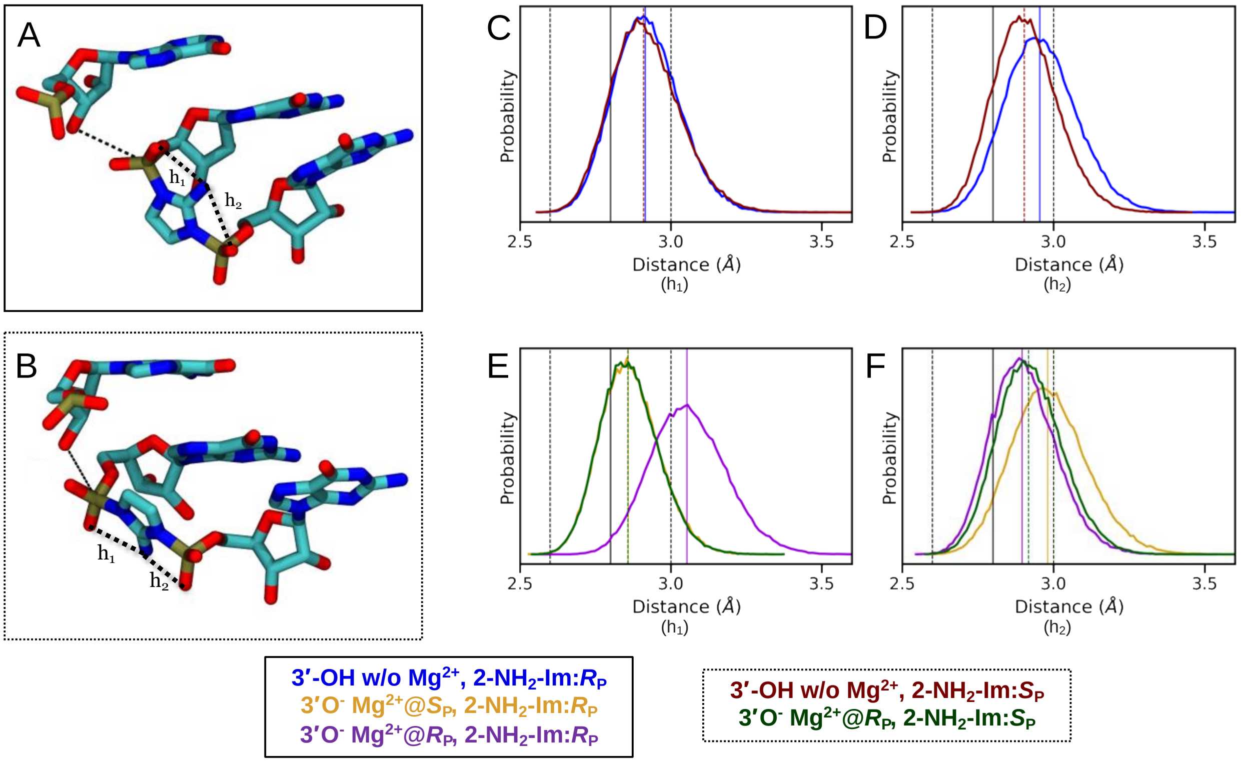

Figure S18. **Hydrogen bonds h_1 and h_2 between 2-NH_2-Im and non-bridging oxygens on the bridged dinucleotide suggest a preferred preorganization geometry.** (A) The major groove-facing and (B) minor groove-facing orientation of the bridged dinucleotide are shown. (C-F) Probability distribution of the hydrogen bond distances h_1 and h_2 for the various simulation systems. Median distance values from simulation with major and minor groove-facing orientations of bridged dinucleotide are shown in solid and dashed lines, respectively. Lines in black show hydrogen bond distances observed in the crystal structure.

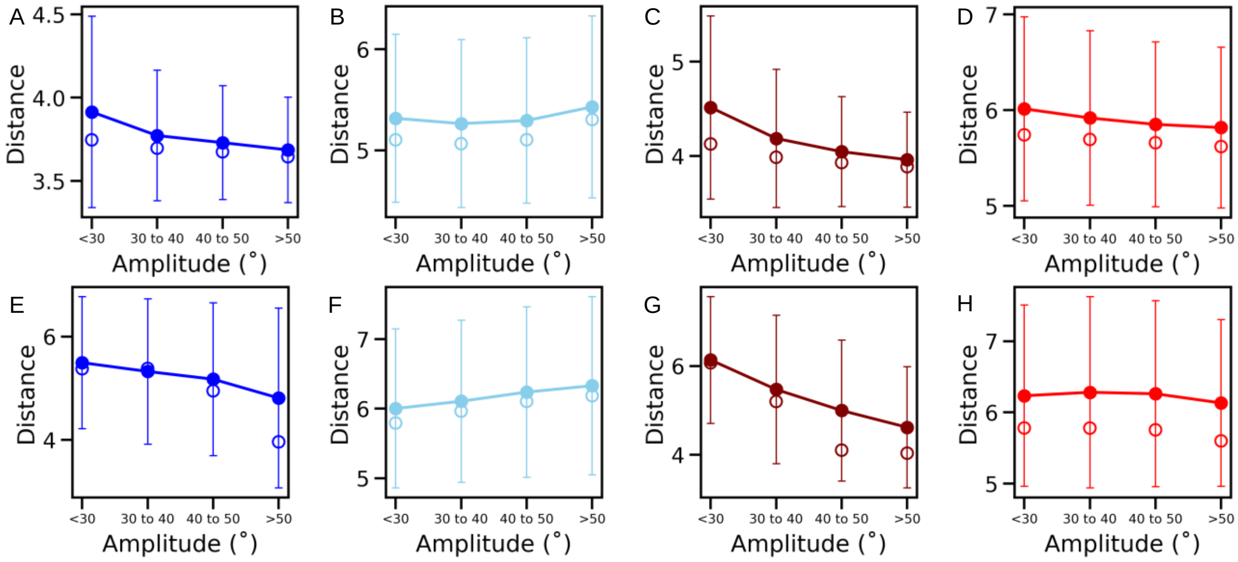

Figure S19. **O3′-P distance as a function of puckering amplitude without bound Mg^{2+}.** (A-D) Mean (●) and median (○) of the O3′-P distance distributions when the terminal primer sugar is in the C3′-endo conformation with amplitudes <30°, 30-40°, 40-50°, and >50°. (E-H) Mean (●) and median (○) of the O3′-P distance distributions when the terminal primer sugar is in the C2′-endo conformation with amplitudes <30°, 30-40°, 40-50°, and >50°. Simulation ensembles include major groove-facing 3′-OH (dark blue) and 3′O^- (light blue), and minor groove-facing 3′-OH (brown) and 3′O^- (red). Standard deviation is shown as colored bars.

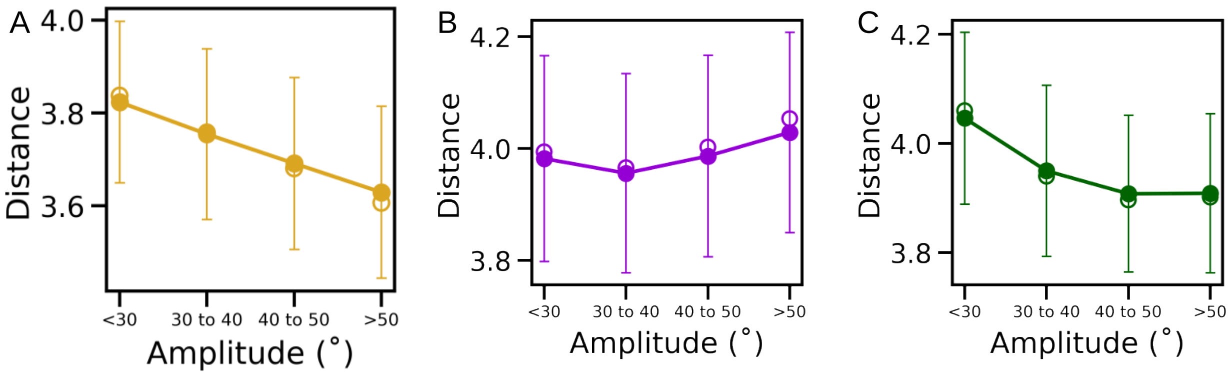

***Figure S20.* O3′-P distance as a function of puckering amplitude with bound Mg^{2+}.** Mean (●) and median (○) of the O3′-P distance distributions when the terminal primer sugar is in the C3′-endo conformation with amplitudes <30°, 30-40°, 40-50°, and >50°. Simulation ensembles include (A) 3′O^- w/ Mg^{2+}@S_P, 2-NH_2-Im:R_P (Sim5; yellow), (B) 3′O^- w/ Mg^{2+}@R_P, 2-NH_2-Im:R_P (Sim6; purple) and (C) 3′O^- w/ Mg^{2+}@R_P, 2-NH_2-Im:S_P (Sim7; green). Simulation frames in C2′-endo are not observed. Standard deviation is shown as colored bars.

TABLE S1 2AI-Bridged Dinucleotide Parameters

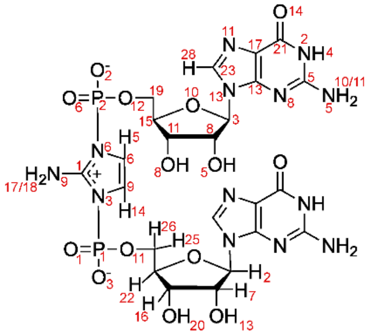

Atom Name	Atom Type	Charge
ATOM P1	PG1	1.599
ATOM P2	PG1	1.599
ATOM C1	CG2R53	0.483
ATOM C2	CG3C51	0.146
ATOM C3	CG3C51	0.146
ATOM N3	NG2R52	-0.557
ATOM O1	OG2P1	-0.821
ATOM O2	OG2P1	-0.821
ATOM C6	CG2R51	0.171
ATOM O6	OG2P1	-0.821
ATOM O12	OG303	-0.534
ATOM N6	NG2R52	-0.557
ATOM H5	HGR52	0.130
ATOM C1	CG2R53	0.483
ATOM N9	NG2P1	-0.621
ATOM H17	HGP2	0.329
ATOM H18	HGP2	0.329
ATOM O11	OG303	-0.534
ATOM O3	OG2P1	-0.821

Atom Name	Atom Type	Charge
ATOM C19	CG321	-0.101
ATOM C15	CG3C51	0.135
ATOM C11	CG3C51	0.145
ATOM O8	OG311	-0.650
ATOM C11	CG3C51	0.145
ATOM C8	CG3C51	0.143
ATOM C3	CG3C51	0.146
ATOM O10	OG3C51	-0.468
ATOM O8	OG311	-0.650
ATOM O5	OG311	-0.650
ATOM N13	NG2R51	-0.017
ATOM C23	CG2R53	0.251
ATOM N11	NG2R50	-0.595
ATOM C17	CG2RC0	0.004
ATOM C13	CG2RC0	0.264
ATOM N8	NG2R62	-0.738
ATOM C5	CG2R64	0.730
ATOM N5	NG2S3	-0.663
ATOM N2	NG2R61	-0.340

Atom Name	Atom Type	Charge
ATOM C21	CG2R63	0.544
ATOM O14	OG2D4	-0.509
ATOM H25	HGA2	0.090
ATOM H26	HGA2	0.090
ATOM H22	HGA1	0.090
ATOM H16	HGA1	0.090
ATOM H20	HGP1	0.419
ATOM H13	HGP1	0.419
ATOM H7	HGA1	0.090
ATOM H2	HGA1	0.090
ATOM H28	HGR52	0.143
ATOM H10	HGP4	0.327
ATOM H11	HGP4	0.327
ATOM H4	HGP1	0.267

Bond	Force Constant	Equilibrium Distance
CG2R51 CG2R51	410.00	1.3600
CG2R51 NG2R52	380.00	1.3700
CG2R51 HGR52	375.00	1.0830
CG2R53 NG2P1	300.00	1.3820
CG2R53 NG2R50	400.00	1.3200
CG2R53 NG2R51	320.00	1.3740
CG2R53 NG2R52	380.00	1.3200
CG2R53 HGR52	340.00	1.0900
CG2R63 CG2RC0	302.00	1.3600
CG2R63 NG2R61	340.00	1.3830
CG2R63 OG2D4	660.00	1.2340
CG2R64 NG2R61	400.00	1.3920
CG2R64 NG2R62	400.00	1.3420
CG2R64 NG2S3	360.00	1.3660
CG2RC0 CG2RC0	360.00	1.3850
CG2RC0 NG2R50	310.00	1.3650
CG2RC0 NG2R51	300.00	1.3750
CG2RC0 NG2R62	350.00	1.3150
CG321 CG3C51	222.50	1.5280
CG321 OG303	320.00	1.4400
CG321 HGA2	309.00	1.1110
CG3C51 CG3C51	195.00	1.5180
CG3C51 NG2R51	220.00	1.4580
CG3C51 OG311	428.00	1.4200
CG3C51 OG3C51	350.00	1.4250
CG3C51 HGA1	307.00	1.1000
NG2P1 HGP2	455.00	1.0000
NG2R52 PG1	180.00	1.7920
NG2R61 HGP1	474.00	1.0100
NG2S3 HGP4	488.00	1.0000
OG2P1 PG1	500.00	1.5100
OG303 PG1	190.00	1.6500
OG311 HGP1	545.00	0.9600

Angle	Force Constant	Equilibrium Angle
CG2R51 CG2R51 NG2R52	145.00	108.00
NG2P1 CG2R53 NG2R52	65.00	127.80
NG2R50 CG2R53 NG2R51	100.00	113.00
NG2R50 CG2R53 HGR52	39.00	124.80
NG2R51 CG2R53 HGR52	40.00	122.20
NG2R52 CG2R53 NG2R52	145.00	108.00
CG2RC0 CG2R63 NG2R61	70.00	107.80
CG2RC0 CG2R63 OG2D4	50.00	124.70
NG2R61 CG2R63 OG2D4	130.00	119.40
NG2R61 CG2R64 NG2R62	70.00	122.20
NG2R61 CG2R64 NG2S3	95.00	115.40
NG2R62 CG2R64 NG2S3	95.00	122.40
CG2R63 CG2RC0 CG2RC0	70.00	119.60
CG2R63 CG2RC0 NG2R50	125.00	129.00
CG2RC0 CG2RC0 NG2R50	100.00	110.00
CG2RC0 CG2RC0 NG2R51	100.00	105.70
CG2RC0 CG2RC0 NG2R62	60.00	127.40
NG2R51 CG2RC0 NG2R62	100.00	126.90
CG3C51 CG321 OG303	75.70	110.10
OG303 CG321 HGA2	60.00	110.10
CG321 CG3C51 OG3C51	45.00	111.50
CG3C51 CG3C51 NG2R51	110.00	111.00
CG3C51 CG3C51 OG311	75.70	110.10
CG3C51 CG3C51 OG3C51	45.00	111.10
CG3C51 CG3C51 HGA1	35.00	111.40
NG2R51 CG3C51 OG3C51	140.00	108.00
NG2R51 CG3C51 HGA1	43.00	111.00
OG311 CG3C51 HGA1	45.90	108.89
OG3C51 CG3C51 HGA1	70.00	107.30
CG2R53 NG2P1 HGP2	47.00	120.00
HGP2 NG2P1 HGP2	25.00	120.00
CG2R53 NG2R50 CG2RC0	120.00	103.80
CG2R53 NG2R51 CG2RC0	100.00	107.20

Angle	Force Constant	Equilibrium Angle
CG2R53 NG2R51 CG3C51	45.00	126.30
CG2RC0 NG2R51 CG3C51	45.00	126.50
CG2R51 NG2R52 CG2R53	145.00	108.00
CG2R63 NG2R61 CG2R64	70.00	131.10
CG2R63 NG2R61 HGP1	40.50	115.40
CG2R64 NG2R61 HGP1	45.00	115.60
CG2R64 NG2R62 CG2RC0	90.00	115.10
CG2R64 NG2S3 HGP4	40.00	121.50
HGP4 NG2S3 HGP4	31.00	117.00
CG3C51 OG311 HGP1	50.00	109.00
CG3C51 OG3C51 CG3C51	95.00	111.00
NG2R52 PG1 OG2P1	140.00	110.60
NG2R52 PG1 OG303	60.00	103.20
OG2P1 PG1 OG2P1	104.00	120.00
OG2P1 PG1 OG303	98.90	107.50

Angle	Force Cons.	Eq. Angle	K_{UB}	S_0
CG2R51 CG2R51 HGR52	22.00	130.00	15.00	2.2150
NG2R52 CG2R51 HGR52	22.00	122.00	15.00	2.1800
CG3C51 CG321 HGA2	34.60	110.10	22.53	2.1790
HGA2 CG321 HGA2	35.50	109.00	5.40	1.8020
CG321 CG3C51 CG3C51	58.00	115.00	8.00	2.5610
CG321 CG3C51 HGA1	34.60	110.10	22.53	2.1790
CG3C51 CG3C51 CG3C51	58.00	109.50	11.16	2.5610
CG2R51 NG2R52 PG1	44.00	126.30	23.00	2.4300
CG2R53 NG2R52 PG1	44.00	126.30	23.00	2.4300
CG321 OG303 PG1	20.00	120.00	35.00	2.3300

Improper			
CG2R53 NG2R52 NG2R52 NG2P1	90.0000	0	0.00
CG2R63 CG2RC0 NG2R61 OG2D4	90.0000	0	0.00
CG2R64 NG2R61 NG2R62 NG2S3	40.0000	0	0.00
NG2S3 HGP4 HGP4 CG2R64	9.0000	0	0.00

Dihedral	Force Cons.	Mult.	Phase
NG2R52 CG2R51 CG2R51 NG2R52	12.0000	2	180.00
NG2R52 CG2R51 CG2R51 HGR52	2.5000	2	180.00
HGR52 CG2R51 CG2R51 HGR52	1.0000	2	180.00
CG2R51 CG2R51 NG2R52 CG2R53	12.0000	2	180.00
CG2R51 CG2R51 NG2R52 PG1	6.0000	2	180.00
HGR52 CG2R51 NG2R52 CG2R53	2.5000	2	180.00
HGR52 CG2R51 NG2R52 PG1	9.0000	2	180.00
NG2R52 CG2R53 NG2P1 HGP2	2.4000	2	180.00
NG2R51 CG2R53 NG2R50 CG2RC0	14.0000	2	180.00
HGR52 CG2R53 NG2R50 CG2RC0	5.2000	2	180.00
NG2R50 CG2R53 NG2R51 CG2RC0	6.0000	2	180.00
NG2R50 CG2R53 NG2R51 CG3C51	11.0000	2	180.00
HGR52 CG2R53 NG2R51 CG2RC0	5.6000	2	180.00
HGR52 CG2R53 NG2R51 CG3C51	0.0000	2	180.00
NG2P1 CG2R53 NG2R52 CG2R51	12.0000	2	180.00
NG2P1 CG2R53 NG2R52 PG1	3.7000	2	180.00
NG2R52 CG2R53 NG2R52 CG2R51	12.0000	2	180.00
NG2R52 CG2R53 NG2R52 PG1	3.7000	2	180.00
NG2R61 CG2R63 CG2RC0 CG2RC0	0.2000	2	180.00
NG2R61 CG2R63 CG2RC0 NG2R50	2.0000	2	180.00
OG2D4 CG2R63 CG2RC0 CG2RC0	14.0000	2	180.00
OG2D4 CG2R63 CG2RC0 NG2R50	0.0000	2	180.00
CG2RC0 CG2R63 NG2R61 CG2R64	0.2000	2	180.00
CG2RC0 CG2R63 NG2R61 HGP1	3.6000	2	180.00
OG2D4 CG2R63 NG2R61 CG2R64	14.0000	2	180.00
OG2D4 CG2R63 NG2R61 HGP1	0.0000	2	180.00
NG2R62 CG2R64 NG2R61 CG2R63	0.2000	2	180.00
NG2R62 CG2R64 NG2R61 HGP1	3.6000	2	180.00
NG2S3 CG2R64 NG2R61 CG2R63	4.0000	2	180.00
NG2S3 CG2R64 NG2R61 HGP1	0.0000	2	180.00
NG2R61 CG2R64 NG2R62 CG2RC0	2.0000	2	180.00
NG2S3 CG2R64 NG2R62 CG2RC0	4.0000	2	180.00
NG2R61 CG2R64 NG2S3 HGP4	1.2000	2	180.00
NG2R62 CG2R64 NG2S3 HGP4	1.0000	2	180.00
CG2R63 CG2RC0 CG2RC0 NG2R51	10.0000	2	180.00

Dihedral	Force Cons.	Mult.	Phase
CG2R63 CG2RC0 CG2RC0 NG2R62	2.0000	2	180.00
NG2R50 CG2RC0 CG2RC0 NG2R51	10.0000	2	180.00
NG2R50 CG2RC0 CG2RC0 NG2R62	7.0000	2	180.00
CG2R63 CG2RC0 NG2R50 CG2R53	2.0000	2	180.00
CG2RC0 CG2RC0 NG2R50 CG2R53	6.0000	2	180.00
CG2RC0 CG2RC0 NG2R51 CG2R53	6.0000	2	180.00
CG2RC0 CG2RC0 NG2R51 CG3C51	11.0000	2	180.00
NG2R62 CG2RC0 NG2R51 CG2R53	2.0000	2	180.00
NG2R62 CG2RC0 NG2R51 CG3C51	11.0000	2	180.00
CG2RC0 CG2RC0 NG2R62 CG2R64	0.2000	2	180.00
NG2R51 CG2RC0 NG2R62 CG2R64	2.0000	2	180.00
OG303 CG321 CG3C51 CG3C51	2.5000	1	180.00
OG303 CG321 CG3C51 CG3C51	0.4000	2	0.00
OG303 CG321 CG3C51 CG3C51	0.8000	3	180.00
OG303 CG321 CG3C51 CG3C51	0.2000	4	180.00
OG303 CG321 CG3C51 OG3C51	3.4000	1	180.00
OG303 CG321 CG3C51 HGA1	0.1950	3	0.00
HGA2 CG321 CG3C51 CG3C51	0.1600	3	0.00
HGA2 CG321 CG3C51 OG3C51	0.1600	3	0.00
HGA2 CG321 CG3C51 HGA1	0.1600	3	0.00
CG3C51 CG321 OG303 PG1	0.6000	1	180.00
CG3C51 CG321 OG303 PG1	0.6500	2	0.00
CG3C51 CG321 OG303 PG1	0.0500	3	0.00
HGA2 CG321 OG303 PG1	0.0000	3	0.00
CG321 CG3C51 CG3C51 CG3C51	0.1900	3	0.00
CG321 CG3C51 CG3C51 OG311	0.1400	3	0.00
CG321 CG3C51 CG3C51 HGA1	0.1900	3	0.00
CG3C51 CG3C51 CG3C51 CG3C51	0.4100	3	180.00
CG3C51 CG3C51 CG3C51 NG2R51	0.0000	3	0.00
CG3C51 CG3C51 CG3C51 OG311	0.2000	3	0.00
CG3C51 CG3C51 CG3C51 OG3C51	0.0000	3	0.00
CG3C51 CG3C51 CG3C51 HGA1	0.1900	3	0.00
NG2R51 CG3C51 CG3C51 OG311	0.0000	3	0.00
NG2R51 CG3C51 CG3C51 HGA1	0.0000	3	0.00
OG311 CG3C51 CG3C51 OG311	0.0000	3	0.00

Dihedral	Force Cons.	Mult.	Phase
OG311 CG3C51 CG3C51 OG3C51	0.2000	3	0.00
OG311 CG3C51 CG3C51 OG3C51	0.6000	4	180.0
OG311 CG3C51 CG3C51 OG3C51	0.3000	5	0.00
OG311 CG3C51 CG3C51 OG3C51	0.5000	6	0.00
OG311 CG3C51 CG3C51 HGA1	0.1950	3	0.00
OG3C51 CG3C51 CG3C51 HGA1	0.1900	3	0.00
HGA1 CG3C51 CG3C51 HGA1	0.1900	3	0.00
CG3C51 CG3C51 NG2R51 CG2R53	0.0000	3	180.0
CG3C51 CG3C51 NG2R51 CG2RC0	0.0000	3	0.00
OG3C51 CG3C51 NG2R51 CG2R53	1.1000	1	0.00
OG3C51 CG3C51 NG2R51 CG2RC0	1.1000	1	180.0
OG3C51 CG3C51 NG2R51 CG2RC0	0.2000	3	0.00
HGA1 CG3C51 NG2R51 CG2R53	0.2500	2	180.0
HGA1 CG3C51 NG2R51 CG2R53	0.1950	3	0.00
HGA1 CG3C51 NG2R51 CG2RC0	0.2500	2	180.0
CG3C51 CG3C51 OG311 HGP1	0.2900	1	0.00
CG3C51 CG3C51 OG311 HGP1	0.6200	2	0.00
CG3C51 CG3C51 OG311 HGP1	0.0500	3	0.00
HGA1 CG3C51 OG311 HGP1	0.1800	3	0.00
CG321 CG3C51 OG3C51 CG3C51	0.3000	3	0.00
CG3C51 CG3C51 OG3C51 CG3C51	0.5000	3	0.00
NG2R51 CG3C51 OG3C51 CG3C51	0.0000	3	0.00
HGA1 CG3C51 OG3C51 CG3C51	0.3000	3	0.00
CG2R51 NG2R52 PG1 OG2P1	0.4000	3	0.00
CG2R51 NG2R52 PG1 OG2P1	0.5000	4	0.00
CG2R51 NG2R52 PG1 OG303	1.9000	2	0.00
CG2R53 NG2R52 PG1 OG2P1	0.4000	3	0.00
CG2R53 NG2R52 PG1 OG2P1	0.5000	4	0.00
CG2R53 NG2R52 PG1 OG303	1.9000	2	0.00
CG321 OG303 PG1 NG2R52	0.4000	1	0.00
CG321 OG303 PG1 NG2R52	0.2600	2	0.00
CG321 OG303 PG1 NG2R52	0.3500	3	0.00
CG321 OG303 PG1 OG2P1	0.1000	3	0.00

TABLE S2 Overview of MD Simulations

System	Label	Simulation time	Simulation Type	Size (# atoms)	System Size (nm^3)	Number of Ions (Cl/Mg^{2+})	Force Field/Method
**3′-OH w/o Mg^{2+} Major Groove**	Sim1a-e	1,005 ns	EQ	34,958	7.2 × 7.2 × 7.2	64/42	CHARMM
**3′O^- w/o Mg^{2+} Major Groove**	Sim2a-e	1,005 ns	EQ	34,957	7.2 × 7.2 × 7.2	64/42	CHARMM
**3′-OH w/o Mg^{2+} Minor Groove**	Sim3a-e	1,005 ns	EQ	34,958	7.2 × 7.2 × 7.2	64/42	CHARMM
**3′O^- w/o Mg^{2+} Minor Groove**	Sim4a-e	1,005 ns	EQ	34,957	7.2 × 7.2 × 7.2	64/42	CHARMM
**3′O^- Mg^{2+}@S_P, 2-NH_2-Im:R_P**	Sim5a-e	1,005 ns	EQ	35,025	6.8 × 7.1 × 7.3	65/43	CHARMM
**3′O^- Mg^{2+}@R_P, 2-NH_2-Im:R_P**	Sim6a-e	1,005 ns	EQ	35,025	6.8 × 7.1 × 7.3	65/43	CHARMM
**3′O^- Mg^{2+}@R_P, 2-NH_2-Im:S_P**	Sim7a-e	1,005 ns	EQ	35,025	6.8 × 7.1 × 7.3	65/43	CHARMM
**3′O^- Mg^{2+}@S_P, 2-NH_2-Im:S_P**	Sim8a-e	1,005 ns	EQ	35,025	6.8 × 7.1 × 7.3	65/43	CHARMM
**3′-OH w/o Mg^{2+} Major Groove**	Sim9	203 ns	EQ	34,958	7.2 × 7.2 × 7.2	64/42	AMBER
**3′O^- Mg^{2+}@S_P, 2-NH_2-Im:R_P**	Sim10	101 ps	QM/MM	35,025	6.8 × 7.1 × 7.3	65/43	CHARMM/HF-3c
**3′O^- w/o Mg^{2+} Major Groove**	Sim11	10 ps	QM/MM	35,023	6.8 × 7.1 × 7.3	63/42	CHARMM/HF-3c
**3′O^- Mg^{2+} Removed Major Groove**	Sim12	19 ps	QM/MM	35,023	6.8 × 7.1 × 7.3	63/42	CHARMM/HF-3c

SUPPORTING REFERENCES

(1) Altona, C.; Sundaralingam, M. Conformational Analysis of the Sugar Ring in Nucleosides and Nucleotides. New Description Using the Concept of Pseudorotation. *J. Am. Chem. Soc.* **1972**, *94* (23), 8205–8212. https://doi.org/10.1021/ja00778a043.
